# Recent Advances of Polymeric Membranes in Tackling Plasticization and Aging for Practical Industrial CO_2_/CH_4_ Applications—A Review

**DOI:** 10.3390/membranes12010071

**Published:** 2022-01-05

**Authors:** Farahdila Kadirkhan, Pei Sean Goh, Ahmad Fauzi Ismail, Wan Nurul Ffazida Wan Mustapa, Mohd Hanif Mohamad Halim, Wei Kian Soh, Siew Yean Yeo

**Affiliations:** 1 Advanced Membrane Technology Research Centre (AMTEC), Faculty of Chemical and Energy Engineering, Universiti Teknologi Malaysia, Johor Bahru 81310, Malaysia; peisean@petroleum.utm.my (P.S.G.); afauzi@petroleum.utm.my (A.F.I.); 2 Gas Sustainability Technology (GaSTech) R&D Department, Group Research & Technology (GR&T), Project Delivery & Technology (PD&T), PETRONAS Research Sdn Bhd, Block E, Lot 3288 & 3289, Off Jalan Ayer Itam, Kawasan Institusi Bangi, Kajang 43000, Malaysia; nurulffazida@petronas.com.my (W.N.F.W.M.); hanifhalim@petronas.com.my (M.H.M.H.); weikian.soh@petronas.com.my (W.K.S.); siewyean.yeo@petronas.com.my (S.Y.Y.)

**Keywords:** CO_2_ removal, plasticization, aging, contaminants, polymeric membrane, mixed gas

## Abstract

Membranes are a promising technology for bulk CO_2_ separation from natural gas mixtures due to their numerous advantages. Despite the numerous fundamental studies on creating better quality membrane efficiency, scaling up the research work for field testing requires huge efforts. The challenge is to ensure the stability of the membrane throughout the operation while maintaining its high performance. This review addresses the key challenges in the application of polymeric technology for CO_2_ separation, focusing on plasticization and aging. A brief introduction to the properties and limitations of the current commercial polymeric membrane is first deliberated. The effect of each plasticizer component in natural gas towards membrane performance and the relationship between operating conditions and the membrane efficiency are discussed in this review. The recent technological advancements and techniques to overcome the plasticization and aging issues covering polymer modification, high free-volume polymers, polymer blending and facilitated transport membranes (FTMs) have been highlighted. We also give our perspectives on a few main features of research related to polymeric membranes and the way forwards. Upcoming research must emphasize mixed gas with CO_2_ including minor condensable contaminants as per real natural gas, to determine the competitive sorption effect on CO_2_ permeability and membrane selectivity. The effects of pore blocking, plasticization and aging should be given particular attention to cater for large-scale applications.

## 1. Introduction

CO_2_ has been highly emitted to the environment, with the distribution of 25% being from electricity and heat production, 24% from agriculture, forestry, and other land usages, 21% by industry, 14% from transportation, 10% by other energies, and 6% from building [1]. Natural gas is the lowest-carbon-emitting fuel compared to crude oil and coal [2], where the CO_2_ emission is less by ~26% with the same amount of energy produced [3]. Despite this, CO_2_ removal from natural gas is needed due to issues such as corrosion, catalyst poisoning and its effects on lowering the heating value of fuel [3,4,5,6,7]. Several mass-transfer technologies can be used for this purpose, namely, membrane, absorption, adsorption and cryogenic [8]. However, membrane-based separation methods are more energy-efficient than heat-driven separations. Membrane-based separation uses 90% less energy than its distillation counterpart [9]. The traditional method for CO_2_ capture is based on amine absorption, but this process requires high energy, incurs high capital and operating cost, and poses corrosion and environmental issues in addition to some operational issues [3,10,11]. The membrane has been found to reduce the energy cost of CO_2_ separation from amine absorption and adsorption [12]; hence, it has been vastly studied for this application.

Membrane units are significantly smaller in size than the conventional plants at the same capacity, hence they are suitable for offshore natural gas application [13]. Membranes have been used for industrial gas applications for over 35 years since the first membranes were commercialized for natural gas CO_2_ removal in the 1980s [14]. Since then, the continuous evolution of gas-separation membranes has produced USD ~1 billion in sales. It is estimated that worldwide membrane market growth is very steady, at approximately 10 to 15% per year [15], and 60% of the membrane gas separation market has been dominated by nitrogen removal from air and natural gas CO_2_ separation [16]. At present, the rapid growth of the membrane market is being driven by technological advancements in polymer science and membrane process engineering [17]. Table 1 lists the polymeric membrane producers for CO_2_ separation. 

The potential of polymeric membrane technology as a separation system for specific high CO_2_ gas fields, when compared to other competing solutions, depends on the feed flow, operating conditions, the target purity and location of the project [18,19,20]. The membrane offers great advantages as a green technology due to its lower energy consumption, no chemical solvent usage, simple operation and maintenance, and high reliability. In addition it is modular, hence easy for expansion, it being energy-efficient, and it have a compact module design [6,11] which is crucial for offshore operation. On top of that, the membrane process response time is basically instantaneous, hence troubleshooting can be very quick, and the rapid start-up can be achieved [20]. Membrane-based gas separation favours bulk removal when only modest purity is needed and the source feed gas is at high pressure, while the retentate stream is the sales gas product [21], at high pressure for the ease of transport.

Several large-scale membrane separation processes have already been installed at offshore platforms with [22] the CO_2_ contaminant in the range of 5 to 70% in the crude natural gas [17]. Depending on the target, a certain percentage of the hydrocarbon loss is required from the membrane to meet the flare BTU (British Thermal Unit) number to avoid using sales gas as the flare fuel gas. In this case, polymeric membrane possesses high productivity, and lower purity is required for separation, so membrane area and cost could be reduced. Some might target very stringent outlet CO_2_ percentages depending on the pipeline specifications. As an example, U.S. pipeline specifications are <2% CO_2_, 7 lb H_2_O/MMscfd, 4 ppm H_2_S, dew point of <−20 °C and heating value of 950–1050 Btu/scf [23]. Unfortunately, there are only few polymers that have both high permeability and selectivity since most polymeric materials have a trade-off relationship according to the Robeson plot [24]. A commercially attractive membrane must possess excellent separation efficiency with high permeance and selectivity, be stable for long-term operation duration, have high strength, and be able to withstand high temperatures and various contaminants. It also needs to have high resistance to aging and plasticization as well as being easily and cheaply manufactured into different sizes and shapes of membrane modules [25].

Modifications to the membrane, such as adding additive, cross-linking, and many others, cannot significantly address the membrane performance trade-off bottleneck between selectivity and permeability. It is not easy to improve both productivity and purity due to the trade-off characteristic found by Lloyd Robeson [26,27]. A study by Lam et al. [28] found this trade-off relationship in the effort to improve CTA polymer performance by adding ionic liquid. It was hypothesized that the ionic liquid can reduce the polymer crystallinity and enhance the CO_2_ affinity. The resulting membrane results in increased CO_2_/CH_4_ solubility selectivity, but CO_2_/CH_4_ diffusivity selectivity is reduced. Another example is the polyacetylenes, which have very high CO_2_ permeability but very low selectivity. Their selectivity can be improved significantly by replacing some pendent groups, however this is accompanied by some reduction in permeability. In addition, rapid physical aging significantly decreases the permeability [24].

The gas-separation membrane working principle is based on the difference in the diffusivity and solubility of some gases through a polymeric membrane [29]. Polymer materials are more rigid, hence more favourable for application in gas separation since they experience less aging. This makes them more stable for long-term operation [30]. Expanding the performance range, gas transport properties and improving the membrane robustness to plasticization, aging and degradation of performance over time are the major goals of all polymer material scientists and engineers working in this field [31]. A membrane with a higher plasticization pressure is required since it can sustain a high selectivity value when applied to high CO_2_ feed concentration and high operating-pressure applications [32,33,34]. The investigation into novel membranes will focus not only on more selective membranes but also on highly plasticization-resistant membranes with stable flow for optimum membrane replacement rate and less requirement for comprehensive pretreatment systems [35]. High-pressure gas permeability typically does not follow the dual-mode sorption model, since polymer chains are subjected to plasticization [36].

Under single-gas testing, numerous materials are able to achieve the desired selectivity. However, when tested with multicomponent feed in the actual plant separation processes, the separation performance of membranes severely reduces. Usually, competitive sorption, plasticization and aging phenomenon are responsible for this performance deterioration [37]. Currently, most of the studies on mass transport focus on single gases and conclude on the membrane performance using this information. This approach might result in misleading conclusions (overestimation or underestimation) due to the absence of contaminants’ and multicomponent gas’ effects, which will reduce the pure gas performance significantly [38]. A good membrane performance is measured by having less hydrocarbon loss, which can be attained by maintaining high selectivity throughout the operation. In addition, a reduction in membrane area by >40% can be achieved by upholding stable permeability during the membrane’s life [39]. Higher permeability leads to a smaller membrane area needed for the same gas capacity, hence fewer membrane modules and lower capital cost are needed [40].

In 2016, Suleman et al. [22] reviewed the plasticization phenomena in CO_2_ removal from natural gas covering inorganic, polymeric membranes and mixed-matrix membranes. Low et al. [16] reviewed the physical aging of high free-volume glassy polymers and its prevention methods. Later, Zhang et al. [3] reviewed approaches to reducing plasticization specifically for polyimide (PI) membranes, focussing more on various cross-linking methods. By emphasising the practical industrial application, the present review provides the state-of-the-art scientific and technological advancement in producing plasticization- and aging-resistant polymeric membranes, covering various existing commercial polymeric membranes, their modification, and new novel polymers. The glassy polymeric membrane plasticization and aging mechanisms and their impacts on gas separation are reviewed. Particular attention is paid to the key challenges in the application of polymeric technology, and the potential techniques that have been explored to overcome the issues are also discussed.

## 2. Commercial Polymeric Membrane for Gas Separation

For natural gas separation systems using glassy polymeric membranes, cellulose and sulfone-based materials are the pioneers used for commercial membranes. Although these polymers still dominate the market, more polymers such as polycarbonates (PCs), polyimide (PIs), sulfonated polysulfone (sPSF), polyetherimide (PEI), and polyamides (PA) have also been introduced commercially [31]. Reported CO_2_ pure gas permeabilities and CO_2_/CH_4_ ideal separation factor at 35 °C and 2 atm for PSF are 5.6 Barrer and 22, for cellulose acetate (CA) they are 6.6 Barrer and 33, for PIs they are 7–20 Barrer and 58 to 102, while for tetrabromo-PC they are 4.2 Barrer and 32, respectively [41]. The features of the polymeric membrane affect the attraction of various gas molecules within the membrane differently, which affects the gas solubility. Membrane fabrication will influence the polymer structure matrix, and the amount of free volume will influence diffusivity [11]. The diffusivity–selectivity can be boosted by enhancing free volume and by modification of its distribution, while the solubility can be improved by introducing CO_2_-philic groups [24]. The incorporation of various Lewis bases, such as ether and carbonyl oxygen, promotes physical interaction due to higher polarity, thus producing higher CO_2_ solubility [17].

Many high-performance polymers have been formulated, but very few can be applied commercially, mainly due to physical aging and plasticization issues [25]. Traditional glassy gas-separation membrane polymers have low free volume, such as CA, PI and PSF. Hence, they possess low gas flux and high gas-separation factors during high-pressure applications. On the other hand, rubbery membranes such as poly(ethylene oxide) (PEO), poly(amide-6-b-ethylene oxide) (Pebax^®^), poly(dimethyl siloxane) (PDMS), polyvinyl alcohol (PVA), and polyvinylamine (PVAm) have lower selectivity than glassy membranes despite having high permeability [15], which limits their applicability in gas separation membrane application. Polymer structural characteristics are governed by their physical and chemical properties. Side groups, molecular weight, the polymer chain’s flexibility, chain interactions and Tg (glass transition temperature) are characteristics that impact the performance efficiency of the membrane, including its chemical, thermal and mechanical stability. The nature and number of side groups in the polymer chain are linked to the rigidity percentage of the main chain. Functional groups such as heterocycles, aromatics, and long side chains, can decrease the polymer chain’s flexibility. The availability of nitrogen and oxygen attached to the main-structure carbon atom enhances the chain flexibility. The introduction of aromatic groups into the main chain of the polymer results in flexibility reduction due to the decrease in chain rotation. This leads to chemical and thermal stability, a higher glass transition temperature and a chain rigidity characteristic [42]. The more flexible the polymer chains, the higher the permeability, since the chain dynamic can affect the transport properties and the separation performance, but this is usually coupled with robustness and durability issues. Polymer Tg and molecular weight can be related to polymer interchain forces and interactions between the chain, which define the polymer state [4].

### 2.1. Cellulose Acetates (CA)

Semicrystalline CA is a green material that is relatively cheap because its raw material comes from a variety of renewable resources such as recycled paper, cotton, sugarcane, and wood [11]. The pioneer in commercial membrane applications for natural gas processing is based on CA and its derivatives. Since the mid-1980s, CA for reverse osmosis membranes has been modified and commercialized for CO_2_ separation from CH_4_ [14]. Cellulose tri-acetate (CTA) membranes occupy 80% of the global market for natural gas removal because of their peculiar properties of low cost, ease to process, higher durability, long term reliability, high pressure resistance, and competitive gas-separation performance [43]. CA polymers with different degrees of acetylation can be dissolved using organic solvents, since acetylation significantly decreases hydrogen bonding, enabling them to be fabricated using the phase-inversion process. Its membrane permeability is directly related to the acetylation percentage. Enhancing the acetylation (1.75 to 2.85), improves its CO_2_ productivity to 6.56 from 1.84 Barrer, while maintaining the CO_2_/CH_4_ separation factor in the range of 33 to 35. This enhancement results from the change in polar hydroxyl groups (-OH) to bulky acetate groups (-COCH_3_) (refer to Table 2a), which reduces its density hence generating more free volume [23]. The different steric hindrance and electronegativity of hydroxyl (HO-) and acetate (CH_3_COO-) groups in CA affect the volumetric, mechanical, and gas-separation properties [44].

**Figure 1:**
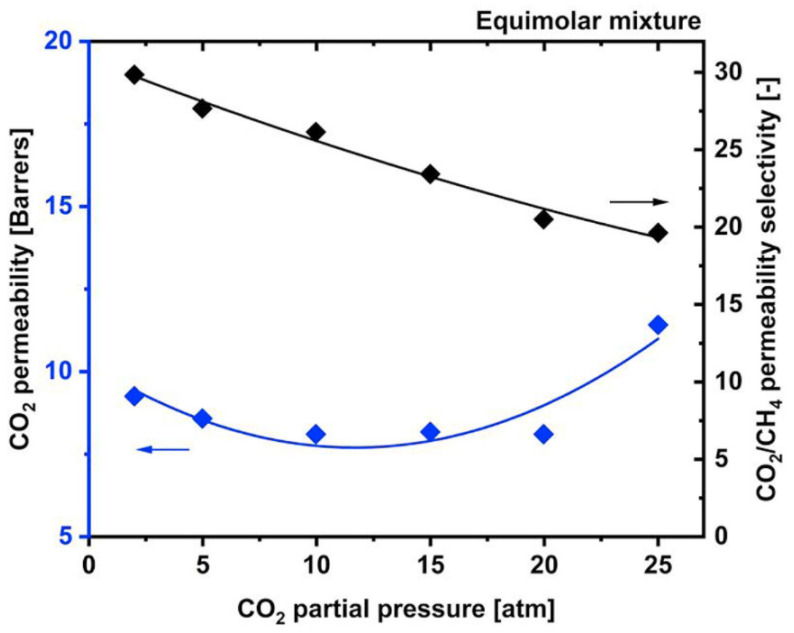
CA membrane performance trend for CO_2_/CH_4_ mixed gas [47].

The major limitation of CA is its susceptibility towards CO_2_ plasticization [23], vulnerability to the presence of water due to its hydrophilicity, and having some interactions with some hydrocarbons such as acetone and aromatics [11], which will damage and dissolve the membrane. Their CO_2_/CH_4_ selectivity reduces to <20 under high pressure of >25 atm and mixed-gas conditions due to a plasticization effect, as shown in Figure 1, which leads to excessive methane loss [41,46]. Eliminating all these contaminants will prolong membrane lifespan. Nevertheless, it is also claimed that CA membranes are robust to aromatic hydrocarbons [14]. CTA can maintain its high performance under actual operating conditions for separation with heavy hydrocarbons due to its high CO_2_/CH_4_ solubility selectivity, which may not be affected by the plasticization induced by the heavy hydrocarbons. The high CO_2_/CH_4_ solubility selectivity in CTA can be further improved by lowering the operating temperature, though the polymer plasticization by the heavy hydrocarbons becomes more significant due to their increased solubility at lower temperatures [28].

CO_2_ plasticization pressure of CA is not changed with different degrees of acetylation. Sanders et al. [23] mentioned that CO_2_ changes the structure of the more highly acetylated membrane more significantly, as shown in hysteresis studies, due to the higher CO_2_ solubility. Compared to the hydroxyl–hydroxyl, the intermolecular network that grips the available polymer chains is lesser in the acetylated membrane, therefore the effort to improve the permeability results in lower strength. Some studies harnessed the benefit of the controlled plasticization effect to enhance the FFV (fractional free volume) of the membrane during fabrication. While CO_2_ is plasticizing the membrane, it modifies the area between CA chains to locate more gas. After the CO_2_ is permeated, the polymer chain will maintain in this new transformed state. The membrane properties due to this conditioning are time-dependent, with more pronounced effects happening with longer membrane exposure to the high-pressure CO_2_ [14]. Cynara^®^ hollow fibres (Schlumberger™, Houston, TX, USA) and Separex^®^ spiral-wound (UOP, Honeywell™, Des Plaines, IL, USA) are commercial CA-based membranes [47], that have been used in high CO_2_ concentration offshore operations.

### 2.2. Polysulfones (PSF)

A basic PSF unit consists of a sulfonic group, an ether group, one or more aryl groups, and other functional groups, as illustrated in Table 2b. This thermoplastic polymer has high mechanical properties, remarkable thermal characteristics with a high glass transition temperature, is stable in oxidizing environments, and has a higher plasticization pressure (~27 bar partial pressure of CO_2_). It also has significant resistance to acid and base environments in specific concentrations and temperatures [48]. PSF has low free volume, less regularity, lower bulky density, resistance to molecule rotation, a rigid backbone, and has high heat-deflection temperature [23]. In 1979, Monsanto fabricated the first membrane separation system using PSF hollow fibres [39], and it was later commercialized for water removal by Air Products, under the trade name ‘Prism’ [49].

Researchers also favour polyethersulfones (PES) which have linear and shorter chain structures, as shown in Table 2c, where the tighter structure yields higher selectivity than PSF. In addition, PES has higher temperature resistance, allowing it to be applied continuously at a higher operating temperature of 200 °C without causing serious damage to its properties. Ether–oxygen, in its structure, provides CO_2_ binding. However, PES has low permeability and a faster aging rate, making it unsuitable for commercial use [30,50]. To resolve these issues, substituting phenyl rings with symmetric bulky groups such as methyl could effectively enhance its gas permeability. Bulkier groups, namely hexafluoro isopropylidene, are used to substitute the isopropylidene bridging moiety to enhance its free volume, making the polymers much more permeable [23]. A novel PES has been manufactured by substituting one diphenyl sulfone unit in the main chains with a bulky and rigid 1,2,4-trimethylbenzene moiety, as shown in Table 2d. The new TPES (poly trimethyl phenylene ethersulfone) has a superior permeability and higher aging resistance. The addition of this moiety creates additional free volume, restricting chain movement and relaxation, hence it displays a slower aging behaviour. The TPES membrane is said to have similar gas-separation efficiency to Matrimid [30].

### 2.3. Polyimides (PIs)

Pis have been proven useful as gas-separation membranes because of their outstanding combination of properties such as unusually high permeabilities and permselectivities. Structure modifications have been made extensively with the incorporation of both aliphatic and aromatic moieties, to enhance its solubility [27] in typical membrane casting solvents. Certain members of the PI family are resilient to chemical attack and stable to temperatures above 400 °C [51]. Table 2e,f,h shows some imide polymer structures prepared via the condensation reaction of an aromatic anhydride group with an aromatic amine [45]. CO_2_/CH_4_ and CO_2_/N_2_ separation can be effectively accomplished using PI membranes [25]. PI membranes possess higher permeability and selectivity than CA membranes, with a 40% area reduction and 75% improvement in CO_2_ removal [49]. However, like CA, an imide-based material called Matrimid may plasticize and undergo physical aging when applied to feed with condensable gas such as CO_2_. On top of its superior performance beyond the 2008 Robeson plot, the 6FDA-PI has an elongation break, and a Young’s modulus value lower than CA. It has been hypothesized that there is a trade-off trend among gas transport and mechanical properties [39]. Even though Pis have high performance and rigid polymer backbones, they are severely attacked by plasticization of CO_2_ and trace heavy hydrocarbon components at high pressure [35]. Some studies indicate that Pis are more susceptible to plasticization compared to CA, since they are more sensitive to structural changes resulting from their more ordered structure. To ensure acceptable membrane life, extensive pretreatment processes are needed, since it is affected by aromatic hydrocarbon even at low concentrations. It has been found that the separation performance of Matrimid 5218 reduction between single-gas testing and binary gas is larger than CA, which is 33% for CO_2_ permeance and 20% for selectivity (10 mol% CO_2_/90 mol% CH_4_, 7.5 bar and 35 °C). In another study, when the feed pressure is increased from 5 to 50 bar, selectivity of Matrimid declined by ~45% in a 55 mol% CO_2_/45 mol% CH_4_ mixture [41]. This is why CA retains its presence in the market despite its low performance, in addition to its cheaper cost [14]. Several types of treatments such as cross-link, blending, semi-interpenetrating network, and thermal rearrangement have been performed for different PIs in order to increase the plasticization pressure [3].

In addition to the PI membrane’s performance deterioration under mixed-gas conditions, its polymer material is relatively expensive, thus making it less attractive for commercial application [52]. The advancement in composite spinning technology has helped in the economical production of these membranes, where it can be applied in very small amounts as a skin on top of cheaper support material [49]. It has been stated in many works of literature that the next most potential polymer material that can be commercialized to replace the lower performance of CA membrane for gas separation is the improved and modified PI-based material. Those imides which incorporate the -6FDA group possess significantly high CO_2_ permeability values [25]. Table 2h illustrates some of 6FDA-based PIs. PIs such as 6FDA-DAM:DABA 2:1, Matrimid^®^, and Ultem^®^ have plasticization pressures of 11, 12 and 28 bar, respectively. Torlon^®^ has a higher plasticization resistance, where CO_2_ permeance can be maintained almost constantly up to ~84 bar, due to the establishment of N–H groups hydrogen bonding with either N–H or C=O groups [25], as shown in Table 2g.

### 2.4. Polycarbonates (PCs)

PC-based membranes are advantageous for gas removal because of their shape and size-selective properties. At severe temperature and pressure conditions, the mechanical and chemical stability of the membrane is questioned [53]. Hence, this polymer needs to be modified. The substitution of many functional groups with aromatic hydrogens is the most-used modification method [23]. The chemical structure of PC is shown in Table 2i. Tetrabromo bisphenol A-PC (TB-BisA-PC) has been used for gas-separation applications by Innovative Gas Systems (IGS) or Generon. It is the most selective polymer available for air separations, with O_2_/N_2_ selectivity and O_2_ permeability of 7.5 and 1.83 Barrer, respectively [23]. Its four hydrogen atoms in the benzene ring are substituted in proportion by bromine atoms. The incorporation of stronger cohesive forces from polar halogens leads to more compressed packing and lessens the mobility, which is believed to be essential in polymer gas diffusion. The commercial TB-BisA-PC is anticipated to be a polyestercarbonate copolymer, where the polymer backbone comprises ester and carbonate linkages [23]. Among the tetra-substituted PCs, TB-BisA-PC has the lowest free volume, the highest Tg, density, diffusion, and gas permeability [23].

Based on the four main types of commercial polymers described above, each of them has its own advantages and limitations. More works need to be conducted to modify the polymers to cover and improve the limitations for more successful applications in the field with long-term stability. For CO_2_/CH_4_ removal from natural gas at high pressure, improvements needed specifically for the CA type are to improve on its selectivity and CO_2_ plasticisation pressure. For PSF, improvements are needed on its permeability and robustness towards heavy hydrocarbon and aromatics. For polyimides, improvements are needed on its CO_2_, heavy hydrocarbon, and aromatic plasticization- and aging-resistance. For polycarbonate, improvements are needed regarding its selectivity and resistance towards heavy hydrocarbons and aromatics.

### 2.5. Transport Mechanism for Gas Separation Membrane

There are five mechanisms of transport for gas-separation membranes, namely solution diffusion, Knudsen flow, surface diffusion, capillary condensation and molecular sieving. Solution diffusion is the one applied for dense membranes and thin skin selective layers (skin thickness between 0.1 to 1 μm) of asymmetric glassy gas-separation membranes. Diffusion happens in order to attain equilibrium, from higher concentrations to lower ones, and this leads to system entropy increases. The thermodynamic differences between the two membrane sides serve as the driving force [49].

One parameter that associates with the variations in polymer gas transport is the FFV. This represents the open volume in polymer chains for gas molecules to pass through [54]. Glassy membranes are used below its glass transition temperature, hence the polymer chains are improperly packed, leading to space between them [49]. Changes in polymer structure do not consistently affect gas transport behaviour but can significantly influence solubility and diffusivity by changes in the Tg and FFV. Diffusion coefficients decrease with increasing penetrant size, while solubility is related to the condensability of the penetrant [54]. In order to separate a component in a particular feed mixture, the polarity of one component must be close to the polarity of the membrane. Polarity is one of the polymer properties that contributes considerably to the membrane separation performance specifically via solubility factor, which is very dependent on the nature of the polymer [49].

Gas solubility within the rubbery polymer matrix, follows Henry’s Law and is linearly proportional to the partial pressure, or fugacity. Dual-mode models and partial/total immobilization models represent the gas transport behaviour in glassy polymers below Tg [49]. The dual-mode sorption parameters relate to several factors, namely the Tg, FFV and gas permeability, which is the diffusivity and solubility. The free volume is related to nonequilibrium structure existing in the polymer, formed during the membrane fabrication. As the temperature is increased, the excess free volume which is in the nonequilibrium state increases, and the Tg increases. The higher the free volume, the higher the gas solubility for the particular polymer and the higher Tg, and the solubility of condensable gas also increases exponentially [55]. At low vapour activity, dual-mode sorption occurs, where hydrocarbons are adsorbed into the polymer matrix. At more than 0.5 vapour activity, higher permeability has been found to be related to dilation and plasticisation of the polymer. Plasticisation has been found to happen at the particular plasticizer amount for specific polymers. The reduction in gas permeability might initially be partially due to “pore blocking”, “competitive sorption” or “anti-plasticisation” phenomenon, where the free space is occupied by hydrocarbon and aromatics such as toluene, which obstruct the flow path [43]. In the partial immobilization model, it was assumed that not all gas molecules sorbed are mobile and contribute to the gas transport. This model also includes the term to account for competitive sorption [56].

Advanced dual-mode sorption models such as the Flory–Huggins and Guggenheim–Andersonde–Boer model have been established [43]. The simplistic membrane model that does not account for such complex effects can deviate more than 20% from a more rigorous approach. Miandoab et al. [56] has developed a mathematical model for membrane performance that incorporates fugacity-dependent permeabilities, competitive sorption, penetrant blocking and plasticization effects. The model also considers nonisothermal operation and includes real gas behaviour and concentration polarization. Importantly, the model simultaneously considers plasticization caused by water vapor (H_2_O) and carbon dioxide (CO_2_).

Temperature and pressure significantly affect the gas transport behaviour in polymers. By increasing pressure, the solubility will also increase, while opposite effect is seen when the temperature is increased. Diffusivity for noninteracting, low-sorbing gases is largely independent of pressure, while for highly sorbing gases, the diffusivity is dependent on pressure [54]. The factors affecting the gas-separation membrane transport, including the gas and polymer properties with the operating conditions, should be carefully evaluated to attain stable performance of polymeric membranes for real applications.

## 3. Challenges for Gas-Separation Membrane

Laboratory assessment of membranes is frequently performed using single or binary mixtures, hence membrane separation efficiency in actual conditions with multicomponent mixtures is not fully understood [57]. In the real condition, the membrane will undergo competitive sorption, plasticization and aging, which decline its performance and lifetime.

### 3.1. Plasticization Phenomena

Membrane intrinsic mobility and chain packing are susceptible to alteration under harsh testing conditions, resulting in a significant reduction in permeability. Under high pressure, CO_2_ swelling occurs, which consequently increases the segmentation mobility of polymer chains, free volume and interchain spacing [3]. These changes, in turn, induce plasticization and reduce size-sieving ability [58]. The plasticization effect is not instantly recoverable and is sometimes referred to as “conditioning”, which means it is irreversible even after the elimination of the plasticizing agents [16]. Plasticization in the gas-separation membrane is described as physical dissolution by penetrant species in the polymer phase, resulting in swelling due to the polymer molecular chain restructuring caused by plasticizers such as CO_2_ and heavy hydrocarbon gases [49]. The plasticization phenomenon can be observed via obvious loss of selectivity [3] since the permeation of a bigger penetrant is more affected than of the small component [46].

**Figure 2:**
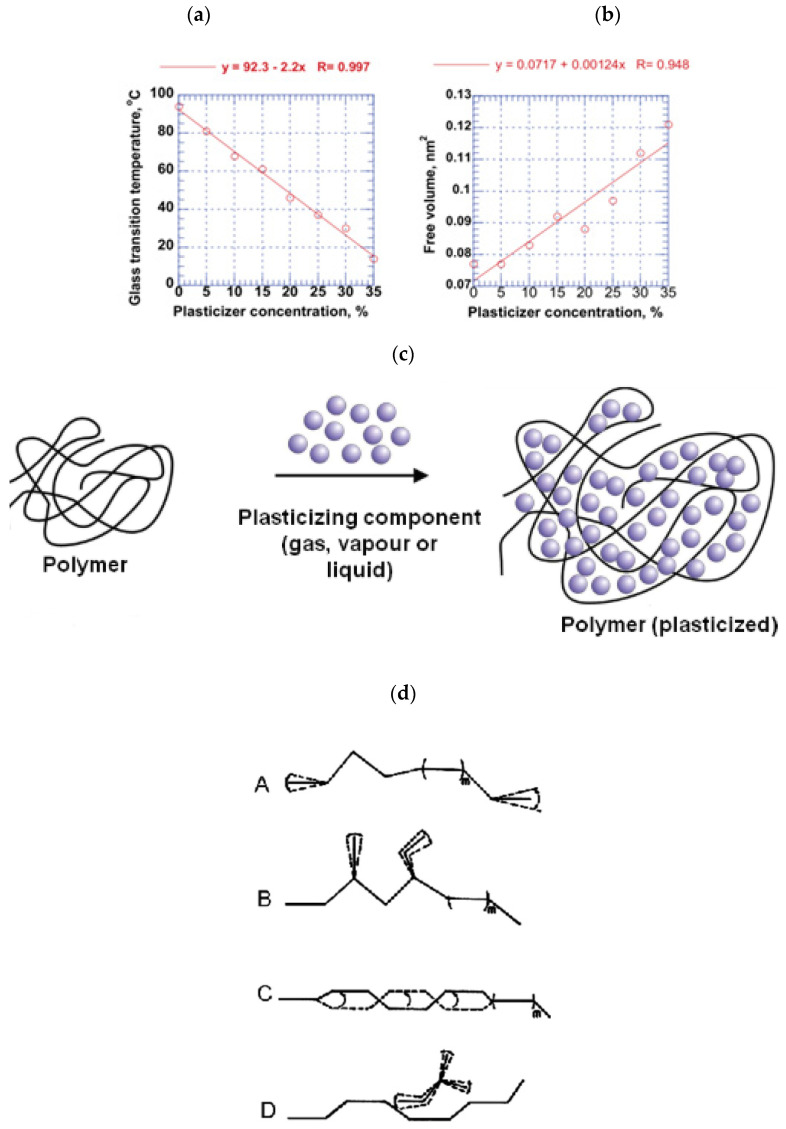
Effect of plasticizer (tricresyl phosphate) on Tg (**a**) and free volume (**b**) of PVC polymer [59]. (**c**)—The plasticization phenomenon in polymers [15]. (**d**)—Free volume for plasticization sources: (**A**) chain end motion; (**B**) side-chain motion, (**C**) main chain “Crankshaft”, (**D**) external plasticizer motion [59].

The early two concepts used to describe the plasticization of polymers are the theory of lubricity and the gel theory. According to the lubricity concept, the function of plasticizer is to reduce friction among the polymer intermolecules. When stretching a plastic, the molecules will flow over each other since the polymer structure is a micellae or a lattice that can rotate and move. The plasticizer serves as a lubricant and reduces sliding resistance. The plasticizer around the polymer chains is probably accountable for the micro-Brownian motion. Typical impacts of plasticization are lowering of the Tg, increasing FFV [27] and elongation, decreasing the tensile strength, ductility, flexural strength and modulus in addition to improvements in material impact strength [59]. The effect of plasticizer on the Tg and free volume of PVC can be seen in Figure 2a,b. From the graph, increasing plasticizer (tricresyl phosphate) concentration up to 35% has reduced the PVC glass transition temperature from 95 to 15 °C and has increased the free volume from 0.072 to 0.116 nm^2^. This can be an example of what is happening to the gas-separation membrane when they meet with the plasticizers in the feed gas stream, such as CO_2_, heavy hydrocarbon, aromatic compounds, H_2_S and many others.

Plasticizer and polymer layers are in an alternate arrangement, as drawn in Figure 2c [59]. The gel theory mentions that the polymers are designed in a honeycomb structure, maintained by wobbly attachments. Solvation–desolvation and aggregation–deaggregation equilibria exist between the polymer and the plasticizer molecules. The polymers’ stiffness is mainly due to its structure’s resistance to internal frictions, as stated by the lubricity theory. According to the gel theory, the number of attachment points between polymer to polymer are reduced by the present of plasticizer, and distortion happen without actually breaking it. A good plasticizing activity includes the filling of the voids, which then acts as a lubricant, forming planes of easy glide. With the application of any type of force, the planes glide over one another [59].

Plasticization can reduce polymer crystallinity and lessens water sensitivity, depending on the type of plasticizer. Plasticizer also can acts as a source of free volume, which will increase gas permeability [59]. This is illustrated in Figure 2d. This effect is based on the hydrophilic or hydrophobic properties of plasticizers, with hydrophilic plasticizers increasing the water intake [27].

The polymer polarities with the plasticizer are considered as the crucial factor that influences the intensity of plasticization and is considered as a case of like dissolving alike. The major factors determining the polymer solvation by plasticizers are the chemical structure and the polarity of their molecules. If the bonds in polymer chains and plasticizer molecules are close in polarity, then the energy of interaction between homogeneous and heterogeneous molecules is nearly identical, and the dissolution takes place. If polymer and plasticizer molecules differ greatly in polarity, then there is lack of compatibility. Plasticizers with polar groups can often be compatible with nonpolar polymers, but only when the content of polar groups is sufficiently small. The compatibility decreases when polar group content increases. Polar plasticizers need to have molecules with an oleophilic end in order to increase compatibility with nonpolar polymers. Nonpolar polymers can dissolve in polar solvents if their molecules have groups capable of polar interaction [59].

Several efforts have been made to find modelling descriptions for the onset of the plasticization effects associated with permeability increase with gas pressure, aiming at a deeper understanding of the mechanism; however, the search for a correlation between the “plasticization pressure” and material properties are still nonconclusive [60]. Kentish et al. [43] attempted to model the plasticization sorption isotherms using advanced dual-mode sorption models such as the Flory–Huggins (FH) model and Guggenheim–Andersonde–Boer (GAB) model, but none are suitable, hence suggesting that a more advanced model with multiparameters is required to simulate this type of behaviour.

**Figure 3:**
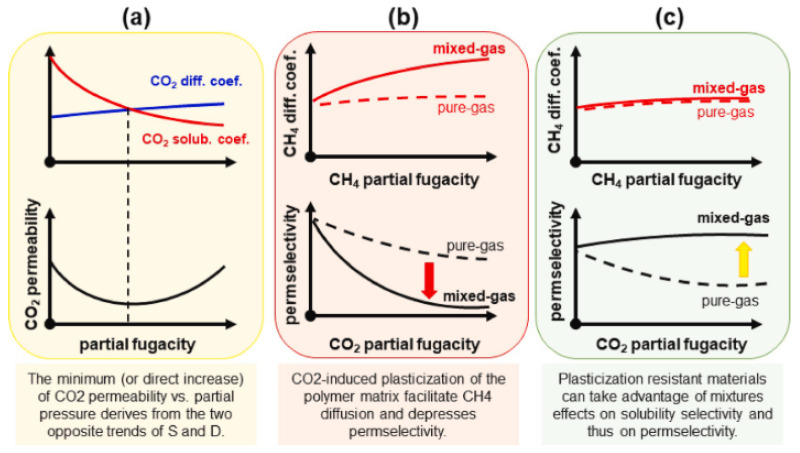
(**a**) CO_2_ permeability of CTA; (**b**) plasticization phenomena (**c**) the behaviour of plasticization resistant polymers [47].

Genduso and Pinnau [47] have conducted some studies on sorption properties and plasticization of CTA. The minimum CO_2_ permeability versus partial fugacity is called the plasticization pressure of the polymer, as shown in Figure 3a. They have found that for mixed gas, CH_4_ solubility is greatly changed by CO_2_ due to the plasticization, while CO_2_ solubility has a slight impact only. This effect will be more severe at higher pressure. CO_2_ dilates the polymer and results in CH_4_ diffusion, which means the size-sieving capability of the CTA has been altered. Therefore, it enhances CH_4_ diffusion and reduces CO_2_/CH_4_ diffusion and permeability selectivity, as shown in Figure 3b [47].

Membranes with plasticization resistance at high pressure need to be explored to improve chain rigidity and swelling resistance, which will not allow the diffusion coefficient of CH_4_ to be enhanced when plasticized [47], as shown in Figure 3c. CTA has higher or superior solubility selectivity and demonstrates the highest affinity to CO_2_. It has lower diffusion selectivity when compared to 6FDA-mPDA PI. CTA’s CO_2_ solubility is only slightly changed by the presence of methane, and competition effects reduce CH_4_ uptake significantly. However, methane diffusivity will be boosted by the CO_2_ swelling [47]. The same finding has been reported using 6FDA-mPDA polyimide material, where the size-sieving properties have been altered when exposed to mixed gas, resulting in a reduction in mixed-gas selectivity due to CH_4_ diffusion change, while the solubility coefficient is improved [61].

#### Plasticizers

Plasticization can have a positive or negative impact, depending on its application. Different types and concentrations of plasticizers can be used in small amounts during polymer processing for specific functions, such as to reduce melt viscosity, increase flowability, reduce processing temperature, flame retardation and smoke reduction. On top of that, it also can serve as a coalescing agent, promote adhesion, influence diffusion coefficient and affect the mechanical features, i.e., hardness, tensile strength, tensile modulus, flexural strength and flexural modulus [59].

**Figure 4:**
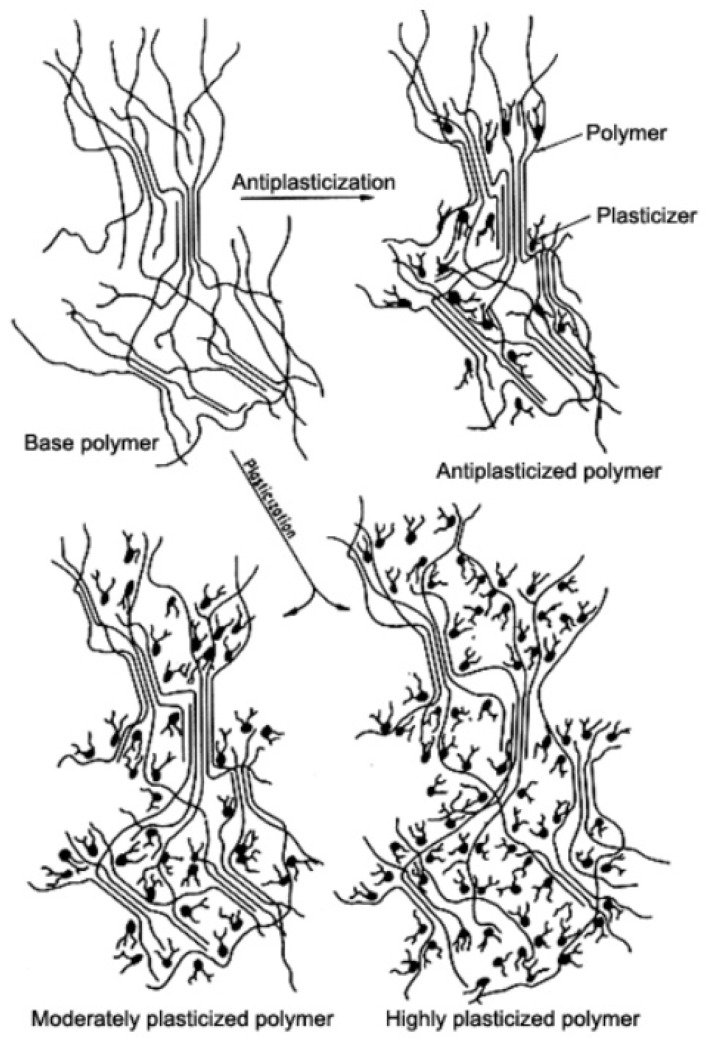
Illustration of antiplasticization and plasticization of a resin [59].

The external plasticizer is usually added to a resin or compound during processing, as opposed to an internal plasticizer which is incorporated in a resin during the polymerization process. Figure 4 demonstrates the idea of resin plasticization, with antiplasticization and plasticization illustrations, where both phenomena can happen depending on the quantity and the type of the plasticizer used [59]. The essential properties of plasticized polymer materials can be related to the type and the amount of plasticizer. The properties influenced include permeation of gases and liquids, sorption of water, hydrophobic or hydrophilic properties, dissolution, and foaming. Gas permeability relies on chain mobility and intermolecular distances (free volume) which can be modified by plasticizers. For example, by increasing the plasticizer content or elevating the temperature, water flux is improved due to the enhanced free volume [27].

Several plasticizer characteristics influence the plasticization behaviours; (1) a plasticizer with a branch which has more free volume is more powerful compared to a linear plasticizer (for the same molecular weight), (2) bigger plasticizer molecular size enhances the polymer-free volume, hence more plasticization occurs, (3) the plasticizer for polymers can also be a small molecule with high free volume, (4) plasticizing efficiency decreases with the increase in plasticizer molecular volume (or weight), (5) lower Tg plasticizer is more effective in lowering the Tg of the plasticized system [59], (6) plasticizers comprising long aliphatic chains are more efficient than those with bulky cyclic groups, but this will be reversed at high temperature, and often the presence of benzene rings in a plasticizer molecule favours compatibility caused by entropy factors [59].

The issue of controlling plasticization at high-pressure operation and its impact on membrane selectivity is one of the most significant material science challenges related to the use of membranes [62] for natural gas sweetening, which is considered not an easy separation [35]. Some of them can be condensed in the system due to Joule Thompson (JT) cooling and act as a liquid plasticizer instead of a gas plasticizer. On the other hand, under controlled plasticization where the polymer can retain the molecular discrimination features within the operating conditions, this condition is favourable since it offers much higher acid gas permeability. In some cases, swelling-induced controlled plasticization of some glassy polymer membranes even offers much higher CO_2_ removal permeability than rubbery polymer membranes [63].

**Figure 5:**
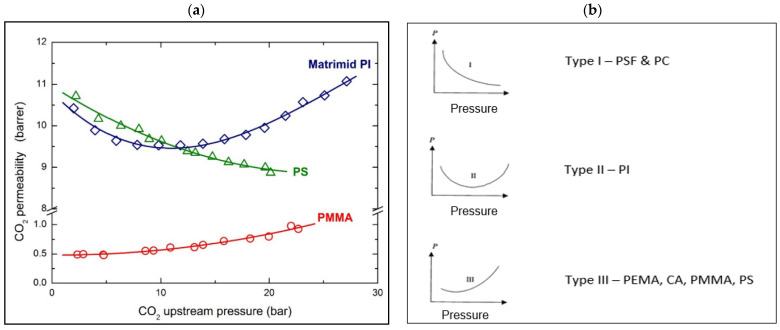
(**a**) CO_2_ permeability in three different glassy polymers at 35 °C vs. pressure [60]. (**b**) Illustration of permeabilities (P) for some glassy polymers to CO_2_ up to 30 bar [65].

The lowest pressure where the permeability-increasing trend starts is named the plasticization pressure. Permeability increment trending from the enhanced in-chain mobility or dilation can be seen by the Tg reduction of the polymer [64]. It is assumed that the polymer requires the equivalent CO_2_ amount to start the plasticization but at a different pressure to reach this concentration, called the plasticization pressure. The accurate calculation to relate the concentration, pressure, and plasticization together will be the partial pressure. CTA has the lowest plasticization partial pressure at 10 bar, and PSF has the highest plasticization partial pressure at 34 bar [64]. A study by Minelli et al. demonstrated that polystyrene (PS), in Figure 5a below, shows a decreasing trend versus operating pressure, which is opposite to the case of polymethyl methacrylate (PMMA), which has an increasing trend even at very low pressure. The CO_2_ permeability in Matrimid PI shows a minimum value at around 11 bar of feed pressure. According to the literature, it can be concluded that PS behaviour does not show any plasticization effect in this range of pressure, which means it has higher plasticization pressure, but PMMA and PI show that plasticization already occurred. In PMMA, the plasticizing phenomenon takes place even at low pressure, while in PI, it takes place only beyond 11 bar [60].

The different trends were due to the difference in Tg, thickness, molecular weight, free volume, permeability mechanism, and many other polymer characteristics [60]. Polymers with the absence of large substituents on the backbone are more rigid, and hence have higher plasticization pressure, such as PSF and PC which are characterized as two examples of Type (I) trending in Figure 5b. Type (1) happens when the diffusion of the polymer reduces with pressure increment, as explained by the dual-mode sorption model, and the decrease in permeability is based on a Langmuir hole saturation theory. At a low concentration of the penetrant, the gas molecules fill in the microvoids [65], or polymer-free volume, until the point where the microvoids turn out to be full at a higher concentration, which is known as Langmuir sites saturation [3]. The higher plasticization pressure of the polymer results in a greater reduction in permeability with increasing pressure [64], due to the site saturation mechanism, before swelling occurs later at higher pressure.

Type (II) demonstrates the permeability trend that is initially declining at low feed pressure, which follows the dual-sorption theory predictions and is deflected when it reach the critical pressure, e.g., PI families [65]. The minimum is commonly associated with a sort of threshold pressure indicated as “plasticization pressure” [60]. In the range of this study up to 30 bar, PSF and PC (Type 1) did not reach its plasticization pressure since it has no deflected point yet, as can be seen in Type II. The plasticization pressure of PSF is 34 bar and for PC is 31 bar which is above the operating pressure range of 30 bar in Figure 5b.

For polymers that have permeability that increases with pressure, it was hypothesized that the diffusion is higher due to swelling induced by plasticization phenomena than the solubility reduction during pressure increment. PEMA, CA, PMMA and polystyrenes (PS) are polyacrylates which have large side groups and display type (III) trend. This is uncommon for glassy polymers, which behave as rubber, where the CO_2_ permeability increases with pressure increment [65]. CA has a plasticization pressure of 11 bar, which is much lower than the operating pressure of 30 bar. At the point of plasticization pressure, where swelling starts to occur, the permeability will increase as the pressure increases. There will be a period of good plasticization effect that will only increase the permeability of the desired component until it reaches the point where selectivity starts to decrease. This is the time where the swelling is enhanced till the undesired component can also pass through, and the membrane has lost its separation ability.

Polymers that have less CO_2_ solubility are less plasticized. However, this theory cannot be generalized [23]. Bos et al. [64] investigated the claim that in the presence of polar and flexible pendant groups, the solubility coefficient for that polymer will be higher. It is assumed to have dipolar interactions with polarizable carbon dioxide molecules, for example, -OCOCH_3_ or COOCH_3_ in PMMA (polymethyl-methacrylate), PEMA (polyethylmethacrylate), and CA. These polar interactions are stronger than chain segments interactions; hence CO_2_ can break these connections. They studied this hypothesis and found no clear connection between sulfone density or carbonyl in several polymers and plasticization pressures. 

Petroleum-based oil, paraffinic, naphthenic, and aromatic oils that in liquid form are plasticizers [59], affect the polymeric membrane. Some polymer material for gas separation is not robust to the condensing liquid which will be created during the gas permeation. Thus, to avoid the liquid formation due to condensation, a prefeed heater is normally installed [66]. Permeation of solvent through a pervaporation membrane is a special case of such a situation [59], since it should not be affected by the liquid feed as per the design concept, hence suitable membrane material selection for this kind of application is crucial.

**Figure 6:**
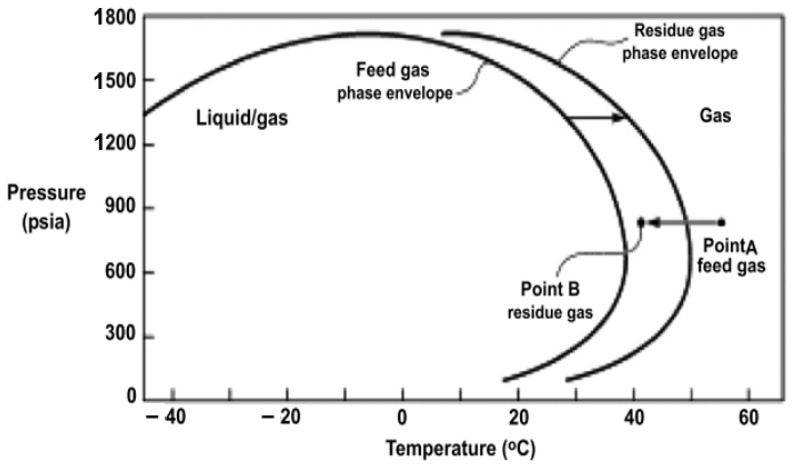
Graph displays the phase envelope change during natural gas processing by a CO_2_-selective membrane [33].

Figure 6 illustrates the phenomenon happening during CO_2_ removal from natural gas using a membrane. Heavier hydrocarbons that cannot be permeated become concentrated in the retentate stream while the CO_2_ and some CH_4_ is taken out in the permeate. The increased concentration of heavy hydrocarbons causes a change of dew point at a higher temperature and brings the curve to the left of the graph. Point A of feed gas (~55 °C) has moved to point B (~40 °C) residue gas. The residue gas in the retentate is then cooled by 10 to 15 °C compared to feed gas temperature due to JT (Joule Thompson) cooling from the expansion of the CO_2_ permeating. The JT cooling and the accumulated heavier hydrocarbons’ concentration in the retentate take the gas into the liquid and gas region (2 phases) resulting from the new, shifted phase envelope [33]. These effects make the residue gas reach the saturation level; hence condensation occurs, resulting in a temperature and slight pressure drop across the membrane [66]. On top of that, condensation swells the membrane material leading to overall separation performance deterioration [49].

### 3.2. Competitive Sorption

Genduso and his team [61] experimented to discuss and clarify the effect of competitive sorption and CO_2_ sorption in 6FDA-mPDA using CO_2_-CH_4_ mixtures. Mixed gas testing has shown a reduction in both CO_2_ and CH_4_ solubility due to the effect of a gas mixture. The CO_2_ versus CH_4_ solubility coefficients trend for mixed gas behaves linearly, similar to other glassy polymers reported in the literature. The mixed-gas CO_2_/CH_4_ solubility selectivity increases with pressure compared to its value at infinite dilution, but the overall mixed-gas permeability selectivity declines from its pure gas value due to contribution from the diffusivity coefficients. The CO_2_ diffusion coefficient is maintained as per the pure gas value, but the CH_4_ diffusion coefficient is enhanced since it has been induced by the CO_2,_ which leads to a reduction in its size-sieving capability.

Similar findings by Ricci et al. [67], mentioning that multicomponent solubility selectivity is higher than the pure gas value, which has a positive impact on separation, as measured in HAB-6FDA polyimide and its thermally rearranged derivative, TR450, at 35 °C and ~30 mol% CO_2_ mixture composition. Unlike in the pure gas experiment, where diffusivity contributes more to the overall ideal selectivity due to the absence of competitive sorption, the multicomponent experiment shows that selectivity is solubility driven. The CO_2_ swelled the polymer, impacted the diffusivity selectivity of the material, and enhanced the CH_4_ diffusion, leading to separation controlled by the solubility selectivity. This is considered as a general trend based on systematic literature data evaluation by materials with very different chemical constitutions. This makes polymers perform better when they are tested with multicomponent, and this fact is to be used for future design and synthesis strategies of the new materials.

In addition, the modelling study has been conducted to investigate the competitive sorption, plasticization and pore-blocking phenomenon for CO_2_ and CH_4_ with the presence of H_2_O. The CO_2_ and CH_4_ fugacity-dependent permeabilities are reduced compared to the pure gas values due to free-volume blocking and competitive sorption by the water vapour. However, due to the small size of the water molecule’s kinetic diameter, it can permeate rapidly, eliminating the diffusivity hindrance induced by its molecule. The humidified and dry feed-conditions simulation differs more at high feed flow rates due to the amount of water retained on the retentate side [56].

**Figure 7:**
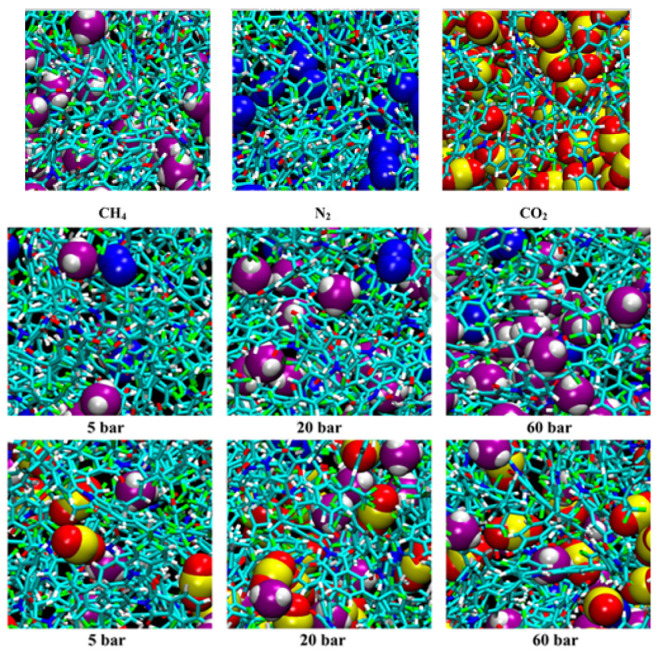
6FDA-6FpDA PI with ~20 bar of methane (C = purple, H = white), nitrogen (N = blue) and carbon dioxide (C = yellow, O = red) (**top row**). 6FDA-6FpDA PI. binary 2:1 CH_4_/N_2_ (**middle row**); 6FDA-6FpDA PI. ternary 16:8:1 CH_4_/N_2_/CO_2_ (**bottom row**) [68].

To understand the effect of competitive sorption, it is further investigated using molecular modelling, where the method to evaluate the gas has been omitted by competitive sorption for the mixture. The gas sorption isotherms in 6FDA-6FpDA PI have been simulated (n_atoms_~50,000), and the inclusion of gas molecule into the chain of polymer were performed using Excluded Volume Map Sampling (EVMS) and validated using lab experiments. The results were directly related to the gas solubilities, with nitrogen having less than methane and methane having less than CO_2,_ as can be seen in Figure 7 (top row). The sorption of the least soluble and lower partial pressure N_2_ was blocked by CH_4,_ which is more soluble and has higher partial pressure. For the 16:8:1 CH_4_/N_2_/CO_2_ mixture, the presence of the highly soluble CO_2,_ even at small concentrations, had a substantial effect on depressing the uptake of both other penetrants, and at this concentration it is not enough to plasticize the membrane, as illustrated in Figure 7 (bottom row) [68].

The binary gas-sorption separation factor varied strongly compared to pure gas, suggesting the presence of competitive sorption. The iterative test particle insertion molecular dynamics (TPI-MD) and grand canonical Monte Carlo molecular dynamics (GCMC-MD) consider the interrelationship of the gas concentrations, solubilities, volume changes, and polymer local relaxation. Therefore, the evaluation of mixed-gas performance can be conducted without pure gas results. Measurements of sorption studied at higher operating pressure and temperature varieties can also be performed without having to perform the complicated mixed-gas testing. Furthermore, the iterative GCMC-MD method should be relevant to perform multicomponent testing, which is important for industrial gas-separation applications [68].

A study on deviation between ideal selectivity (pure gas) and true selectivity (mixed gas) revealed that the difference can be up to factor of four, as the feed pressure changes due to competitive adsorption–diffusion [69]. Heavy/slowly diffusing molecules, in this case CO_2,_ can dramatically affect the diffusion flux of weakly adsorbed/light molecules, such as He. When the partial pressure of CO_2_ increases from 1 to 3 bar, true permselectivity decreased from ~1.6 to ~0.5, while ideal selectivity was insignificantly affected and remained at ~3. However, the permeability of CO_2_ increased by ~30% while He decreased by 60%. It was concluded that competitive sorption (adsorption–diffusion) is a function of a chemical potential, molecular mass, and diffusivity, and is more significant for light species. From this study, the importance of competitive sorption in affecting the membrane’s overall performance is highlighted. The misleading nature of ideal selectivity in presenting the overall membrane separation performance for real applications is also emphasized [69].

### 3.3. Polymer Aging

Glassy polymer chains gradually relax into their favoured higher packing density (densification) due to physical aging and compaction phenomenon [30]. Initially, the permeability will be increased due to plasticization, but after a certain duration, the transport properties will be led by physical aging, causing the permeability to decrease [23]. Physical aging in glassy polymers happens due to the relaxation of the nonequilibrium chain conformation towards an equilibrium state below the glass transition temperature [70]. Polymers experience local movement and are progressively densified, lessening their free volume slowly [39], which starts from storage time even before they are exposed to external force [30]. Hence, proper storage is important to avoid this phenomenon, which occurs rapidly due to exposure to humid air and contaminants. Therefore, the membrane must be put in a sealed bag or desiccator at room temperature during storage [58].

A thin-skin asymmetric membrane has been invented, since studies have shown that skin thickness is governed by material density [46]. On top of that, thin films are essential for membrane applications to enhance gas flux and reduce capital costs [71]. Decreasing thickness means the membrane will have lower FFV, and higher cohesive energy density (CED) leads to higher gas sorption and lower gas mobility [46], but the physical aging of the thin-skin asymmetric membrane is a huge concern. Physical aging takes a long time to severely impact the performance of the thick films (50–100 μm), and in laboratory testing, the effects on gas permeability seems insignificant. However, the effects on the thin membranes (<1 μm) can be seen within a few days, if not hours [39]. The thin film’s accelerated aging is due to the free-volume diffusion to the film surface and higher mobility near the surface. This results in lower FFV in the polymer which occurs more rapidly than in bulk samples [16]. The dense selective layer, although possessing invisible pores, contains less than 3–10 Å in diameter free volume in the interstitial space of polymer chains [72]. Throughout the operation, densification leads to a reduction in these free volumes and the permeability [72]. Therefore, one can use gas permeation results over time to trace the physical aging [46].

Aging happens more quickly in a thin film, as can be seen for traditional polymers (PI, PPO, PSF) and high free-volume polymers (Teflon AF, PTMSP, Hyflon AD, PIM-1). Physical aging is highly thickness-dependent and will show accelerated physical aging rate with decreasing thickness, particularly for those of less than 1 micron [23]. This aging is dominant in the glassy polymers with inefficient chain packing conformation with lots of nonequilibrium, excess free-volume elements surrounding chains, that serve as a precursor for physical aging. Particularly, the high permeability due to high fractional free-volume polymers such as PIM-1 will have shorter membrane life due to aging [58]. Over a long time of exposure to operating pressure, the aging effect will be enhanced, which will finally overrule the plasticization effect [72]. The excess free volume is diminished, hence lowering the permeability value, and diffusivity and solubility [72].

Polymers with different structures, especially at the backbone, age differently. This aging is thermally reversible, hence after a decrease in permeability it can be heated above its Tg to recover the losses [23]. This method only includes recoverable changes in properties and cannot be performed if permanent alteration happens to the structure. The impacts of physical aging on the polymer are different from chemical aging, absorption, degradation or contamination, that might impact the membrane properties more severely as the operation duration is prolonged [16]. Gas permeability measurements for high FFV polymers are usually performed after nonsolvent soaking, either with ethanol or methanol, to readjust the aged membrane properties. Thermal history elimination via annealing above Tg combined with rapid quenching to room temperature is impossible for high free-volume polymers due to very high or immeasurable Tg. This technique is not suitable for thin-film composites as it can cause delamination of the thin film from the substrate [16].

Physical aging alters polymer-specific volume, entropy and enthalpy, in addition to reducing permeability. The physical aging rate will be reduced when the driving force is diminished, when the excess free volume and polymer chain mobility gradually decrease [23]. A study by Yavari et al. [73] found that among the three perfluoropolymers studied, permeability reduction rate due to aging increases with the increment of FFV in the polymer. FFV is directly proportional to the gas permeability but experiences a trade-off with selectivity. FFV, CO_2_ permeability and CO_2_/CH_4_ selectivity for Teflon AF1600 are 0.29, 520 Barrer and 6.5, respectively, while Hyflon AD80 and Hyflon AD40 have lower FFV, lower permeability and higher selectivity. These perfluoropolymers exhibit much higher permeability values than typical hydrocarbon polymers with similar FFV values. This is caused by the side groups (such as –CF_3_) in the perfluoropolymers, which are much bulkier and heavier than those in the hydrocarbon polymers (such as –CH_3_), hence more difficult to relax, leading to a slower aging rate in the perfluoropolymers. In general, physical aging decreases gas permeances, increases selectivity, increases Tg, and it is accelerated significantly for thinner selective layers. A correlation can be made between permeability reduction and increase in Tg, in order to find out the physical aging rate.

Table 3 summarizes the counterbalance effects of plasticization, competitive sorption and aging on the glassy polymer transport behaviour and properties. All can happen simultaneously, and depending on the conditions, one effect can be dominant over others or can be countered by another and stabilize at an average value. Plasticization increases permeability due to the enlarged polymer matrix via swelling, while the competition effect reduces the permeability and hence they balance out each other [74]. This means that plasticization effects are masked by competitive sorption during mixed-gas experiments [75].

High gas permeability and selectivity can be attained by polymer blending [76]. Some studies have involved the modification of free-volume distribution [16], such as blending a slower-aging polymer with a higher-aging polymer to result in a lower average value. Several other design methods have also been proposed to enhance the stability and aging resistance of the new blended membrane. These include enhancing initial gas permeability of the blended membrane by blending combination with a modified membrane with high permeability but similar aging rate. The hypothesis is that when the membrane has high permeability, densification and aging may potentially reduce the flow and reach the stability value at a higher permeability than the commercial membrane. The next method is to blend with a modified membrane that has a slower aging profile. This introduces discriminating aging that enhances overall gas performance, with bigger pores having faster aging than the smaller pores. In addition to that, substitution methods such as using hydroxyl to freeze the free volume via hydrogen bonding and prevent it from collapse can also be effective strategies to prevent a faster aging rate [16].

Lau et al. [77] claimed that the incorporation of ultraporous additives can absorb some polymer chains, preserving the porous and low-density, high free-volume polymers. This froze the chains in their open position, slowing the aging process whilst improving gas-transport properties for permeability and selectivity. This particular microporous-microparticle porous aromatic framework (PAF) will generate an intertwined nanocomposite with the polymers. This results in super glassy polymers’ aging suppression at high CO_2_ flow and CO_2_/N_2_ separation factor, as demonstrated over one-year of testing. They highlighted that this technique could permit high free-volume polymers to be reconsidered for gas separation applications. It is essential to further evaluate the aging factor of the fabricated membrane. There should be minimal loss in terms of selectivity and permeability over a typical operating period with feed gas containing contaminants. The acceptable decline in permeability should be limited to around 20 to 30% throughout the minimum membrane lifetime of about 3 years [78].

Wu et al. [58] introduced a PPO membrane with a new design (venation-like) via several interchain cross-linking bridges by means of Friedel–Crafts-based hyper-cross-linking reaction. This stiffens the chains to avoid densification by inhibiting the microstructural deformation, while producing ultrahigh CO_2_ permeability resultant of higher free volume. After 360 days of testing, only a 19% CO_2_ permeability reduction was recorded, and plasticization is fully suppressed up to a 70 bar operating pressure. Clarizia et al. [79] studied the permeation tests for a long duration on asymmetric PI hollow fibres (HF). They found that aging of the membrane led to dense skin layers, hence a reduction in permeance while enhancing selectivity. The most significant deterioration was noted in the initial six months of aging duration. The HF samples aged differently according to the specific preparation method, and the one which was cross-linked was more stable over time. The thinner skin layer resulted in more aging over time. Alcohol treatment that yielded permeability enhancement also resulted in a gradual permeance reduction over time. Nevertheless, the gas permselectivity was sustained and even increased for some cases.

Further research is necessary to minimize the membrane aging and compaction issues during high-pressure operation, which will reduce the membrane lifetime. Polymer-aging reduction approaches that have been proposed are copolymerization and polymer modification [4], backbone redesign [80], filler incorporation, and polymer blending [71]. On top of that, incorporation of filler in a polymer matrix such as metal–organic framework (MOF) can also decrease the effects of physical aging. Despite all the strategies proposed, long-term accelerated aging studies for the polymer and MMM need to be thoroughly investigated in the laboratory to develop a better theoretical understanding of this phenomenon [71].

## 4. Strategies to Improve Polymeric Membranes towards Plasticization- and Aging-Resistance

The methods to limit the polymer chain enlargement and to reduce plasticization include the use of a long polymer chain with a nonordered structure and a highly crystalline polymer with a high-molecular-weight polymer. Longer polymer chains tend to maintain their structures, whereas the crystalline forces present in the highly crystalline polymer can hold the chains together. These forces prevent the plasticizer from entering the polymer. The plasticizer atoms attract the linear and low crystallinity polymer chain via dipole forces and separate the chains by reducing Van der Waals [59]. Plasticization can also be mitigated by improving polymer hydrogen bonding, performing polymer cross-linking either chemically or thermally, improving polymer stiffness by incorporating parasubstituted rings, introducing bulky side groups to decrease rotational freedom and removing any flexible linkages [25,81]. In higher cross-linked polymers, the primary bond gripped the chain tightly.

For CO_2_/CH_4_ gas-separation applications, there are some physical, chemical, and structural polymer characteristics to be considered as suitable selections for the given operating conditions. Among them are the intrinsic membrane performance [82], polymer Tg, polarity to the CO_2_, the dominant transport mechanism, either diffusion or solubility, plasticization pressure, polymer properties such as hydrophilicity or hydrophobicity, FFV, chemical, thermal, mechanical strength and many others. On top of that, other characteristics to be considered are membrane formulation, which includes polymer molecular weight, polymer concentration, type of solvent and cosolvent, nonsolvent-to-solvent ratio and additive, which will determine the membrane thickness, free volume and structure.

CTA, CA, P84, Matrimid, BPZ-PC and PEI are CO_2_ solubility-dominant polymers, where their solubility is more than diffusivity at their plasticization pressure. Diffusivity-dominant polymers are PSF, PES, BPA-PC, TMBPA-PC and PPO. The polymers commercially used for gas separation, such as CTA and CA, have low plasticization pressure, while PSF and PES have higher plasticization pressure. Based on Bos et al. [64], there are no clear relationships between the Tg or film density and the plasticization pressure. There are also no clear correlations between solubility and plasticization pressure, but there is variation in diffusion coefficient as the diffusivity is impacted more severely by concentration; nevertheless, still no trend was found [64].

### 4.1. Polymer Modification via Cross-Linking

A cross-link refers to the connection of two or more chains to each other via covalent bonding, which often occurs via a chemical reaction or ionic bonds. Cross-linking agents are used so that the movement of the polymer chains, degree of swelling and amount of free fraction volume can be controlled. The intermolecular networks via chemical bonds result in materials with greater resistance to dilation and plasticization [83]. It enhances CO_2_ gas permeability and selectivity, enhances FFV and reduces chain mobility. Depending on its physical and chemical attributes, density and molecular weight, the chain mobility can be altered by the cross-linker [50]. The mechanism of cross-linking, such as esterification, monoesterification, decarboxylation, transesterification reactions, imide ring-opening reactions, and chemical cross-linking using diamino compounds, are some of the methods that have been used successfully to prepare membranes with improved plasticization-resistance properties. Cross-linking can also improve membrane resistance to chemical dissolution and enhance thermal strength [51]. This process significantly affects the physical, mechanical and thermal properties of the resulting polymer [42]. Various methods, such as chemical reaction, annealing, physical networking, semi-interpenetrating network, ultraviolet radiation or other energy sources, can be used for cross-linking [3,4,51]. 

#### 4.1.1. Physical Crosslinking

Wu et al. [58] produced membranes with higher plasticization- and physical aging-resistance, and also high CO_2_ permeability without losing selectivity, where a Friedel–Crafts-based hyper-cross-linking reaction was performed to promote numerous interchain cross-linking bridges. This enhanced the chain’s stiffness, preventing densification and microstructural deformation while producing sufficient free volume for permeation. Superior structural stability and very high CO_2_ permeability (5130 Barrer) were achieved 73-fold higher than a virgin PPO membrane. Plasticization was reduced at 70 bar, and the physical aging was lowered to only 19% after 360 days. This extensively cross-linked structure prevents the polymer chain’s mobility, thus improving physical aging and plasticization resistance. Plasticizers dissolve the crystalline structures of polymers, or separate elements of physical cross-linking, hence the surplus of plasticizers may affect the network and lead to a decrease in elongation [27]. Membrane cross-linking for commercial applications should be performed without compromising the reproducibility of separation performance [39].

#### 4.1.2. Chemical Cross-Linking

**Figure 8:**
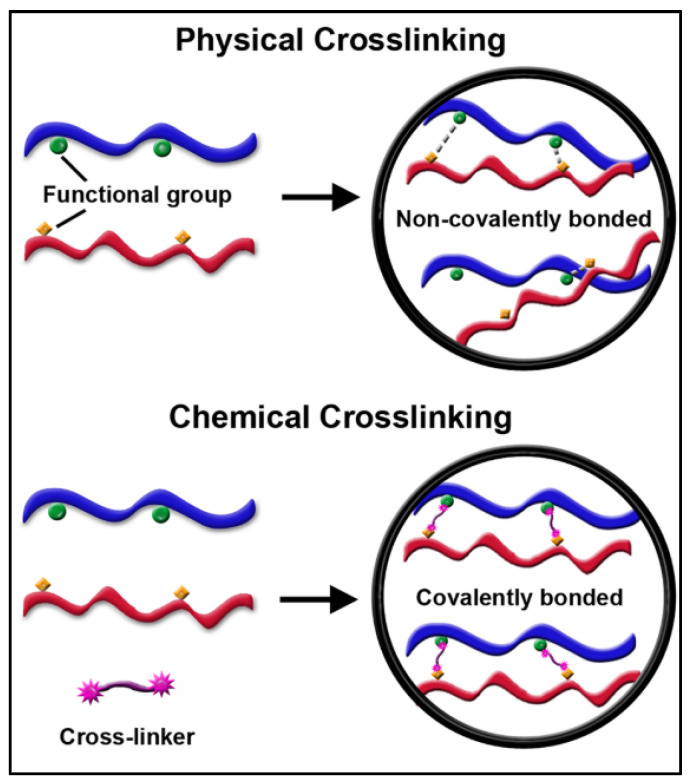
The difference between physical and chemical cross-linking [84].

Chemical cross-linking is a promising method for producing high plasticization-resistant membranes. The difference between physical and chemical cross-linking is illustrated in Figure 8, where covalent bonding is present in chemical cross-linking.

Table 4 below summarizes the polymer-modification improvement strategies using various cross-linking methods that can help to improve membrane properties, enhancing performance and resistance towards plasticization and aging. Grafting is another route of polymer modification that is used to stabilize the polymer against plasticization [85] and improve membrane performance [83]. Grafting can be accomplished using hydroxyl, carboxyl, sulfuric compounds and amines [25], and can be accomplished by the addition or replacement of small functional groups onto the polymer’s rotating unit. This is achieved via attaching the oligomeric chains in an irregular manner to the side chain of the main polymer either via chemical, radiation, photochemical, or plasma-induced techniques. The irradiation method is used to modify the insoluble polymer membrane [83].

##### Incorporation of CO_2_ Philic Groups

Some challenges associated with chemical cross-linking are the creation of ester linkages when diol is used as a cross-linking agent, and the linkages formed can be hydrolysed when a membrane is utilized in the strong acid-gas feed streams. The hydrolysis lessens the membrane separation and plasticization resistance [74]. Hence, a novel cross-linking step has been established which is decarboxylation of the acid pendant at high temperature, which will generate free radical sites that can initiate the cross-linking process without a diol cross-linking agent, and hence will not cause creation of ester [83].

This has been achieved by Zhang et al. [92] by synthesising CADA1 and CADA2, the two carboxylic acid-containing diamines, to polymerize with 6FDA, BTDA and DSDA, respectively. The results showed that the CO_2_-induced plasticization does not happen at CO_2_ pressure up to 30 bar for 6FDA-CADA1-425, which has CO_2_ permeability of 917.4 Barrer and CO_2_/CH_4_ selectivity of 28.11 [3]. Kratochvil and Koros [93] synthesized 6FDA-DAM-DABA (2:1) by quenching from above Tg. It was found that the membrane has a higher CO_2_ plasticization pressure of 34 bar. Quenching PI gives a large free volume to the polymer. The same finding has been observed by Wind et al. [94], where the 6FDA-based copolyimide was applied with covalent and ionic cross-linking with carboxylic acid groups, and showed plasticization resistance up to 55 bar for CO_2_ pure gas, and up to 59 bar for 50:50 CO_2_/CH_4_ mixtures. Numerous advantages and disadvantages are associated with the decarboxylation approaches, such as the potential of triggering the collapse of the transition layers and the substructure established using decarboxylated materials. In order to resolve this issue, thermal cross-linking at a temperature below the Tg has been explored [83]. High-temperature decarboxylation, slightly below the Tg, resulted in more free volume compared to diol or diamine cross-linking agents [3].

Zhang et al. [95] formulated two Tröger’s base (TB)-based polymers by polymerization from two spirobichroman-containing diamine monomers, TB-MSBC. Its gas separation performance is much better, with CO_2_ permeability of about 7.6 times higher than the one with of 6FDA-MSBC. This is largely due to the ineffective chain packing via the addition of the spriobichroman structure and a ladder-type bridged bicyclic TB unit.

Hu et al. [96] enhanced the surface polarity of polyacrylonitrile hollow-fibre-supported polydimethylsiloxane (PDMS) membrane through surface grafting, which improved the surface roughness. The initiator 3-aminopropyltriethoxysilane containing one amine group was combined into PDMS, and polyvinyl pyrrolidone (PVP) with strong polarity was linked to the surface reacting with amine groups. Substantial improvement in surface polarity (more hydrophilic) after the modification can be proved by the sharp decrease from ~98° to ~28° in water contact angle. The membrane immersed in PVP solution for 10 s improved CO_2_/CH_4_ and CO_2_/N_2_ selectivity, while CO_2_ permeance slightly decreased from ~2500 GPU to ~2440 GPU. For the separation of CO_2_/CH_4_ and CO_2_/N_2_ mixed gases, all CO_2_/CH_4_ and CO_2_/N_2_ selectivities were improved after the modification. This showed enhanced stability of the fabricated membrane.

Grafting and cross-linking with vinyltrimethoxysilane (VTMS) to OH groups has been used to modify CA, followed by hydrolysed methoxy groups via condensation on the silane. The permeability of CO_2_ and H_2_S was 139 Barrers and 165 Barrers, respectively, for pure gas, which is much better than that of neat polymer, while selectivity maintains as per the unmodified. The resultant membrane has lower crystallinity, lower Tg and higher flexibility, hence reduced brittleness [85]. This shows the enhanced mechanical strength of the fabricated membrane.

Through functionalization, the biological response of different cells can be manipulated as well as their hydrophilicity or hydrophobicity. Since PSF is a polymer without available functional groups for further chemical modifications, the grafting of functional groups onto aromatic ring-like formyl, sulfonic acid or chloromethyl, is needed for immobilizing other chemical species. For example, chloromethylated PSF (PSFeCl) membranes are introduced with various functional groups such as hydroxyl group (eOH), azide group (eN_3_), amino (eNH_2_), carboxyl (eCOOH), and sulfonic (SO_3_H) groups. These functional groups are introduced onto PSF membranes surface, thus improving membrane hydrophilicity [48]. By doing this, membrane polarity improved, hence resulting in higher membrane permeability.

##### Incorporation of Bulky Groups

Various studies have been conducted to further improve PI limitations to accelerate its commercialisation. According to Chung et al. [97] and Niwa et al. [98], PIs with a hexafluoro substituted carbon (–C(CF_3_)^2^) in the PI backbone leads to more gas selectivity, mainly for CO_2_ relative to CH_4_, than other glassy polymers with similar permeabilities. Structure and property studies showed that limiting chain packing due to the steric hindrance from the CF_3_ groups, which serve as molecular spacers, and chain stiffness in the polymer including the reduced chain mobility can concurrently increase permeability and selectivity in PIs. The bulky –C(CF_3_)^2^ groups in the dianhydride structure of 6FDA-based PIs increase chain stiffness and increase selectivity while inhibiting chain packing, since the bulky group can serve as a molecular spacer, which can increase permeability. Chain stiffness impacts intrasegmental (rotational) mobility, while packing density affects inter-segmental spacing. A trade-off is needed to prevent chain mobility in order to attain higher selectivity by the molecular sieving mechanism, and to increase the free volume to achieve higher permeability [15].

Sixteen polymers have been identified as potential materials, since they are positioned near the 2008 Robeson CO_2_/CH_4_ upper bound. Seven of them are PIs and most are dianhydride and diamine monomers [25]. These bulky diamines help disrupt chain packing and increase free volume, which contributes to enhanced permeability, while the aromatic rings are responsible for chain rigidity for better selectivity [27]. According to Lin and Chung [97], the 6FDA-durene polymer, with four methyl groups, has a CO_2_ permeability close to 700 Barrer at 2 atm and close to 500 Barrer at 10 atm, with CO_2_/CH_4_ selectivity of 5 and 2.8, respectively. Suhaimi et al. [99] reported enhanced separation performance for the 6FDA-durene PI with ZIF-8 particles functionalized with a different types of amine groups. The results disclosed that the membrane containing 0.5 wt% AAPTMS (N-[3-(Dimethoxymethylsilyl)propyl] ethylenediamine) functionalized ZIF-8 in 6FDA- durene polymer matrix displayed an enhanced and highest CO_2_ permeability of 825 Barrer, and CO_2_/CH_4_ ideal selectivity of 26.2 with 0.2024 FFV, compared to the 6FDA-durene without the filler at 510 Barrer, 8.6 selectivity and 0.1981 FFV [99]. Even though these 6FDA-durene hollow-fibre membranes have impressive separation performance, their gas permeation flux deteriorated significantly with time [97], which shows lower aging resistance.

Li et al. [87] attempted the same concept of introducing bulky groups of tetra-o-isopropyl and naphthalene prepared via the polymerization process, using microwaves on three commercial PIs, namely dianhydrides including ODPA, BDPA and 6FDA. The resultant membrane had high FFV values (0.19) with bimodal size distribution of microcavities having average diameters in the range of 8.0 Å–9.0 Å and 5.6 Å–6.3 Å, respectively. The incorporation of these bulky groups successfully enhanced polymeric backbone rigidity while disrupting chain packing, resulting in high gas permeabilities. The PI-3F film had high H_2_ and CO_2_ permeability values of 722 and 849 Barrer, respectively, while upholding similar selectivities for H_2_/CH_4_ and CO_2_/CH_4_ gas pairs with other reported PIs of the same dianhydride, and showed outstanding gas separation performance for the O_2_/N_2_ gas pair which is near the 2008 Robeson upper bound.

Wang et al. [88] explored the outcome of rigid, bulky, and diamond-like structure adamantane grafted onto amidoxime-functionalized PIM-1 (AOPIM-1) mainchain through a simple acyl chloride substitution reaction, to function as a side group that can adjust the chain packing. D-spacing could be finely tuned by adjusting the mole ratio of the grafted adamantane moiety. The resultant membrane CO_2_ permeability was enhanced significantly from 981 Barrer for to 2484 Barrer. The ideal separation factors of CO_2_/N_2_ and CO_2_/CH_4_ gas pairs were reported as 31 and 30, respectively, which largely exceeds the Robeson upper bound. This method is hypothesized to enhance the membrane permeability without affecting the membrane stability and aging due to improved rigidity.

##### Functionalization Using Bromination

Another improvement needed is the Matrimid, which has been studied to be functionalized by brominations. Bromination have a tendency to enhance both permeabilities of N_2_ and CO_2_, with smaller decreases in CO_2_/N_2_ selectivity [20]. Rahmani et al. [49], who synthesized the brominated Matrimid 5218, found that this membrane is much more permeable but less selective than pristine membranes due to higher FFV of the brominated membranes. Their studies are focused on altering the structure of Matrimid membranes using thermal treatment (annealing) or chemical cross-linking, and also on the formation of charge transfer complexes to suppress plasticization.

##### Sulfonation

Sulfonation is another polymer modification method using sulfonic acid (SO_3_H) functional groups to replace hydrogen atoms. This is frequently performed with a reaction using sulfuric acid at high temperatures. Previously, a method used was the post-sulfonation technique, but this leads to lesser mechanical and thermal strength than the original polymer. On top of that, it is tough to obtain a high degree of sulfonation using this way. Sulfonation of monomers before polymerization was proposed to resolve this matter [83].

Xu et al. [100] explored the sulfonation/desulfonation process for CO_2_ permeability enhancement by the thermal treatment in order to harness its plasticization resistance benefit. Gas separation performance, chain rigidity, and interchain distance were analysed with respect to the degree of sulfonation (DS). Sulfonation and desulfonation reaction successfully enhanced the microvoids, promoted interchain hydrogen bonding by the SO_3_H group, and boosted the rigidity, preventing the polymeric membrane from melting during heat treatment. As the DS increased, gas permeability increased by 3.2-fold, reducing CO_2_/CH_4_ selectivity. The performance of this modified membrane for CO_2_/CH_4_ has successfully reached the 2008 Robeson upper bound. Largier et al. [101] evaluated thermal stability, physical properties, and gas transport characteristics of sulfonated poly(phenylene) (sPP) as a function of ion-exchange capacity and counter-ion type. Increasing ion exchange capacity and counter ion complexation strength within sPP led to greater density and reduced FFV. Unsulfonated poly(phenylene) (PP) had a Tg at 391 °C, which shifted to 420 °C upon sulfonation. The increase in Tg shows enhanced resistance to plasticization. PP had high CO_2_ permeability (152 Barrers) with moderate CO_2_/CH_4_ selectivity (12.6). The one with ion exchange has higher CO_2_/CH_4_ selectivity, with lower gas solubility and diffusivity. The tuneable ideal gas permselectivity is largely determined by the sulfonate group concentration and counter-ion type.

##### Lithiation

Guiver et al. [90] applied lithiation chemistry to chemically modify the PSF polymer. By choosing the appropriate selection and site of the functional group, the properties such as solubility, FFV, and chain rigidity were favourably altered. Attachment sites and cross-linking ability of the membrane have been provided by the reactive groups. Functionalisation imparts hydrophilicity, hydrophobicity, reactivity and ionic character of the resultant polymer. The lithiation modification technique harnesses benefits from the sulfone group of PSF that possess strong ortho-directing power. One of the best ortho-directing groups in lithiation chemistry is PSF, due to the availability of a strong electron-withdrawing effect and capability of the lone pairs of electrons on the sulfone oxygen atoms to combine with the lithiating agent. Butyllithium substitutes the ortho-sulfone hydrogen atoms with lithium atoms so that the aromatic carbon atoms to which lithium is attached nominally have a negative charge. The activated site of lithiation is exclusively ortho to the sulfone linkage group. Examples of lithiation are direct lithiation, bromination, carboxylation, sulfinic acid, alcohols, amine, azide cycloadditions, silyl, and tricyclic polymers. During gas separation, the incorporation of basic functional groups undergoing a chemical interaction with acidic CO_2_ feed gas increased the selectivity from 5.5 to 10, reducing permeability from 1.5 to 0.12 Barrer. In the case of aminated PSF, high interchain hydrogen bonding increases the chain stiffness, thus improving its stability.

##### Esterification

Esterification, which involves the reaction between acid and alcohol, is also an example of cross-linking. The cross-linked membranes described in the patent of Koros et al. [91] sustained the membrane transport properties over long-term operation, by adding PI with alcohols, olefins and carboxylic acid or ester groups in its cross-linkable sites to create a limited rotational ability of its chain. The monomer that offers a PI chain with restricted rotational capability is 6FDA, or 4,4′-(hexafluoroisopropylidene) diphtalic anhydride [102]. Nevertheless, the synthesis of modified PIs is much more complex since it comprises several synthesis and purification steps to obtain monomers at sufficiently high purity to produce high-molecular-weight PIs compared to typical PIs [23].

Previous chemical cross-linking methods have a low percentage of cross-linking (<25%), and flexible chains are still available. Crosslinking makes the polymer chains intertwine with each other and make it tighter due to the shrinking effect of cross-linking bridges. The FFV and local mobility are reduced, causing significant deterioration of permeability [58]. Further improvement is needed in this method in order to overcome all these issues.

#### 4.1.3. Thermal Crosslinking and Thermally Rearranged (TR) Polymers

Thermal treatment has been explored by various researchers to improve the membrane performance, enhanced plasticization resistance and improved mechanical strength. Xu et al. [103] fabricated a membrane from phenolphthalein-based cardo poly (arylene ether ketone) (PEK-C) material that successfully enhanced the gas permeability and antiplasticization properties using thermal treatment that promotes interchain cross-linking. The results showed that the lactone rings’ decomposition in cardo moieties and the formation of biphenyl linkages via cross-linking successfully improved performance and plasticization-resistance properties. The enhanced gas permeability is specifically due to the enhanced chain interspace and FFV, while the plasticization resistance is due to the enhanced rigidity. The CO_2_ permeability improved by 110-fold, with a satisfactory CO_2_/CH_4_ selectivity compared to PEK-C neat membrane. Plasticization pressure was significantly improved from 2 to 30 bar maximum testing pressure.

The same findings were found by previous researchers [104,105]. Qiu et al. [104] prepared 6FDA-DAM:DABA(3:2) via the modification of monomers’ proportion. The thermally cross-linked membranes can withstand up to 48 bar for pure CO_2_ gas and 69 bar for 50:50 of CO_2_/CH_4_ mixed-gas separation, showing enhanced plasticization resistance. Chen et al. [105] also concluded that the PI hollow fibres cross-linked at 350 °C under N_2_ atmosphere sustained good binary gas performance under 69 bar for over 45 and 95 h testing. It was determined that thermal cross-linking enhanced membrane separation effectiveness, plasticization resistance, and mechanical strength of fibres.

Deng et al. [106] reported a method to concurrently improve membrane transport properties via thermal oxidation at sub-Tg temperatures. Copolymers containing a component that can enhance Tg and functional groups that decompose and cross-link at lower temperatures are responsible for this improvement. Crosslinking increased polymer FFV, reduced the d-spaces and inhibited CO_2_-induced plasticization. At CO_2_ pressure up to 30 bar and 35 °C, no plasticization occurred. Pure gas CO_2_ permeability of 88.5 Barrer with a CO_2_/CH_4_ ideal selectivity of 38.8 was attained. However, the permeability and ideal selectivity were reduced to 55 Barrer and 29, respectively, when separating equal molar CO_2_ and CH_4_ mixed gas between 10 to 60 bar due to the competitive sorption effect.

Du et al. [107] successfully prepared covalent cross-linked PIMs by a nitrene reaction from diazide precursors, which showed plasticization resistance to the condensable gas up to 20 bar for single-gas CO_2_ and CO_2_/CH_4_ mixtures. It was concluded that covalent cross-linking is better at reducing plasticization [94]. 1,2-ethylene diamine (EDA), a linear aliphatic diamine, is prepared to cross-link with 6FDA-durene, followed by thermal annealing at 250 °C. It showed higher plasticization pressure at 50 bar versus 21 bar for the original 6FDA-durene membrane. The d-space was reduced, and the structure tightening was improved by adding cross-linking agents into polymer chains. The higher the thermal annealing temperatures, the stronger plasticization resistance is achieved by the material. Incorporation of amine bonds further enhanced polymer characteristics such as hydrophilicity and stability in solvents [3].

##### PBOs, PBTs and PBIs

High thermal cyclization temperature, which can cause polymer chain segmental rotation destruction leading to lower chain packing density and enhanced microvoid formation, has been introduced to achieve high gas-separation performance. Aromatic polybenzoxazoles (PBOs), polybenzothiazoles (PBTs), and polybenzimidazoles (PBIs) are glassy polymers with flat, stiff, rigid-rod phenylene heterocyclic ring units [83]. These polymers are insoluble in almost all solvents due to their rigid aromatic structure and intermolecular hydrogen bonding, hence their higher plasticization resistance [39]. Thermal conversion of soluble aromatic PIs containing pendent functional groups at ortho position (for example -OH) to the heterocyclic imide nitrogen in the polymer backbone to aromatic PBOs or PBTs have been described as a promising method to produce PBO or PBT polymer membranes [35].

These polymer membranes showed 10 to 100 times better CO_2_ permeability, and their CO_2_/CH_4_ separation factors were comparable to those of conventional polymer membranes [35]. High-temperature treatments of up to 450 °C lead to brittle structure and the initiation of polymer thermal degradation [39]. Therefore, more optimization needs to be achieved for the future application of this type of polymer [14]. Wu et al. [58] stated that the class of this aromatic polymer, poly (2,6-dimethyl-1,4-phenylene oxide) (PPO) membrane, has different mechanical weaknesses due to its high chain mobility caused by the flexible aromatic ether bond.

##### Template Polymerization

Another method attempted for the modification of polymer during polymerization is template polymerization, invented from natural polymerization processes. The porogen-induced pore formation has been performed via a sacrificial thermal decomposition. This approach is very beneficial in producing membranes with enhanced resistance to plasticization. The methods of template polymerization and use of porogens can be found during the preparation of carbon molecular sieve (CMS) fabricated by heating the PIs to a temperature range of 700–900 °C. The high-temperature heating process results in PIs’ degradation, hence the relatively low-temperature pyrolysis was proposed. The membrane comprises many microvoids, which were caused by “template-like effect”. CO_2_ permeability of the membranes was increased compared to its polymer precursor [83].

##### TR Polymers

**Figure 9:**
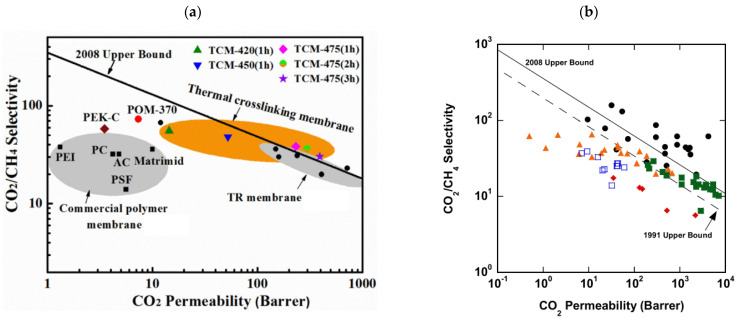
Illustration of thermal cross-linking and TR membranes on the 2008 upper Robeson plot. (**a**) POM-370—(TR PEK-C with oxidative pretreatment; TCM-X(y)—(TR PEK-C with thermal treatment at x °C (y hours), [103] (**b**) CO_2_/CH_4_ separation performance of several polymer materials described in the literature. TR polymers (●), PIMs (■), Perfluoropolymers (◆), Polyimides (▲) Poly(RTIL)s (□), [23].

Alternatively, one more innovation that leads to the achievement of high-performance membranes is that of TR polymers. They have several main advantages, namely good permeability and selectivity, high chemical resistance and higher plasticization resistance [23]. For CO_2_/CH_4_, several TR polymers achieved high performance, close to the 2008 upper-bound limit towards high permeability, as illustrated in Figure 9a,b. The underlying factors for their remarkably high diffusion coefficients are related to the high FFV and high gas solubility [57].

TR polymer also shows high durability and robustness after the removal of the chemical contaminants. Liu et al. [108] examined the effect of toluene on pure and mixed CO_2_ and CH_4_ gas transport properties for a TR polymer formed from a PI precursor based on 3,3′-dihydroxy-4,4′-diamino-biphenyl (HAB) and 2,2′-bis-(3,4-dicarboxy-phenyl) hexafluoropropane dianhydride (6FDA). As the polymer is tested using CO_2_, CH_4_, and their equimolar mixture with 0.3 toluene activity, CO_2_ and CH_4_ permeability are reduced by >90% due to antiplasticization and competition effect. CO_2_/CH_4_ selectivity was at maximum as toluene activity increased, reflecting the interaction between competitive sorption, antiplasticization, and plasticization. CO_2_/CH_4_ separation factor values in the ternary mixture initially increased from 23.4 to 25.3 at low toluene activities, and at higher toluene activities it reduced to 20.3. The gas permeability was recovered when toluene was removed from the feed gas mixture with 84% of CO_2_ and CH_4_ permeability coefficients regained from their original number and maintained at that value throughout six days of the experiment duration. This indicated high durability to the swelling, with just 16% permanent changes to the polymer. The reduction in permeability value was mainly due to antiplasticization, competitive sorption, and pore-blocking of the bigger toluene molecules, while having the side advantage of improved selectivity.

Scholes et al. [109] developed two TR membranes from the precursor co-PI 4,40-(hexafluoroisopropylidene) diphthalic anhydride (6FDA), 3,30-dihydroxyl-4,40-diaminobiphenyl (HAB), and 2,4,6-trimethyl-m-phylenediamine (DAM) (6FDA-HAB-DAM), where one of the membranes undergoes cross-linking with 3,5-diaminobenzoic acid (DABA) during TR. The results showed that both have high CO_2_ permeability of 196 and 218, respectively with CO_2_/N_2_ selectivity of 20.0 and 20.5. The permeability of H_2_S in the TR membrane was constant as a function of H_2_S feed partial pressure and increased with temperature. There is no evidence of H_2_S plasticization in this TR membrane, supporting the chemical resilience of the TR polymer. The increase in H_2_S permeability with temperature for non-cross-linked PI is indicative of a diffusivity control process, as diffusivity theoretically is known to increase with temperature. For the cross-linked membrane, the H_2_S permeability decreased with temperature due to the solubility-dominant resultant from the cross-link, that alters the FFV and bimodal cavity distribution.

Lee et al. [110] highlighted that chain rigidity of cross-linked-polybenzoxazole TR (XTR) could be enhanced during an isotherm treatment at its Tg. Densification reduced the unwanted pinhole defects (pore diameter < 5 nm) on precursor fibres and they were completely healed after thermal treatment (>400 °C). The XTR hollow fibres exhibited CO_2_ permeance of ~2300 GPU and a CO_2_/N_2_ selectivity of 17.4, with a skin thickness of 103 nm.

Ricci et al. [67] studied the binary CO_2_/CH_4_ sorption in HAB-6FDA PI (3,3′-dihydroxy-4,4′-diamino-biphenyl (HAB) 2,2′-bis-(3,4-dicarboxyphenyl) hexafluoropropane dianhydride (6FDA) and its TR450 at 35 °C and ~30 mol% CO_2_ mixture composition. The competition effect enhanced the CO_2_/CH_4_ solubility selectivity of CO_2_ while reducing the CH_4_ solubility. CO_2_ has a harmful consequence on diffusivity selectivity since it can dilate the polymer and enhance the diffusion of CH_4;_ thus, separation will then only be controlled by solubility selectivity. With higher fugacity, solubility selectivity becomes dominant over diffusivity selectivity. TR450 has much higher value of solubility selectivity relative to the precursor PI, which has a positive impact on the CO_2_/CH_4_ separation. This is due to the higher free volume available for TR450, making competitive sorption effect less significant, resulting in CO_2_ diffusion coefficients that are nearly similar in pure and multicomponent conditions. It has been hypothesized that solubility selectivity of mixed-gas-enhanced relative to pure-gas solubility selectivity, probably due to solubility of CO_2_ under mixed-gas conditions, is higher during the competitive sorption compared to CH_4_ in terms of to their condensability values. Mixed-gas diffusivity selectivity, in this case, is always lesser than pure-gas diffusivity selectivity, and this suggests that membrane material with high solubility selectivity is preferred for multicomponent CO_2_/CH_4_ separation, to reduce the plasticization impact and maintain high-performance stability.

**Figure 10:**
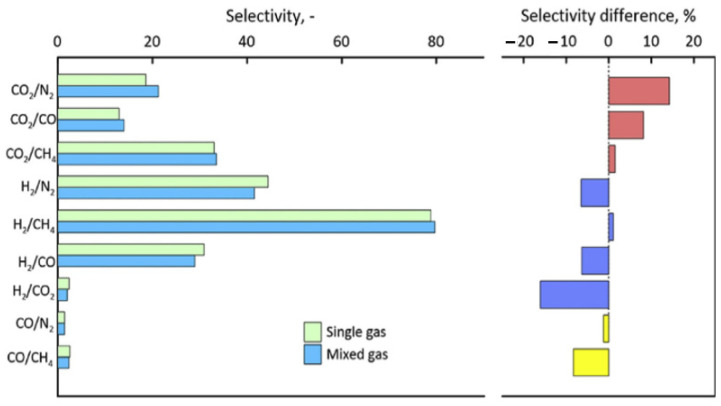
Performance comparison for TR-PBOI single- and mixed-gases hollow-fibre membrane [38].

Brunetti et al. [38] investigated the mutual influence of binary gas mixtures through TR poly(benzoxazole-co-imide) (TR-PBOI) membranes. They made the same conclusion, in which TR polymer mixed-gas selectivity is greater than single gases for CO_2_/CH_4_ separation, with slightly different selectivity, and this is illustrated in Figure 10 for various gas combinations. In addition, they [111] also evaluated the aging trend of a TR-PBOI MMM (Mixed Matrix Membrane) loaded with 0.5 wt.% of oxidized multiwall carbon nanotubes (MWCNT). After 166 days, the permeability was reduced for both membranes of TR-PBOI and TR-PBOI MMM, with MMM reduction being slightly higher. TR membrane reduction was ~10.3% for CO_2_ and ~24.4% for N_2_, whereas the MMM has a reduction of 13% for CO_2_ and 25.5% for N_2_. Overall, the incorporation of CNTs in the TR polymer membrane enhanced the separation performance, with a 17.1% increase in CO_2_ permeability and a 16.5% improvement in CO_2_/N_2_ selectivity. In summary, although the MMM was thinner and more permeable, and supposed to be more vulnerable to physical aging than the pure TR membrane, no significant differences were observed in their aging trend [111]. Further studies are needed to evaluate the effects of precursor structure and explore the effects of physical aging on TR polymer thin films [23]. Li et al. [3] reported that TR polymer membranes do not show reduced selectivity at CO_2_ fugacity (~15 bar) and show excellent resistance to plasticization at CO_2_ partial pressure up to 20 bar.

**Figure 11:**
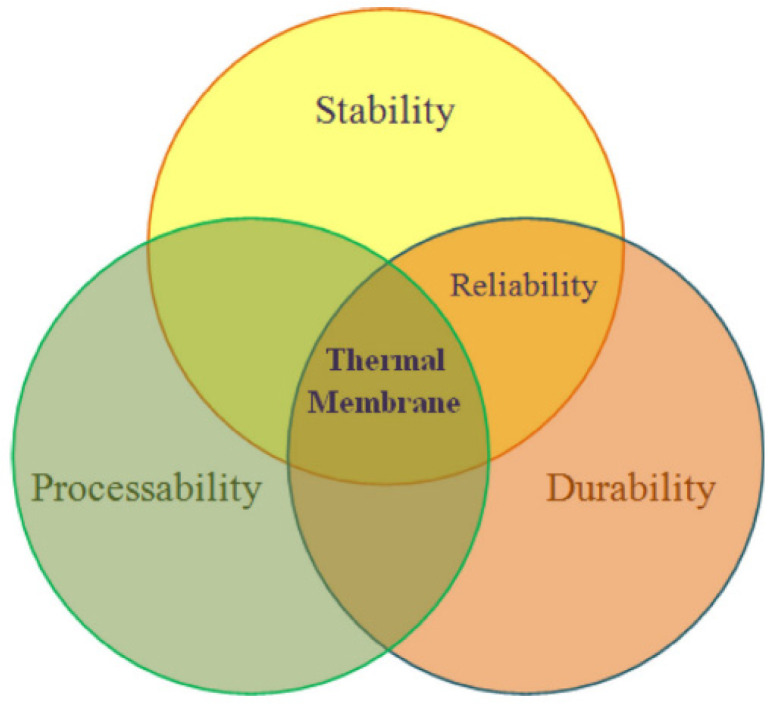
The target to develop thermally stable polymers [81].

The Young’s modulus and elongation break of TR450-30min are 95% and 490%, respectively, which is lower than those of CA, although they have good separation performance. This result suggests that a trade-off exists between gas transport and mechanical properties [39]. Gas separation effectiveness of the polymer membrane can be custom-made by the use of suitable thermally induced structure transformation and polymer chain rearrangement [83]. The aim to develop thermally stable polymers for advanced high-performance gas-separation membranes is illustrated in Figure 11, where all factors, namely stability, reliability, and durability, need to be achieved at a similar time while maintaining good processability [81].

Membranes with lesser FFV, higher resistance to CO_2_-induced plasticization phenomenon, better packing density, and higher Tg can be obtained via thermal annealing. However, the improved plasticization resistance resultant from chain rigidity is accompanied by the decline in membrane permeability [83], as a consequence of thin skin densification during the annealing process. Rapid quenching has been introduced into the membrane cross-linking step [83], as has innovation of the TR polymer that has managed to improve FFV, solubility and chemical resilience in order to resolve the thermal annealing-associated issue. However, the TR membrane’s mechanical strength needs to be further improved.

#### 4.1.4. Ultraviolet (UV) Radiation Cross-Linking

Both thermal and UV irradiation-induced photo-oxidation of PIMs seem to be effective techniques for reducing aging and plasticization effects via densification and elimination of microvoids and improving membrane performance and mechanical strength. It has been found that the plasticization resistance significantly relies on the number of cross-linkable moieties and the annealing temperature. UBE marketed aromatic PI membranes with maximum operating conditions of 100 °C and 150 bar, respectively [25]. Li et al. [112] investigated the UV-rearranged polymers of PIM-1 and found that single-gas CO_2_ permeance dropped from 6601 Barrer for the original PIM-1 to 4560 Barrer after 10 min of UV radiation, and to 62 Barrer after 4 h of UV radiation. On the other hand, the selectivity enhances slightly from 15.3 for the original, 16.1 after 10 min, and 23.7 after 4 h. This is because the FFV for CO_2_ has been reduced from 0.222 to 0.117. Mixed-gas separation of equimolar CO_2_ and CH_4_ for PIM-UV 20 min results in CO_2_ permeability reduction from its single-gas value by just 17%, and for PIM-UV 30 min by only 3%. As for the selectivity, PIM-UV 20 min drops by 16% while PIM-UV 30 min drops by 6% only, which shows great stability. The PIM-1 backbone undergoes homolytic cleavage at the C-H bond and an intramolecular 1,2-migration reaction to form the UV-rearranged PIM-1 under the photochemical reactions. The resulting UV-rearranged PIM-1 destroyed the kinked feature of the original PIM-1, hence leading to the significant reduction in pore size and FFV.

Contradictory findings have been observed by Altun et al. [113], who fabricated membranes comprising of a semi-interpenetrating network of PSF and cross-linked polyacrylate. They were manufactured via nonsolvent-induced phase inversion and then treated using UV. Tetrahydrofuran (THF) or 1,4-dioxane (DIO) were used as a cosolvent to the N,N-dimethylformamide (DMF)-based polymer solutions, and cast films were evaporated before coagulation. A post-treatment was conducted to enhance the flux by dipping the UV-cured PSU-based films in DMF for 48 h. The resultant membranes exhibited higher permeance with declined selectivity, making them suitable for the preparation of support for thin-film composite (TFC) membranes. Longer evaporation times and lower initial cosolvent concentrations have reduced or eliminated the macrovoids formation. Kusworo et al. [114] improved the permeability and selectivity of the nanohybrid polyethersulfone (PES) membranes comprising nano TiO_2_ of 0.5, 1, and 1.5 wt%, respectively. It was exposed to UV rays followed by soaking in the ethanol–acetone mixture, and then underwent thermal treatment for 20 s at 180 °C. Testing results confirmed that the nanohybrid PES-nano TiO_2_ with 0.5 wt% nanoparticle loading showed the best performance, with CO_2_ permeability and CO_2_/CH_4_ selectivity of 681 GPU and 43, respectively. The membrane achieved 43% improvement for permeability and 67% improvement for selectivity, compared to the neat membrane.

Parl et al. [115] prepared a series of CO_2_-philic terpolymers by free radical polymerization using three monomers, poly (ethylene glycol) methyl ether methacrylate (PEGMA), methyl methacrylate (MMA), and 4-hydro-xybenzophenone (BPMA), to develop CO_2_/N_2_ separation membranes. In order to produce cross-linked membranes (X-PMB), poly (PEGMA-co-MMA-co-BPMA) (PMB) was cast into thin precursor films, which were then cross-linked by a UV-induced photochemical reaction between benzophenone and the alkyl group. The gas permeabilities of the X-PMB membranes were influenced by the PEGMA content, since the presence of polar ether groups of PEGMA leads to increased CO_2_ solubility and diffusivity. The CO_2_ permeability was increased from 50 to 111 Barrer when the PEGMA content was increased. The permeabilities of N_2_ also increased from 1 to 2.2 Barrer, resulting in a CO_2_/N_2_ value of 50. Compared with PEBAX, this membrane performance is similar or better, demonstrating that the X-PMB could be potentially applied to CO_2_ separation. These studies indicated that UV cross-linking could improve membrane performance and plasticization and aging resistance via densification.

#### 4.1.5. Surface Modification

The relationship between physicomechanical properties of membranes and their surface structure is studied to understand the impact on the membrane performance. Microscopy techniques such as atomic force microscopy (AFM) and scanning electron microscopy (SEM) are employed to study the morphological and structural properties at the micro- and nano-scale [116,117]. Chemical and physical surface modification and functionalization can be performed to improve the membrane stability and performance [118].

Sazanova et al. [116] used chitosan (CS) graft and block copolymers with polyacrylonitrile (PAN) and polystyrene (PSt) modified with ionic liquids (ILs) based on a cation of 1-butyl-3-methylimidazolium ([bmim]) with various anions ([BF4], [PF6] and [Tf2N]) to study the relationship between surface structure and mechanical properties. It was found that the immobilization of ILs resulted in a reduction in the surface packing density of the copolymers and caused deterioration in mechanical properties. Block copolymers were more resilient to this effect than the grafted ones. Nevertheless, the CS copolymers with PAN showed better strength and elastic properties than the ones with PSt [116]. Sazanova et al. [117] also examined the effects of surface roughness in terms of surface free energy (polar and dispersive components) on the nonporous polymeric membranes’ performance and mechanical strength. It was proven that the surface roughness of the polymer membranes affected both energy components, which also depend on the chemical nature of the polymer. The dispersive energy component is assumed to be inversely correlated with any gases’ total permeability. Contrarily, the polar component is inversely correlated with the permeability by gases with the ability for site-specific interactions. Polymers mechanical properties are also influenced by the dispersive component of the surface energy.

The novel imidazolium salts based on bis(2-ethylhexyl) sulfosuccinate anion have been developed as ILs, and the transport of CO_2_, H_2_S, CH_4_ and N_2_ in a series of supported ionic liquid membranes (SILMs) with immobilized conventional (bmim[PF6], bmim[BF4], bmim[Tf2N]) and novel ILs was investigated. The SILM containing 1-butyl-3-methylimidazolium bis(2-ethylhexyl) sulfosuccinate (bmim[doc]) yielded a very high H_2_S solubility and H_2_S/N_2_ selectivity equal to 65. However, the permeability of acid gases through such a membrane had relatively low values, varying in a range of 100–200 Barrer, whereas permeability for SILMs impregnated by conventional ILs (bmim[BF4]) achieved 565 Barrer, mainly due to the solubility component of permeability. The polymeric support surface properties such as free surface energy, surface topology and roughness parameters, were evaluated to estimate the stability of SILMs. Among the tested ILs, bmim[BF4] immobilized in porous polymeric support was more resistant to losses and determined the higher stability of membranes, which correlates with experimental contact angle measurements.

From these studies, it can be deduced that surface modification and functionalization have significant impacts on the membrane performance, stability and mechanical properties. The surface packing density, surface roughness, surface free energy, and surface topology have been evaluated. More studies and characterisations are needed using various types of membranes to gain a deeper understanding on the impact of these properties on the overall membrane performance and stability.

### 4.2. Polymer Blending

Another method that can be considered for membrane performance improvement and plasticization resistance is by using polymer blending, in which the two polymers are not covalently bonded. Aging can also be reduced by polymer blends, but it is usually accompanied with a decrease in the transport properties [72]. The polymer blend can be either homogeneous or heterogeneous. Homogeneous blends have strong interaction between the two polymer phases since both can completely dissolve, forming one solution. This is usually due to their solubility parameters being close to each other, indicating good mutual solubility [59]. A heterogeneous blend means that the phases are still separated into two phases, and this type of blend is usually not preferred due to mechanical strength weaknesses. Compatibilizers can be added to assist the mixing and stabilize the phases interface between the two polymers [46]. For example, Dioctyl phthalate (DOP) can be added to dissolve PVC and CPE. At first, it is utilized to form PVC-DOP and CPE-DOP, where each blend has its own Tg. At a particular amount of DOP, homogeneous ternary phase is formed. This happens when the presence of DOP can sufficiently counterbalance the repulsion forces between the two polymers, which is not immiscible initially. This behaviour is common where the ternary blends contain a third component that is miscible with the two immiscible polymers, which in this case is the DOP. Surpassing the critical amount makes the binary phases disintegrate, and there is reduction in the two phases’ interfacial tension, forming a miscible ternary blend. Properties of polymer blends such as compatibility, miscibility and others, are affected by the presence of plasticizers which help the two polymers to be miscible [59].

Mazinani et al. [76], showed that blending Matrimid and high-performance polyamide-imide (PAI) leads to higher selectivity with a slight reduction in permeability. This is due to the strong intermolecular interactions, resulting in polymer chain packing enhancement and the specific interaction between CO_2_ and the polymer matrix, which promotes CO_2_ solubility and increases the selectivity. Additionally, the blended system has significantly improved the plasticization resistance at pressures up to 20 bar. The PES/PC blend produced good membrane properties while having similar mechanical strength as PES on its own, and the amorphous PC does not affect the blended polymer chemical resistance. In addition, the PES/PA blend also displays enhanced toughness with good morphology. Polyamides that are semicrystalline have resulted in a higher chemical resistance of the blended material than the pure PES [119]. Mannan et al. [120] studied PSF/PES blending and found that the ideal selectivity improved by 65% with improved thermal stability. However, there is gradual reduction in permeability as the weight fraction of PES in the blend increased, but the plasticization pressure of PSF/PES blend membranes was successfully increased from 20–25 bar to 35–40 bar via this blending.

Blending hard glassy polymer with soft rubbery polymer results in a membrane with high separation performance. A study by Mosleh et al. [121] indicated that a blend membrane with 10 wt.% PES or Matrimid with Pebax^®^ managed to increase selectivity up to ~56.6 compared to the neat Pebax^®^, which is a 67.5% increment, with permeability reduction to 21.7, which is a 14.7% decline from the neat. Polymer blending of fast-aging polymers with the slower aging polymers is another effective method for producing an enhanced aging-resistance membrane. Blending can also be performed on polymers with high and low plasticization resistance to average the effects [50]. In addition, composite membranes comprising copolymers that have both glassy and rubbery segments have also been explored. The mechanical support comes from the glassy segments that form the structural frame, while the rubbery part forms the continuous microdomains in the membrane. Its flexible structure enhances gas transport, resulting in permeability enhancement compared with the glassy polymer [49]. The flexible, rubbery polyethyleneoxide polymers usually have low densities, resulting in an overall increase in FFV of the membrane when blended with high-density, low-permeability glassy polymers. Nevertheless, the increase in FFV leads to enhanced diffusion of all species, which is accompanied by a loss in diffusion selectivity and a loss in the overall selectivity [74]. More polymer blending studies need to be explored in order to achieve breakthrough performance and stability. This type of improvement is easier and manageable for quick commercialization.

### 4.3. Facilitated Transport Membranes (FTMs)

FTMs depend on a chemical reaction between the gas and a carrier in the polymeric membrane. For CO_2_-selective FTMs, the active carrier is normally basic, and will react with acidic gases to increase CO_2_ loading [39]. The reacted species flow across the membrane while the unreacted gases are suppressed, and the partial pressure difference across the membrane acts as the driving force for the transport. The carrier enhanced the permeability and selectivity through increased loading in the membrane via a solution–diffusion mechanism similar to polymeric membranes [10,49]. Faster diffusion of gases in the liquids rather than the solids results in this process having higher permeabilities [4]. Amines reacted reversibly with CO_2_ according to a zwitterion mechanism [39]. The mobile carrier can actively interact with CO_2_ and achieve high selectivity, while high permeability is gained via the loose flexible polymer chain [25].

Kojabad et al. [122] explored a new type of FTM in which aniline molecules were incorporated into the poly (ether-block-amide) (Pebax) matrix as the semimobile carrier. Aniline molecules play a vital role in removing the trade-off limit, so that in aniline-containing membranes, permeability and selectivity were promoted concurrently. Based on the molecular simulation, aniline molecules had a high diffusion coefficient and could move freely between polymer chains and ease the transport of CO_2_ molecules through two hopping and vehicle mechanisms. It was found that hydrogen bonding in the structure of the aniline-containing membranes lead to the improvement of Young’s modulus in these membranes. The CO_2_ diffusivity was improved 7.5-fold compared to the neat membrane. Furthermore, 50 wt% of aniline has shown a permeability value of 151 Barrer and a selectivity of 92.5, which increased by 76% and 101%, respectively, compared to the neat Pebax membrane.

Tu et al. [123] introduced the salting-out effect as a suitable method for improving CO_2_ separation membranes by hindering the permeation of N_2_ and CH_4_. They synthesized fluorion-based protic ionic liquids (FPILs) and FTM of CO_2_, and found the salting-out effect on N_2_ and CH_4_ in FPIL solutions. High CO_2_ permeability, CO_2_/CH_4,_ and CO_2_/N_2_ selectivity up to 2572 Barrers, 774 and 387, respectively, were obtained at 40 °C and 0.1 bar CO_2_ partial pressure in [DMAPAH][F] solution of 40 wt% water. Kunalan et al. [124] enhanced this further, by developing polymer–dendrimer composite membranes consisting of poly(vinyl alcohol) (PVA)–poly (ethylene glycol) (PEG), cross-linked with glutaraldehyde (GA) membranes, and further incorporated with amine-rich poly(ether-imine) (PETIM) dendrimer generations 1–3. Compared with known FTM systems, the discovery of the third-generation dendrimer-based composite membrane shows the most optimal CO_2_ permeance and CO_2_/N_2_ selectivity. The molecular simulation revealed the details of the affinity of CO_2_ to the membrane among dendrimer generations. CO_2_ permeance and CO_2_/N_2_ gas pair selectivity of 208 GPU and 56, respectively, were achieved, at a feed pressure of 2 bar and 30 °C. The membrane possesses long-term stability for 200 h.

**Figure 12:**
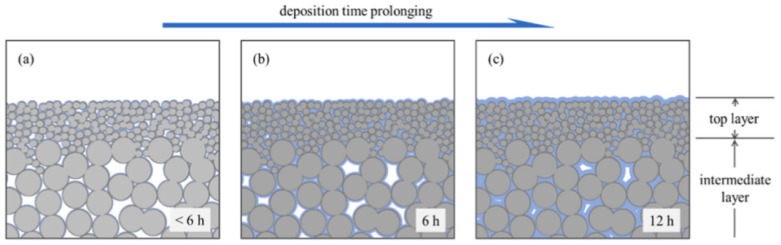
Ionic liquid filled the layer with deposition various times (**a**) on pore wall (**b**) top layer (**c**) top and intermediate layer [125].

Supported ionic liquid membrane (SILM) using low surface-tension ionic liquid [Emim] [Tf2N] was reported by Sun et al. [125]. The membrane was prepared with the supercritical fluid deposition (SCFD) method and supercritical carbon dioxide (scCO_2_). The IL shuttles and diffuses into the interior of the support to render the deposition in γ-Al_2_O_3_ nano–micro pores. Under optimum conditions with deposition time of <6 h, IL filled the top layer of ceramic support only, as illustrated in Figure 12. The [Emim] [Tf2N]-SILMs exhibited CO_2_ permeance of 22.6 GPU and CO_2_/N_2_ selectivity of 27.1, while maintaining proper pressure resistance and long-term stability with no significant IL loss within 20 days. Free-standing PIL/IL composite membranes of 1-bromohexyl-1 methylpiperidinium bromide (Br-6-MPRD) grafted poly(2,6 dimethyl 1,4 phenylene oxide) (ILPPO) incorporated with different amounts of free Br-6-MPRD (0, 2, 5 and 10 wt%) has been explored by Vijayalekshmi et al. [126]. Gas permeation at 1 bar pressure and 25 ºC revealed the best CO_2_ permeability and CO_2_/N_2_ permselectivities which are 907.20 Barrer and 12.94, respectively for ILPPO/Br-6-MPRD-2. A novel FTM is proposed by Zhang et al. [127] for the transfer of CO_2_ from carbene to phenolated anion. They investigated a series of imidazolium-based phenolate ILs that have dual-site interaction centres to segregate CO_2_ from N_2_ by SILMs. High permeability and selectivity of CO_2_/N_2_ are achieved, which is 2540 Barrers and 127, respectively, in 1-butyl-3-methylimidazolium phenolate ([bmim][PhO]), containing 15 wt% H_2_O under humidified conditions. They [128] further introduced four diamine-monocarboxylate-based protic ionic liquids (PILs) to prepare SILMs. Humidified conditions with 15 wt% H_2_O result in higher CO_2_ permeabilities than the dry condition. The highest CO_2_/N_2_ and CO_2_/CH_4_ selectivities are 151 and 72, respectively. Zhang and his team [129] expanded their study for the separation of H_2_S/CH_4_ and H_2_S/CO_2_ in SILMs for the co-removal of H_2_S and CO_2_ from natural gas. The highest ideal selectivity of H_2_S/CH_4_ and H_2_S/CO_2_ are 142 and 12, respectively, with H_2_S permeability of 5279. It was found that neutral ILs (1-butyl-3-methylimidazolium tetra-fluoroborate and 1-butyl-3-methylimidazolium trifluoromethanesulfonate) show high permeability of CO_2_ and H_2_S, and competitive permselectivity of CO_2_/CH_4_ and H_2_S/CH_4_.

To be on par with the current latest advancement, Halder et al. [130] investigated the high free-volume polymer of PIM-1 blending with ionic liquid (IL) [C6mim][Tf2N] performance. The blend membrane shows a slightly lower permeability and enhanced selectivity. Nevertheless, PIM-1 and the IL are not compatible, hence the polarity of PIM-1 had to be improved using poly(ethylene glycol) (PEG) as a compatibilizer by blending and copolymerization with a PEG containing anthracene maleimide comonomer (CO). Better polymer-IL compatibility was achieved with copolymerization (PIM-COP) in the IL concentration range 2.5–10 wt% compared to the PIM-PEG blend IL, which leading to enhancement of CO_2_/N_2_ selectivity from 19 to 30 at 30 °C and above 800 Barrer CO_2_ permeability [4].

A review of the recent development of FTMs with ionic liquids for CO_2_ separation has been conducted by Klemm et al. [131], deliberating the challenges and opportunities in its application. Traditional supported liquid membranes that use volatile solvents have an issue with stability under pressurized conditions or vacuum, and tend to dry out, which affects the performance. Galizia et al. [39] provided a comprehensive overview of FTM covering various applications of CO_2_, olefin–paraffin, and O_2_/air separation.

**Figure 13:**
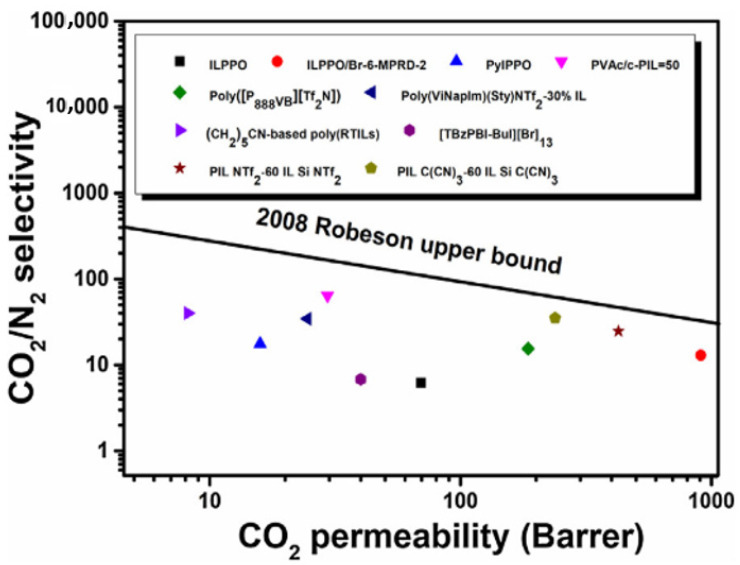
Reported literatures for FTMs vs. 2008 Robeson upper bound [126].

SILMs are most efficient at low pressures, and their CO_2_/CH_4_ selectivity decreases intensely with the increasing total feed pressure for a binary gas mixture [10]. On top of that, many of these room-temperature ionic liquid, RTIL-based polymers show permeabilities much lower than that of TR polymers or PIMs with similar selectivities [23]. This immobilized liquid membrane has inadequate tolerance to cope with the large transmembrane pressure differences required to achieve sufficiently large gas fluxes [20]. In addition, the immobilized liquid layer is generally water-based, hence water evaporation and hydrocarbon fouling are among the major issues that need to be resolved [14]. Evaporation of contained molecular solvents may result in poor stability of the supported liquid membranes [123]. Despite that, the application of ILs in membranes for gas separation is relatively new, and more studies need to be performed in this area. However, it can be seen from Figure 13 that FTMs performance is close to the 2008 Robeson upper bound, which shows the great potential of this type of membrane for future application with further improvements that need to be made. Table 5 summarizes the most recent FTMs performance comparison with high permeability and moderate selectivity performance. 

### 4.4. Mixed-Matrix Membranes (MMMs)

Because polymeric membranes provide incomplete separation and often have chemical or thermal stability properties that are not sufficient for desired applications, the focus of much of the active research in the field is related to the development of materials that can achieve higher selectivity as well as permeability with more resistant and stabile performance for the intended application [62], such as MMM. Membranes comprising intercalated filler had reduced plasticizer uptake by about 50%, hence lower plasticizer concentration than other samples studied. This shows the limited plasticizer movement via physical hindrances and the impact of plasticizer spreading in the matrix [59].

The performance of current polymeric membranes has reached a level of plateau, and it is difficult to achieve significant improvement without novel innovation [31]. MMM which have been created by the inclusion of nanoparticles as discrete phase (i.e., materials with a pore size < 2 nm) either nonporous or microporous nanomaterials (zeolites, silica, clays, zirconia, alumina, CMS, CNTs, MOFs, POSS, GO, etc.) and metal oxides such as MgO, ZnO and TiO_2_ in the dispersed continuous polymer matrix make up the bulk of the research being conducted nowadays. The high permeability and selectivity of inorganic membranes with the ease of processing of polymeric membranes are combined to improve separation performance cooperatively. The rigid, adsorptive, porous-type inorganic phase offers superior separation properties, and the polymeric phase permits the ideal membrane formation. This solves the problem of fragile inorganic membranes and lower performance in dense polymeric membranes. In addition, MMM demonstrates higher mechanical strength than the corresponding pure polymer and has lower cost than inorganic membranes [20].

MMMs can be considered as membranes comprising solid particles with pores that help to improve the selectivity, and membranes consist of nonporous nanoparticles, which enhance the permeability by increasing the FFV of the membrane [4]. Numerous reviews on MMM can be found elsewhere [50,71,132,133].

Table 6 summarizes some of the latest findings from recent studies. Generally, there are no commercial applications of this type of membrane, since some fundamental issues still need to be resolved, especially the sealing, homogeneity, and agglomeration. Nevertheless, they offer great prospects for many applications in the future.

### 4.5. New Polymer

Research and development in materials focusses on two major areas, which are alteration of current conventional polymers and invention of novel new polymers [83].

#### 4.5.1. High Free-Volume Membrane/Super Glassy Polymer

Free volume can be categorized into a continuous portion that results from oscillations that increase slightly as the temperature is raised, and a discontinuous portion or holes, which increases greatly with increasing temperature. Highly tactic polymers (regular polymer with one species of the configurational repeating unit in a single sequential arrangement) have more free volume than atactic polymers (formed by free radical mechanisms) as a result of special chain conformation. Near the Tg, the tortuosity effect may become less significant than free-volume size and chain mobility [59].

The FFV in glassy amorphous polymer increases with increasing Tg, where the formation of microvoids have been promoted, hence lowering the activation energy [26]. From the past literature, it was hypothesized that free volume can be increased by increasing temperature or decreasing molecular weight either by using low-molecular-weight polymer or the addition of additives such as plasticizers. Free volume also can be enhanced by increasing chain mobility by having more end groups, lowering interaction between chains by having low polarity and decreasing hydrogen bonding, and by increasing hydrodynamic volume via numerous long side chains. Incorporating plasticizer molecules into the polymer can reduce the polymer Tg, particularly using small plasticizer molecules that can significantly enhance the polymer free volume. Therefore, free-volume theory mentions the effect of this lowering of the Tg with the existence of plasticizer [59]. It has also been found that permeability is strongly associated with free volume. The higher the FFV in a polymer, the higher the gas diffusion coefficients [40]. This is aligned with the finding from Bos et al. [64], who also reported the relationship between permeability of polymer membrane and free volume. Matrimid has the highest FFV (0.225), and BPZ-PC has the lowest FFV (0.138), which generally follows their permeability trending.

##### PIMs

High free-volume gas-separation membranes such as poly 1-trimethylsilyl-1-propyne (PTMSP), tetrafluoroethylene/perfluoro-2,2-dimethyl-1,3-dioxole (TFE/PDD) copolymer, PIM-1, TR, and perfluorinated polymers, have been invented to increase permeability factor, where almost one-third of the polymers are unoccupied spaces [21].

The microporosity of PIM polymers is referred to as “intrinsic” because it is formed from their structure instead from a thermal or processing procedure. Gas permeability in PIM-1 is 100 times higher than conventional glassy polymers for gas separation, with negligible effects on the selectivity. The high gas permeability exhibited by PIM is basically due to high solubility coefficients with moderate diffusion coefficients [39]. The extraordinary gas-separation performance of PIMs (refer to Figure 2 for its molecular structure), contributed to the redefinition of Robeson’s upper bounds in 2008 and 2015. The new 2015 upper bounds for the O_2_/N_2_, H_2_/N_2,_ and H_2_/CH_4_ gas pairs were significantly shifted from the 2008 upper bounds, demonstrating the significant influence of highly rigid, molecular sieving PIMs and PIM-PIs derived from triptycene components [21,138].

The PIM-PI-based membrane displayed two orders of magnitude higher permeability than conventional PI such as Matrimid, and average selectivity for CO_2_/CH_4_, O_2_/N_2_, CO_2_/N_2,_ and H_2_/N_2_. PIM-PI is another promising high-performance, porous, organic polymer membrane material for future gas-separation applications [21,138]. However, it does not have a Tg below its decomposition temperature, leading to a higher physical aging rate (PIMs Tg ~442 °C). After 1400 days of operation, thick films based on PIM-1 lose 70% of O_2_ permeability with an improvement in O_2_/N_2_ selectivity of 40%. Chemically modified PIMs lose 50% of their O_2_ permeability in 300 days, with a gain in O_2_/N_2_ selectivity of 30%. Nevertheless, the overall performance of aged membranes in O_2_/N_2_ separation still lies on the O_2_/N_2_ selectivity of about 30% of the 2008 upper bound. A major problem with PIMs is dimensional stability and their overall performance is lower than that of TR polymers [39]. Lately, it has been stated that incorporating polymeric fillers can lessen aging while enhancing selectivity [35]. The membrane loses about 70% of its initial CO_2_ permeability (6576 Barrer) in 192 days for high FFV polymers such as PIM-1 [58].

**Figure 14:**
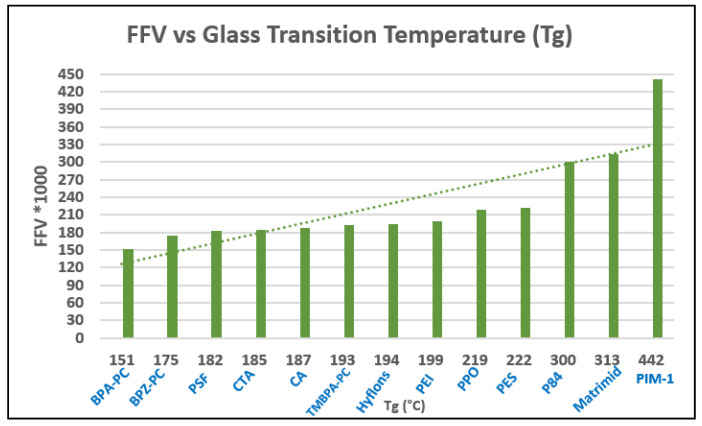
Relationship between FFV and Tg.

The aging rate correlates strongly to the Tg of the polymers. The higher the Tg, the higher the FFV (refer to Figure 14) and the faster the aging rate [16,139]. In the kinetic theory, Tg is not a thermodynamic variable, but it is related to the rate at which the polymer reaches its equilibrium. Thus, Tg is the temperature at which the time scale of the experiment i.e., rate of cooling, is equal to the relaxation time of the polymer chain [140].

**Figure 15:**
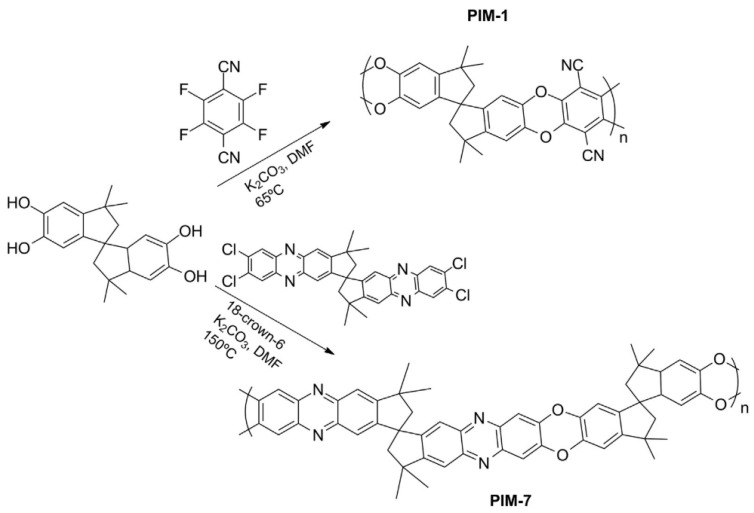
Synthesis and chemical structures of PIM-1 and PIM-7 [23].

PIM-1 and PIM-7 were the only two PIMs initially reported to form films enough for membrane fabrication and testing, since low molecular weights and low yields have been a problem in the synthesis of PIMs. It is difficult to form the film, and it lacks in mechanical strength. In addition, it has lower selectivities as compared to TR polymers. Two key features of PIMs are their kinked backbone (spiro-type structure) and highly restricted backbone rotational movements. The synthesis of the most studied PIM-1 and PIM-7 materials and a molecular model of PIM-1 are shown in Figure 15 [23].

PIMs can be solution-casted directly into their final form without high-temperature heat treatment. The CO_2_/CH_4_ and CO_2_/N_2_ mixed-gas selectivities do not reduce the tetrazole-containing PIMs (TZPIMs). TZPIMS showed a CO_2_/CH_4_ selectivity of ~17.5 at 10 atm of CO_2_ partial pressure in pure-gas measurements and 37.5 for the equimolar CO_2_/N_2_ mixed-gas selectivity. This enhancement in selectivity has been hypothesized to be due to the solubility effects. Therefore, these PIMs can sustain their separation performance in a mixed-gas environment. More work is needed to further examine the effect of contaminants on mixed-gas permeation properties, particularly on physical aging at thicknesses of 1 micron or less, suitable molecular weights, and mechanical behaviour of PIMs [23].

Membrane performance improvement, including aging issues, in high free-volume polymers have been studied, such as via polymer backbone redesign, but this method involves complex synthesis steps and produces low yields. Other methods to improve high free-volume polymer aging issues are post-synthetic modification, cross-linking [112], heat treatment, blending of nanomaterials [134], and combination of the above methods with the target to improve the rigidity of all three factors of the polymer chain, interchain spacing, and interchain interactions. Improved aging-resistance membranes can be achieved by four general methods, which are by slowing the aging rate, improving initial gas permeability with similar aging rate, hence reaching stability at higher permeability value, modified membranes with faster aging rate for large pores and slower for small pores, and finally to use strongly interacting substituents (e.g., hydroxyl) to lock the free volume by hydrogen bonding [16].

Genduso et al. [37] examined ladder polymer (PIM-Trip-TB) and PIM-1 resistance to plasticization since it has high intrachain rigidity. Intrachain rigidity can be obtained if the movement around the chemical bonds of the repeating unit is constrained, for example, by ladder design or by introducing sites of substitution that lock the local rotation of certain building blocks of the repeating unit. However, they found that the intrachain rigidity cannot restrain the unfavourable mixture effects on CO_2_/CH_4_ diffusion and permeability selectivity, since the results are also the same as CTA, where the membrane is thermodynamically stable but kinetically disturbed. The mixed-gas solubilities were lesser than pure gas due to competitive sorption, but the mixed-gas solubility selectivity coincides with the average value at infinite dilution, and at all pressures is higher than pure-gas solubility selectivity. The diffusion coefficient is found to be insensitive to mixture effects, but the diffusion coefficient of CH_4_ in the mixture is higher, subsequently lowering the CO_2_/CH_4_ permselectivity in the mixture due to CO_2_-induced plasticization. The increase in CH_4_ mixed-gas and higher-pressure diffusion coefficients from the pure-gas values are more pronounced for both PIMs than for the conventional 6FDA-mPDA PI. It was concluded that intrachain rigidity cannot solve the plasticization problem and must be supported by interchain rigidity, such as promoting chain-induced hydrogen bonding. This can be achieved by integration of an amidoxime moiety in the PIM-1 structure via cross-linking, and by polymer heat treatment (CMS, TR polymers), which are found to be successful approaches against plasticization [37].

Stern’s group incorporated polar functionalities such as hydroxyl (OH) and carboxyl (COOH) groups in the polymer backbone and observed higher CO_2_/CH_4_ permeability selectivities even 2- to 3-fold better than that of commercial CTA [138]. Alaslai et al. [41] investigated the possibility of tightening polymer structure for CO_2_ plasticization resistance under high-pressure mixed-gas conditions using PIs membrane. They have introduced the hydroxyl groups in the diamine moiety of 6FDA-diaminophenol (DAP) and 6FDA-diamino resorcinol (DAR) PIs to increase the charge transfer complex formation compared to unfunctionalized 6FDA-m-phenylene diamine (mPDA). The hydroxyl-functionalized PIs 6FDA-DAP and 6FDA-DAR exhibited very high pure-gas CO_2_/CH_4_ selectivity of 92 and 94 with CO_2_ permeability of 11 and 8 Barrer, respectively, compared to 6FDA-mPDA values of 70 and 14 Barrer. It was concluded that hydroxyl-containing PI membranes maintained very high CO_2_/CH_4_ permselectivity due to a large increase in diffusivity selectivity, indicating CO_2_ plasticization resistance at high pressure (40 atm), mixed-gas (equimolar of CO_2_ and CH_4_) conditions with a reduction in permeability. This is due to stronger chain interactions by interchain hydrogen bonding and charge transfer complex (CTC) formation that can reduce the plasticization effect of CO_2_. The increase in tightness of polymer chains decreased the diffusion coefficient, increasing the diffusivity selectivity compared with that of the unfunctionalized 6FDA-mPDA. The CO_2_/CH_4_ diffusivity of 6FDA-mPDA was increased from 13 to ~21 upon the introduction of hydroxyl groups, whereas the CO_2_/CH_4_ solubility selectivity varied only between 4.8 and 4.9 [41]. However, the advantage of high permeability has been sacrificed significantly to improve selectivity and plasticization resistance.

Abdulhamid et al. [138] explored a novel trimethyl-substituted carboxyl-containing PI (6FDA-TrMCA) with performance located on the 2018 mixed-gas upper bound with a CO_2_ permeability of ~98 Barrer and CO_2_/CH_4_ permselectivity of 38. The COOH-functionalized PIM-PI homopolymer is claimed to have outstanding resistance against plasticization by CO_2_ at least at 10 atm CO_2_ partial pressures, due to interchain hydrogen bonding and CTC formation induced by the carboxyl group. The trimethyl substitution with diamine building blocks promoted the formation of large, unrelaxed, free volumes, with a BET surface area of 260 m^2^g^−1^, which is typical of PIM-PI [138]. Ma et al. [141] developed novel polymers (hydroxyl-functionalized PIM-PI, namely PIM-PMDA-OH and PIM-6FDA-OH) which integrate significant microporosity and polar hydroxyl groups. They have high thermal stability, easy processability for membranes, good solubility, and display good permeability owing to the intrinsic microporosity introduced by the highly contorted PIM segments, as well as high CO_2_/CH_4_ selectivity that arises from the hydroxyl groups. PIMs with contorted ladder-like backbones consisting of spiro-centres and rigid, fused dioxane rings prevent close polymer-chain packing. These highly glassy polymers contain alternating double bonds in the main chain and bulky side-chain substituents that prevent efficient chain packing leading to high free volume. PIs, on the other hand, are chosen since they can be functionalized to alter their affinities to different gases, and the introduction of hydroxyl groups can improve the selectivity. The resultant PIM-PI membranes showed CO_2_/CH_4_ selectivities of >20 when tested with equimolar CO_2_/CH_4_ mixture for feed pressures up to 50 bar [141]. They also showed better plasticization resistance by showing high CO_2_ permeability and CO_2_/CH_4_ selectivity under high-pressure, mixed-gas conditions. On top of that, the incorporation of hydrophilic hydroxyl groups and microporosity in the polymers increases their water affinity. As such, the higher water-vapor sorption capacity makes them potentially suitable for gas dehydration applications [141].

The rigid polymers are proposed for aggressive natural gases applications. It is suggested to use scalable molecular sieves with advanced polymers such as TR and PIMs, or hybrid materials using MOF, since the molecular sieve is believed to be unaffected by plasticization but still shows a competition effect between mixture components for transport pathways and can improve the membrane’s entropic selectivity [29].

##### PEO and PEG

A polymer with the same repeating units is called a homopolymer, either using single or more monomers, as long as the repeating units are the same throughout. A copolymer is the one that has been prepared from two different monomers that are coupled using various methods, and the sequence of structural units are completely irregular. The copolymer can be further classified as random, block, or graft [42]. Poly(ethylene oxide) or poly(ethylene glycol), known as PEO or PEG, is a rubbery polymer prepared by oxidation of ethylene and then reaction with water. The material is named PEG for its low molecular weight (lower than 20,000 g/mol) and is usually present in liquid form. PEO for higher molecular weight is present in solid form, although some exceptions have been observed. The crystallinity of this material with an increment of molecular weight relates to its Tg, which varies from −15 to −95 °C. The gas permeates better with lower molecular weight and vice versa for the higher molecular weight with denser inter-segmental chain packing. Despite this, the effect of chain flexibility on permeability increment for higher molecular weight is superior to permeability decrease due to crystallinity enhancement [4].

The presence of polar oxygen and ether groups in the polymer structure has improved the acid gas separation performance since they have a strong attraction to CO_2_ gas. This is due to the dipole moment of the ether segment and the quadrupole moment of CO_2_. Poly(vinyl pyrrolidone) PVP and PEO oligomers, which are water-soluble polymers, could be added in a small amount to optimize the porous structure [23]. Hence, these polymers are suitable for the separation of gas mixtures containing polar gases (such as CO_2_ and H_2_S) and nonpolar ones (such as H_2_, N_2_, and CH_4_). This is due to their polarity and CO_2_ affinity, resulting from etheric groups in PEG. PEO comprises an ether–oxygen functional group which preferably interacts with CO_2,_ leading to an increase in gas selectivity [83]. However, it is challenging to make a defect-free film of PEO since it has a strong inclination to crystallize, which will significantly reduce the gas permeability. Methods to lessen crystallinity in PEO include designing a highly branched chain, cross-linked networks with a high concentration of PEO, using the low molecular weight of liquid PEO or PEG and by designing phase-separated block copolymers with ethylene oxide (EO) segments, which are too short to crystallize effectively at room temperature [18]. Low-molecular-weight PEG serves as a pore-forming agent and pore size enhancer, while high-molecular-weight PEG acts as a pore-reducing agent and pore size reducer. Increases in pore size, pore density, sublayer porosity and FFV, enhance permeance while reducing the selectivity. Improvements in plasticizing resistance and flexibility of polymer chains also lead to higher diffusion [50]. Studies on how to further enhance the plasticization pressure of these classes of highly permeable polymers are suggested for future research [83].

The materials that comprise a hard portion (such as polyamide or terephthalate) and a soft portion (such as polyether or PEO) are PEBAX, polyactive, and many others. The semicrystalline, semiamorphous structure of these materials results in better selectivity and CO_2_ permeability [4]. If polymers are soluble in each other, such as the combination of glassy and rubbery, an optimized synergy can be harnessed. Karimi et al. [142] has tried to incorporate PEG in a PSU polymer with hard segments. It was found that this approach can improve the membrane permeability. The same finding been reported by Yave et al. [143] and Car et al. [144] for PEBAX/PEG. Findings by Kawakami et al. [145], indicated that by adding PEG to cellulose nitrate it is possible to increase the solubility selectivity due to strong interaction and high affinity of PEG to CO_2_. A similar finding was stated by Okamoto et al. [146] and Yoshino et al. [147] for PI membrane.

Yu et al. [148] prepared a polyethersulfone (PES) copolymer with PEG side chains via polycondensation followed by the thiolene click chemistry. The optimization of the PES-g-PEG membrane is achieved using a variety of densities and lengths of PEG side chains (Mn = 550, 1000, 2000) to fine-tune the structure of the microphase separation. Thermal annealing helps the copolymer membrane with shorter PEG side chains to achieve higher chain density and more developed microphase separation. The resultant membrane shows the best gas-separation performance, with 26.8 Barrer and 27.6 CO_2_ permeability and selectivity. The permeability variation mostly depends on side-chain density rather than side chain molecular weight and volume fraction. Lee et al. [149] reported a plasticization-resistant membrane using PBEM-G-POEM (poly(2-[3-(2H-52enzotriazole-2-yl)-4-hydroxyphenyl] ethyl methacrylate) and poly(oxyethylene methacryalate) comb copolymer that was synthesized using radical polymerization. The CO_2_ permeance did not change much with pressure when the PBEM content was above 20 wt%. The best performance was achieved with a PBEM:POEM ratio of 20:80 wt%. The CO_2_ permeance and CO_2_/N_2_ selectivity were 29.3 GPU and 73.3, respectively, which were much better than that of the composite membrane prepared using commercially available PEBAX (CO_2_ permeance: 22.9 GPU, CO_2_/N_2_ selectivity: 21.8). Recently, a series of amphiphilic tercopolymers comprising polyacrylonitrile, poly(ethylene glycol) methyl ether methacrylate (PEGMA), and poly(N,N-dimethylamino ethyl methacrylate) (PDMAEMA) segments have been synthesized via a facile free-radical polymerization. By optimizing the content and chain length of the PEGMA segments, the optimized membranes exhibited a high CO_2_ permeability of 47 Barrer with CO_2_/N_2_ and CO_2_/CH_4_ selectivities of 67 and 23, respectively.

PEO used in copolymerisation act as the flexible soft segment that can decrease the polymer crystallinity and improve FFV and separation performance. The PEO within the copolymer structure enhances diffusivity and solubility for CO_2_ polar gas due to the strong interaction with the polar functional group in the PEO segments. On the other hand, the lower mechanical strength of PEO can be improved through bonding with a hard portion in the polymer backbone, hence contributing to membrane performance enhancement [4].

##### PTMSP

Glassy polymer PTMSP has shown an extraordinary capability to perform separation similar to rubbery material, where heavy hydrocarbons permeate at a higher rate than methane. It has very high free volume (up to 30%) and large surface areas which exceed 1000 m^2^/g. In single-gas testing, it has a very low selectivity for separating organic vapours from gases, but higher selectivity is attained with gas mixture. This is because the surface diffusion is used as the gas transport rather than solution diffusion, and the condensable vapours sorb onto the wall resulting in multilayer adsorption, probably including capillary condensation. The build up of heavier hydrocarbons lessens the permeation of noncondensable gas [14,39]. Since PTMSP is soluble in hydrocarbons, it is not resistant to solvent [14]. The high permeability quickly reduces throughout operation due to densification and structural compaction. The aging of polyacetylene such as PTMSP was identified over 30 years ago, and it remains a major issue alongside superior permeation traits of polymers, where almost one-third of them are empty volume [16].

Several efforts have been made to resolve the PTMSP aging issue, mainly via chemical cross-linking and the addition of inorganic nanoparticles which have a rigid structure. Nevertheless, these have been unsuccessful attempts, with a 70% gas permeability reduction after PTMSP cross-linking [39]. Bazhenov et al. [150], tried to improve this issue by using it as the membrane support in a thin film composite (TFC), which can contribute to a high CO_2_-permeance membrane. PTMSP, which is insoluble in chloroform, has been used as a gutter layer for further deposition with CO_2_-selective materials from organic solvents. Using poly (ethyl-eneglycol) diglycidyl ether (PEGDGE) as the cross-linking agents, PTMSP has been cross-linked with polyethyleneimine (PEI) using the kiss-coating technique. The procedure of using alcohol as post-treatment is used for the improvement of gas permeance. The resulting membranes have CO_2_ permeance of 18,500–20,000 GPU and 3.6–3.7 ideal CO_2_/N_2_ selectivity. Therefore, this method shows the potential of the membrane as a good support material to be used with another CO_2_-selective layer. The incorporation of porous aromatic framework (PAF), an ultraporous additive, has been explored to stop aging in super glassy polymers, such as PTMSP with poly(methylpentyne) (PMP). The polymer chain portion is assimilated in the PAF pores to freeze the polymer with its originally open and loosely packed features [39].

Midoxime-functionalized poly(1-trimethylsilyl-1-propyne) (AO-PTMSP) membrane has been prepared by Feng et al. [151] through hydrosilylation and post-polymerization modification. Substantial enhancements in CO_2_/N_2_ gas separation performance have been achieved with CO_2_ permeability of ~6000 Barrer and CO_2_/N_2_ selectivity of ~17 compared to neat PTMSP membranes. This is hypothesized due to enhancement in the interaction between the gas and polymer. The high potential of the PTMSP membrane has attracted Air Products and MTR to market it particularly for the separation of heavy hydrocarbons, which is the next largest market after natural gas sweetening using membranes in natural gas processing [14].

##### Perfluorinated Polymer

Perfluoropolymers are unique amorphous glassy polymers that have high gas permeability resulting from its large free volume, high electronegativity and strong hydrophobicity [152]. Most or all of its C-H bonds (346 kJ/mol) have been replaced by C-F bonds (487 kJ/mol), which are stronger [17], improving its chemical, thermal, and plasticization strength to both CO_2_ and hydrocarbons [14]. Their transport mechanisms are dominated by solubility in a polymer with gas condensability [35]. Perfluoropolymers have exhibited superior resistance to aromatic and other hydrocarbon gases since the dominance mechanism is solubility [57]. These polymers possess an inert nature due to the perfluoro chemistry, and retain a glassy nature even at high partial pressure organic vapor [40]. However, selectivity was low, and further optimization is needed [62].

Chiang et al. [153] investigated the blend of poly(perfluoro-2-methylene-4-methyl-1,3dioxolane) or poly (PFMMD) with perfluoropolyether as the plasticizer. The copolymer of poly (PFMMD)-co-CTFE (chlorotrifluoroethylene) managed to increase the selectivity from 26 to >40, and as the trade-off, the copolymer permeability was reduced. For the poly (PMMD)-PFPE (perfluoropolyethers), CO_2_ permeance has successfully been increased from 520 to 657 Barrer with only a slight reduction in selectivity.

**Figure 16:**
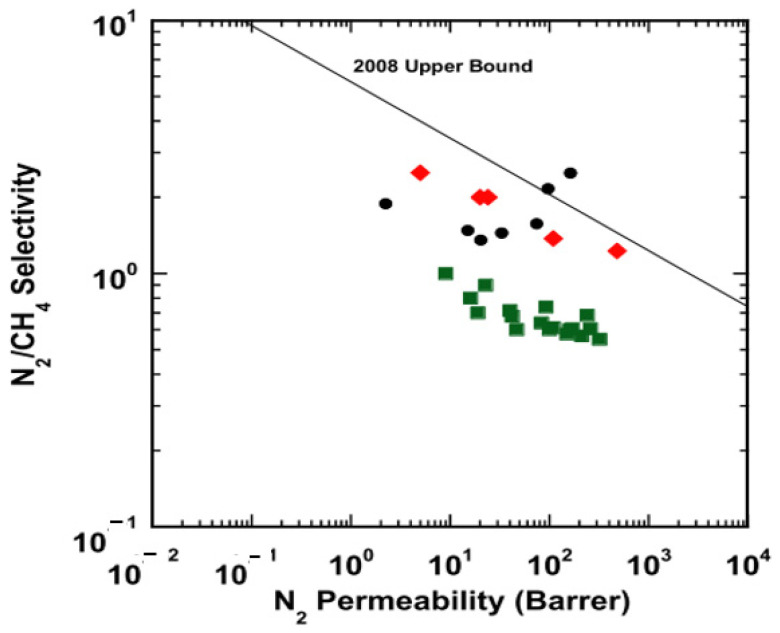
N_2_/CH_4_ separation performance for TR polymers (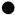), PIMs (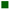), Perfluoropolymers (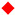) [23].

A key discovery for the gas-separation membrane is the invention of Teflon AF, by DuPont in the late 1980s. Other amorphous glassy perfluoropolymers were also developed, namely Cytop (by the Asahi Glass Company) and Hyflon AD60 and AD80 (produced by Solvay) perfluoropolymers with well-known superior performance surpassing the 2008 Robeson boundary (refer Figure 16), [154,155]. Their chemical structure is shown in Table 7.

Fluoropolymers have many beneficial characteristics, but they are difficult to process by the normal methods. This is a crucial matter with perfluoropolymers, in which the solvent has been retained and has even undergone annealing. They are insoluble in common organic solvents and only soluble in perfluorinated solvents [152].

Furthermore, a high free-volume polymer will also have a higher aging issue. Additionally, the perfluoro polymer precursor material is very expensive, resulting in high fabrication costs. Formation of fluoropolymer skin on top of the hydrocarbon polymer support, such as the fluorination of Matrimid, has been introduced. This method tried to exploit the synergy between the C-F bond resistance to plasticization and the conventional hydrocarbon polymer. The remarkable performance and unique properties of this membrane will make it attractive for future research and innovation. More work is to be conducted to improve its limitations, as discussed [58].

In this paper, we have categorized the polymeric membrane for CO_2_/CH_4_ gas separation improvement strategies into five categories, reviewed the methodologies attempted by various researchers and discussed each method’s achievements, limitations and areas of improvement needed. The summary is presented in Table 8.

## 5. Future Direction

This review has covered the cause, effect, and potential solutions to enhance polymeric gas-separation membrane resistance to aging and plasticization for CO_2_ removal from the natural gas application. At present, the existing membrane materials, namely CA, PSF, PC and PI, suffer from these phenomena responsible for their low performance and reduced membrane life during commercial application. Numerous new families of polymers have been designed and explored to enhance permeability and selectivity, and some of these polymers also have higher plasticization resistance. These polymers include TR polymers, RTILs, PIMs, perfluoropolymers, and the latest modified PIs, which are promising materials for membrane gas separation.

PIs have been the material of choice for many researchers after various modifications of the polymer backbone and functional groups. TR polymers and PIMs typically show the best separation performance when plotted in Robeson’s upper bound. These polymers are quite new for gas separations, hence further research may improve their transport properties and mechanical strength. FTMs display less favourable performance than other new materials but still show great potential than current commercial materials. Further development is needed to reduce its limitation for high-pressure applications. Perfluoropolymers show permeability and selectivity values that are specifically encouraging for N_2_/CH_4_ separations. The robust properties of perfluoropolymers have shown great potential for gas separation applications in the future. The focus of research for this polymer should be directed toward achieving greater selectivity.

It was commonly detected that functional groups such as polar ether–oxygen linkages, hydroxyl, carboxyl, and amine improve the separation performance of polymeric membranes. The interaction with CO_2_ enhances its solubility, hence the solubility selectivity of the membrane is improved. The size of such functional groups contributes to the enhancement of CO_2_ diffusivity, which leads to higher diffusivity-selectivity of the membranes. It was also discovered that porogens such as sulfonic, carboxylic acid, cyclodextrin, and saccharides could potentially improve the separation properties of polymeric membranes. Therefore, the incorporation of any of functional groups into the polymer backbone is one of the excellent approaches for producing membranes with superior performance.

MMM still have some essential glitches that need to be resolved to enable their commercialisation. Although MMM have been confirmed to have some performance enhancements, it was observed that most of them experienced poor bonds between the organic matrix and inorganic particles. Therefore, the industry focus will still be on the polymeric membrane studies due to cheaper material, easy processing into the module, moderate performance and lifetime, lightweight nature, high surface-area-to-volume ratio, and many other advantages.

Novel materials and complex synthesis methodology make mass production expensive. For this reason, research efforts should still be focused on designing readily processable polymers with some simple treatment or modification that is practical for production, with the target to have a good combination net effect of permeability and selectivity, with long-term stability and high resistance to plasticization and aging. All these will have a counterbalancing effect among each other, including the competitive sorption phenomena during multicomponent testing with contaminants.

Scaled-up fundamental knowledge and issues need to be studied for a complex mixture consisting of multicomponents, including various contaminants at high pressure. This know-how is very vital for the scale-up of membrane technology, which can accelerate the commercialisation of plenty of new polymer membranes developed in the literature. Focusing on high-selectivity and high-permeability membranes using simple gas has not guaranteed that the membrane can survive during actual operating conditions, since the CO_2_, heavy hydrocarbon, aromatics and many others can swell the membrane and reduce selectivity, while competitive sorption, compaction, densification, and aging reduce the permeability with the enhancement of selectivity.

Those working in academic and industrial research and development could collaborate to create representation mixtures for common separations that include the main feed components and typical contaminants for more reliable results close to commercial application. Generally, an effective solution to the plasticization and aging issues enables steady performance for the separation of gases that are potentially superior to those applied in the commercial application. If these membranes’ performances can be maintained over 3 years of lifetime when applied to thin (<1 micron) skin of asymmetric or composite membranes, it would show a major breakthrough in the current membrane technology achievements. Depending on the conditions, it might be essential and practical to reduce separation performance slightly in order to increase the mechanical strength and robustness towards aging and plasticization for long-term stable performance.

For a medium-pressure and low-temperature application, which is beneficial in terms of aging (longer lifetime), it is crucial to leverage on the good CO_2_ plasticization and solubility selectivity to enhance the CO_2_ permeance during the operation before the aging and densification take place, which will dramatically impact the diffusivity selectivity. For a high-pressure and high-temperature operation, the diffusivity selectivity is favourable, but the membrane will suffer from aging and shorter lifetime. The development of membranes to overcome the permeability–selectivity trade-off is an ongoing task.

Besides the experimental studies for membrane material improvement, molecular dynamics studies will be able to provide a better picture of overall separation and might assist the researcher in developing molecularly engineered chemical structures with high permeability and selectivity. Advanced simulation and modelling are critical for an improved understanding of performance, properties, and structural relationships in the new and existing membrane material. Computational studies are required to gain a thorough understanding of the morphology and the separation mechanism of nonporous amorphous polymer membranes, hence providing a more detailed correlation with the experimental findings. When applied to super glassy polymers, these can provide more information on the distributions of the free volume. The prediction of small gaseous permeation behaviour, sorption isotherms calculations, and free volume distributions determination can also be made using force-field-based molecular mechanics and molecular dynamic (MD) methods. Previously, membrane research was purely based on empirical observation, then it moved to mathematical modelling and molecular modelling, and now it has been advanced into the new method called machine learning (ML). Instead of relying on exhaustive, expensive, and time-consuming experimental investigations, the vast available data in the literature has been trained into an algorithm that derives the permeability calculation by using thorough information of the monomer structure and chemistry. The algorithm accuracy has been tested by validating it with the most promising polymer membranes, which has been confirmed to exceed the upper bound of CO_2_/CH_4_ separation performance.

## 6. Conclusions

Membranes are among the separation technologies with huge potential. This review addresses the key challenges in the application of polymeric technology for CO_2_ separation, focusing on plasticization and aging. A brief introduction on the properties and limitations of the current commercial polymeric membranes is deliberated. The effect of each plasticizer component in natural gas on membrane performance and the relationship between operating conditions and the membrane efficiency were discussed. The recent technological advancements and techniques to overcome plasticization and aging issues, covering polymer modification, new, high free-volume polymers, polymer blending and FTMs have been highlighted. We have covered the cause, effect, and potential solutions to enhance polymeric gas-separation membrane resistance to aging and plasticization for CO_2_ removal from natural gas applications. At present, the existing membrane material suffers from plasticization and aging phenomena, which are responsible for their low performance and reduced membrane life during commercial application. Numerous new families of polymers have been invented to enhance permeability and selectivity, and some of these polymers also have higher plasticization resistance. These polymers include TR polymers, RTILs, PIMs, perfluoropolymers, and the latest modified PIs, which are promising materials for membrane gas separation with stable long-term performance. Upcoming research must focus on mixed gas with CO_2_ including minor condensable contaminants as per real natural gas, to determine the competitive sorption effect on CO_2_ permeability and membrane selectivity. The effect of pore blocking, plasticization and aging should be given emphasis to cater for large-scale applications.

## Figures and Tables

**Table 1:** Polymeric membrane producers for CO_2_ separation available in the market [11].

Producer	CO_2_ Separation Process
IGS (Generon membrane) DIVEX GLOBAL (Helipur membrane)	CO_2_/H_2_ CO_2_ from N_2_/Ar/O_2_
UOP—A Honeywell Company (-)	CO_2_/H_2_
AIRRANE Co, Ltd. (Hollow fiber polysulfone (PSF)-based membrane)	CO_2_ from CH_4_/H_2_/N_2_
CYNARA (Cellulose triacetate (CTA) based)	CO_2_ from natural gas
MEDAL (Polyaramide based) GRACE, SEPAREX (Cellulose acetate (CA) MTR (Perfluoro polymers based) PBI Performance Products, Inc. (Hollow fiber Celazole^®^PBI based)	CO_2_/H_2_
AIR Liquide S.A. (-)	CO_2_ from biogas/natural gas
EVONIK Industries AG (Sepuran^®^ membranes) Compact Membranes Systems, Inc. (Fluoropolymer based)	CO_2_ from biogas CO_2_/CH_4_, CO_2_/N_2_

**Table 2:** Chemical structure and Tg for various polymers.

No.	Polymer Name	Tg (°C)	Chemical Structure
(a)	Cellulose & Cellulose Tri-Acetate (CTA)	185	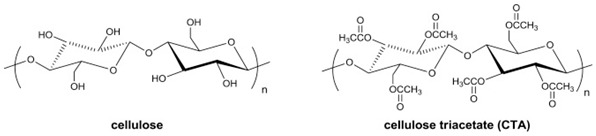
(b)	Polysulfone (PSF)	182	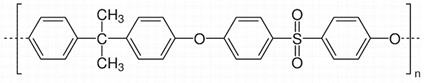
(c)	Polyethersulfone (PES)	222	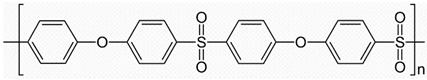
(d)	Poly trimethyl phenylene ethersulfone (TPES)	NA	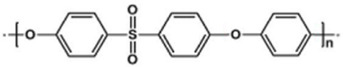
(e)	Polyetherimide—PEI (Ultem^®^) (BPADA-PPD) [45]	199	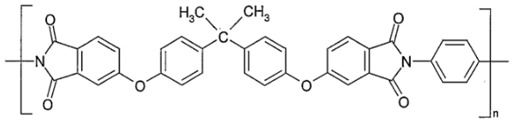
(f)	Polyimide—PI (Matrimide^®^ 5218) (BTDA-DAPI)	313	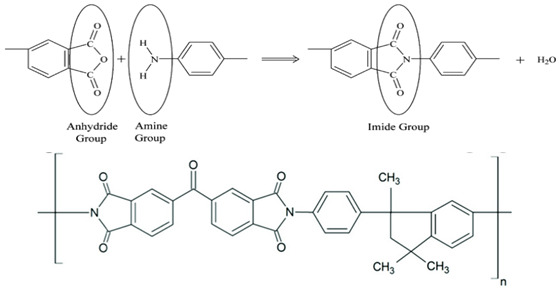
(g)	Polyamide-imide—PAI (Torlon)	281	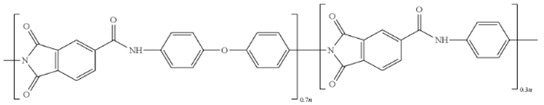
(h)	6FDA-based polyimide	348	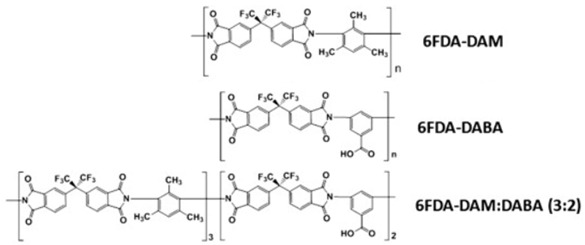
(i)	Polycarbonate Bisphenol Z & A (BPZ-PC & BPA-PC)	175 & 151	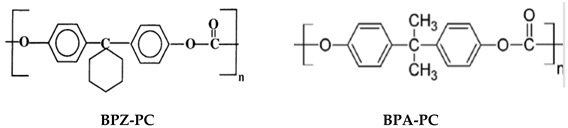
(j)	Polymer of intrinsic microporosity-1 (PIM-1)	442	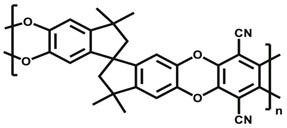
(k)	Hyflon	194	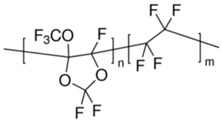

**Table 3:** Effects of plasticization, competitive sorption and aging, that counterbalance each other.

No	Counterbalance Effect	Permeability	Selectivity	Tg	FFV
1	Plasticization	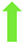	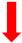	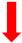	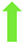
2	Competitive sorption	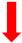	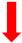	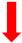	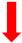
3	Aging	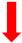	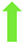	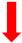	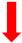
4	Net Effect	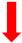	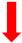	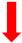	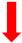

**Table 4:** Summary of polymer-modification improvement strategies.

No.	Modification Routes	Improvement Strategies/Reaction Schemes
1	Incorporation of CO_2_-philic groups into the polymer to improve polarity towards CO_2_. Oxygen functionalities can improve sorption properties. The interaction can be via hydrogen bonding or electrostatic.	Incorporation of various Lewis bases/pendant polar groups such as ether, hydroxyl, carboxylic and carbonyl oxygen promotes physical interaction with CO_2_ (electronegativity) due to higher polarity, thus producing higher CO_2_ solubility. It can produce extra hydrogen bonding that further alters the pore size of the membranes, resulting in higher activation energy for CO_2_ and CH_4_. The incorporation of micropores from the incorporation of polar groups have enhanced the permeability of CO_2_. 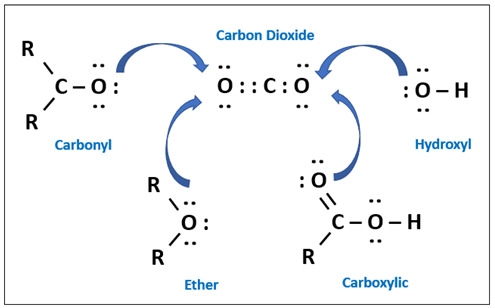
2	Incorporation of bulky groups to improve chain stiffness and limit chain packing	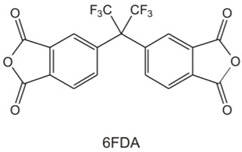 Bulky hexafluoro-substituted carbon (–C(CF_3_)^2^) groups in dianhydride structure of 6FDA-based PIs increased chain stiffness, hence increasing selectivity while inhibiting chain packing since the bulky group can serve as a molecular spacer which can increase the permeability and reduce aging. 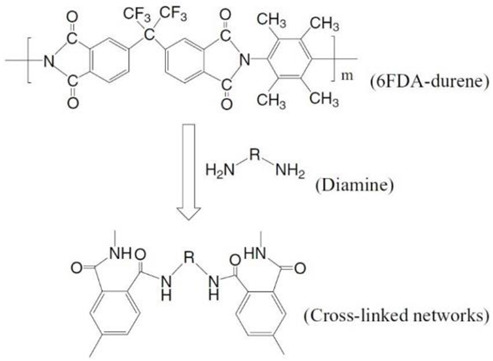 Bulky diamines help disrupt chain packing while increasing free volume. This contributes to the enhancement in permeability, while the aromatic rings are responsible for chain rigidity for better selectivity [25] and aging resistance. Amines strongly and selectively bind the CO_2_ via chemisorption leading to higher heat of adsorption [86]. Bulky tetra-o-isopropyl and naphthalene groups are introduced to membranes, resulting in high FFV values and enhanced polymeric backbone rigidity. The disturbed chain packing leads to high gas permeabilities [87] and aging resistance. Rigid, bulky and diamond-like structure Adamantane is grafted into the membrane main chain and side chain through a simple acyl chloride-substitution reaction to adjust the chain packing. D-spacing of the membranes could be finely tuned by adjusting the mole ratio of grafted adamantane moiety. The resultant membrane CO_2_ permeability is enhanced significantly [88].
3	Functionalization using bromination to increase FFV and thus permeability.	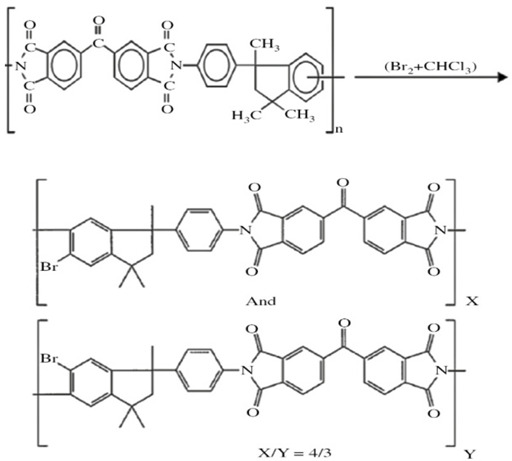 Brominated Matrimid 5218 membranes were much more permeable due to the higher FFV of the brominated membranes [89].
4	Sulfonation to improve rigidity and FFV	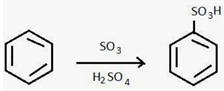 Sulfonation reaction successfully enhanced the microvoids, promoted interchain hydrogen bonding by the SO_3_H group and boosted the rigidity. As the degree of sulfonation increased, gas permeability increased by 3.2-fold [83] and improved aging resistance.
5	Lithiation to improve solubility, FFV and chain rigidity	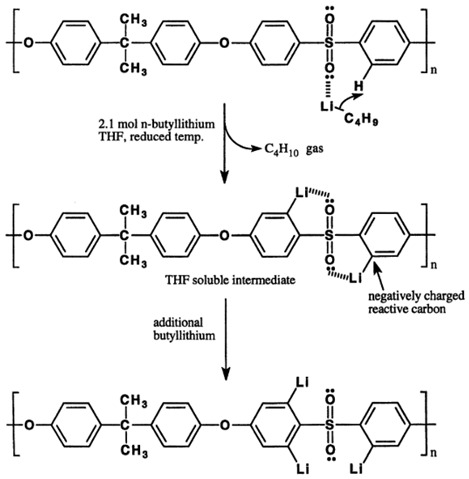 Lithiation is performed in PSF due to the availability of strong electron-withdrawing effect and capability of the lone pairs of electrons on the sulfone oxygen atoms to combine with the lithiation agent. Butyllithium substitutes the ortho-sulfone hydrogen atoms with lithium atoms so that the aromatic carbon atoms to which lithium is attached nominally have a negative charge, which can improve its performance and enhance attraction towards CO_2_ [90].
6	Esterification by diols or polyols	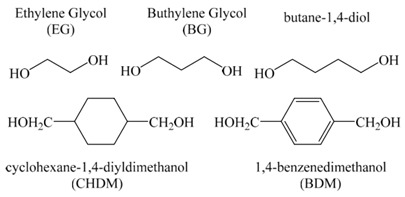 Common diol cross-linkers can be introduced through carboxylic acid or sulfonic acid groups to form ester bonds, linking the two polymer chains [3]. Esterification, which involves the reaction between acid and alcohol, serves as a function to create limited rotational ability [91].

**Table 5:** Performance comparison of some recently reported FTMs.

Polymer	Carrier	Condition	Gas Pair	Separation Performance	Neat	References
Pebax	Aniline molecule (50 wt%)	7 bar, 25 °C	CO_2_, N_2_ Binary CO_2_, N_2_ (20%, 80%)	α = 92.5; P_CO_2__ = 151 Barrer α = 68; P_CO_2__ = 123 Barrer	α = 22.2; P_CO_2__ = 75 Barrer α =19; P_CO_2__ = 83 Barrer	[122]
PES	[DMAPAH][F]- Fluorion-based protic ionic liquids (FPILs)	0.1 bar, 40 °C (water content 40%)	CO_2_, N_2_ CO_2_, CH_4_	α = 774; P_CO_2__ = 2572 Barrer α = 387; P_CO_2__ = 2572 Barrer	-	[123]
PVA-PEG-GA (Glutaraldehyde)	(PETIM)– Amine-rich poly(ether imine)	2 bar, 30 °C	CO_2_, N_2_	α = 56; P_CO_2__ = 207.7 Barrer	-	[124]
γ-Al_2_O_3_	[Emim] [Tf2N]-ionic liquid	1.5 bar, 30 °C	CO_2_, N_2_	α = 27; P_CO_2__ = 23 GPU	-	[125]
ILPPO	(Br-6-MPRD)– 1-bromohexyl-1 methylpiperidinium bromide	1 bar, 25 °C	CO_2_, N_2_ CO_2_, O_2_	α = 12.94; P_CO_2__ = 907 Barrer α = 14.9; P_CO_2__ = 907 Barrer	α = 6.21; P_CO_2__ = 70 Barrer α = 5.15; P_CO_2__ = 70 Barrer	[126]
PES	([bmim][PhO])1-butyl-3-methylimidazolium phenolate	0.1 bar, 40 °C	CO_2_, N_2_	α = 135; P_CO_2__ = 2020 Barrer (Dry) α = 127; P_CO_2__ = 2540 Barrer (Wet)	-	[3]
PES	[DMAPAH] [MOAc]	0.1 bar, 40 °C	CO_2_, N_2_	α = 66; P_CO_2__ = 1391 Barrer	-	[128]
PVDF	[Bmim][Ac] [Bmim][BF_4_]	0.1 bar, 30 °C	H_2_S/CH_4_ H_2_S/CO_2_ H_2_S/CH_4_ H_2_S/CO_2_	α = 142; P_H_2_S_ = 5279 Barrer α = 11.9; P_CO_2__ = 443 Barrer α = 40; P_H_2_S_ = 3708 Barrer α = 3.5; P_CO_2__ = 1056 Barrer		[129]
PIM-COP	[C6mim][Tf2N] embedded	-	CO_2_, N_2_	α = 30; P_CO_2__ = 800 Barrer	α = 19; P_CO_2__ = 7440 Barrer	[130]

**Table 6:** Summary of MMM performance for different gas pairs.

Polymer	Inorganic Filler	Condition	Gas Pair	Separation Performance	Neat	References
PIM-1	Graphene-like fillers	2 bar, 25 °C	Binary CO_2_, CH_4_ (50%, 50%)	P_CO_2__ = (3.5 ± 0.6) × 10^3^ Barrer α = 22.9 ± 1.1	P_CO_2__ = (2.0 ± 0.7) × 10^3^ Barrer α = 30.0 ± 4.7	[134]
PES	Carbon Molecular Sieve (CMS) and diethanolamine (DEA)	30 bar, 25 °C	CO_2_, CH_4_	P_CO_2__ = ~300 GPU α = 16.04	P_CO_2__ = ~140 GPU α = 8.15	[135]
MATRIMID 5218	AMP-RGO (Ascorbic acid multiphase reduced graphene oxide)	10 bar, 30 °C	CO_2_, CH_4_	P_CO_2__ = 10.7 Barrer α = 79.8	-	[136]
PI	ZIF-95	3 bar, 30 °C	CO_2_, CH_4_	P_CO_2__ = 23.2 ± 0.38 Barrer α = 58	P_CO_2__ = 5.7 ± 0.11 Barrer α = 33	[125]
PSF	Cloisite 15A	4 bar, 25 °C	CO_2_, N_2_	P_CO_2__ = 72.04 GPU α = 518.34	P_CO_2__ = 21.68 GPU α = 22.58	[137]

**Table 7:** Chemical structure of Selected Perfluoropolymers-Based Membranes.

Polymer	Chemical Structure
Hyflon AD60	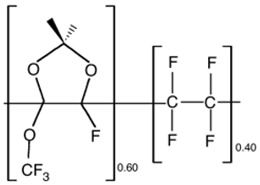
Hyflon AD80	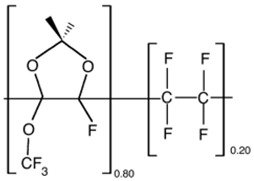
Cytop	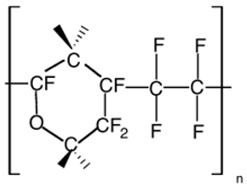

**Table 8:** Summary of recent polymeric gas-separation (CO_2_/CH_4_) membrane improvement strategies for plasticization and aging resistance.

Strategies	Achievement/Advantages	Issues/Enhancement Needed/Proposed
Polymer Modifcation via Cross-linking	Manage to improve performance and plasticization and aging resistance	To reduce complexity and improve on mechanical strength
Polymer Blending	Improvement within the average of the selected polymer performance accompanied by reduction in one aspect.	To improve the blended polymer performance further via cross-linking
FTMs	Manage to improve performance for low-pressure applications	To improve for high-pressure applications, to resolve the water/solvent evaporation and hydrocarbon fouling issues
MMMs	Manage to improve polymeric membrane performance and mechanical strength within the inorganic properties	To improve on sealing, homogeneity and agglomeration
New Polymer	Successfully improve on permeability and plasticization resistance to a great extent	To improve on selectivity and aging

## References

[1] (2021). Global Greenhouse Gas Emissions Data; US Environmental Protection Agency. https://www.epa.gov/ghgemissions/global-greenhouse-gas-emissions-data.

[2] Faramawy S., Zaki T., Sakr A.A. (2016). Natural gas origin, composition, and processing: A review. J. Nat. Gas Sci. Eng..

[3] Li P., Hosseini S.S., Zhang M., Deng L., Xiang D., Cao B. (2019). Approaches to Suppress CO_2_-Induced Plasticization of Polyimide Membranes in Gas. Processes.

[4] Kargari A., Rezaeinia S. (2019). State-of-the-art mod fi cation of polymeric membranes by PEO and PEG for carbon dioxide separation: A review of the current status and future perspectives. J. Ind. Eng. Chem..

[5] Adewole J.K., Ahmad A.L., Ismail S., Leo C.P. (2013). International Journal of Greenhouse Gas Control Current challenges in membrane separation of CO_2_ from natural gas: A review. Int. J. Greenh. Gas Control.

[6] Zhang Y., Sunarso J., Liu S., Wang R. (2013). Current status and development of membranes for CO_2_/CH_4_ separation: A review. Int. J. Greenh. Gas Control.

[7] Mushtaq A., Mukhtar H.B., Shariff A.M., Mannan H.A. (2013). A Review: Development of Polymeric Blend Membrane for Removal of CO_2_ from Natural Gas. Int. J. Eng. Technol. IJET-IJENS.

[8] Bernardo P., Clarizia G. (2013). 30 Years of Membrane Technology for Gas Separation. Chem. Eng. Trans..

[9] Sholl D.S., Lively R.P. (2016). Seven chemical separations to change the world. Nature.

[10] Iarikov D.D., Ted Oyama S. (2011). Review of CO_2_/CH_4_ Separation Membranes. Membrane Science and Technology.

[11] Russo F., Galiano F., Iulianelli A., Basile A., Figoli A. (2021). Biopolymers for sustainable membranes in CO_2_ separation: A review. Fuel Process. Technol..

[12] Wu H., Li Q., Sheng M. (2021). Membrane technology for CO_2_ capture: From pilot-scale investigation of two-stage plant to actual design. J. Memb. Sci..

[13] Bernardo P., Drioli E., Golemme G. (2009). Membrane gas separation: A review/state of the art. Ind. Eng. Chem. Res..

[14] Kentish S.E., Scholes C.A., Stevens G.W. (2008). Carbon Dioxide Separation through Polymeric Membrane Systems for Flue Gas Applications. Recent Patents Chem. Eng..

[15] Schmeling N., Konietzny R., Sieffert D., Rölling P., Staudt C. (2010). Functionalized copolyimide membranes for the separation of gaseous and liquid mixtures. Beilstein J. Org. Chem..

[16] Low Z., Budd P.M., Mckeown N.B., Patterson D.A. (2018). Gas Permeation Properties, Physical Aging, and Its Mitigation in High Free Volume Glassy Polymers. Chem. Rev..

[17] Han Y., Ho W.S.W. (2021). Polymeric membranes for CO_2_ separation and capture. J. Memb. Sci..

[18] Lin H., Freeman B.D. (2005). Materials selection guidelines for membranes that remove CO_2_ from gas mixtures. J. Mol. Struct..

[19] Sridhar S., Smitha B., Aminabhavi T.M. (2007). Separation of carbon dioxide from natural gas mixtures through polymeric membranes—A review. Sep. Purif. Rev..

[20] Brunetti A., Scura F., Barbieri G., Drioli E. (2010). Membrane technologies for CO_2_ separation. J. Memb. Sci..

[21] Yeo Z.Y., Chew T.L., Zhu P.W., Mohamed A.R., Chai S.P. (2012). Conventional processes and membrane technology for carbon dioxide removal from natural gas: A review. J. Nat. Gas Chem..

[22] Ma X.-H., Yang S.-Y. (2018). Polyimide Gas Separation Membranes.

[23] Suleman M.S., Lau K.K., Yeong Y.F. (2016). Plasticization and Swelling in Polymeric Membranes in CO_2_ Removal from Natural Gas. Chem. Eng. Technol..

[24] Sanders D.F., Smith Z.P., Guo R., Robeson L.M., McGrath J.E., Paul D.R., Freeman B.D. (2013). Energy-efficient polymeric gas separation membranes for a sustainable future: A review. Polymer.

[25] Du N., Canada C., Park H.B., Dal-cin M., Canada C., Guiver M.D. (2012). Advances in high permeability polymeric membrane materials for CO_2_ separations. Energy Environ. Sci..

[26] Favvas E.P., Katsaros F.K., Papageorgiou S.K., Sapalidis A.A., Mitropoulos A.C. (2017). A review of the latest development of polyimide based membranes for CO_2_ separations. React. Funct. Polym..

[27] Lozano E., Pra P., Tejerina F., Herna A. (2008). Effect of Fractional Free Volume and T g on Gas Separation Through Membranes Made with Different Glassy Polymers. J. Appl. Polym. Sci..

[28] Ahmad M.Z. (2018). Synthesis and Characterization of Polyimide-Based Mixed Matrix Membranes for CO_2_/CH_4_ Separation/Mohd Zamidi Ahmad.

[29] Lam B., Wei M., Zhu L., Luo S., Guo R., Morisato A., Alexandridis P., Lin H. (2016). Cellulose triacetate doped with ionic liquids for membrane gas separation. Polymer.

[30] Koros W.J., Zhang C. (2017). Materials for next-generation molecularly selective synthetic membranes. Nat. Mater. Gr..

[31] Yong W.F., Chung T.S., Weber M., Maletzko C. (2018). New polyethersulfone (PESU) hollow fiber membranes for CO_2_ capture. J. Memb. Sci..

[32] Julian H., Wenten I.G. (2012). Polysulfone membranes for CO_2_/CH_4_ separation: State of the art. IOSR J. Eng..

[33] Lokhandwala K.A., Baker R.W. (2008). Natural Gas Processing with Membranes. Ind. Eng. Chem. Res..

[34] Baker R.W. (2002). Future Directions of Membrane Gas Separation Technology. Ind. Eng. Chem. Res..

[35] Buonomenna M.G. (2017). Membrane Separation of CO_2_ from Natural Gas. Recent Patents Mater. Sci..

[36] Adewole J.K., Ahmad A.L., Sultan A.S., Ismail S. (2015). Model-based analysis of polymeric membranes performance in high pressure CO_2_ removal from natural gas. J. Polym. Res..

[37] Genduso G., Wang Y., Ghanem B.S., Pinnau I. (2019). Permeation, sorption, and diffusion of CO_2_–CH_4_ mixtures in polymers of intrinsic microporosity: The effect of intrachain rigidity on plasticization resistance. J. Memb. Sci..

[38] Brunetti A., Tocci E., Cersosimo M., Sung J., Hee W., Geun J., Moo Y., Drioli E., Barbieri G. (2019). Mutual influence of mixed-gas permeation in thermally rearranged poly (benzoxazole- co -imide) polymer membranes. J. Memb. Sci..

[39] Galizia M., Chi W.S., Smith Z.P., Merkel T.C., Baker R.W., Freeman B.D. (2017). 50th Anniversary Perspective: Polymers and Mixed Matrix Membranes for Gas and Vapor Separation: A Review and Prospective Opportunities. Macromolecules.

[40] Norman N.L., Anthony G.F., Ho W.S., Matsuura T. (2008). .Advanced Membrane Technology and Applications.

[41] Alaslai N., Ghanem B., Alghunaimi F., Litwiller E., Pinnau I. (2016). Pure- and mixed-gas permeation properties of highly selective and plasticization resistant hydroxyl-diamine-based 6FDA polyimides for CO_2_/CH_4_ separation. J. Memb. Sci..

[42] Mulder M. (1996). Basic Principles of Membrane Technology.

[43] Lu H.T., Liu L., Kanehashi S., Scholes C.A., Kentish S.E. (2018). The impact of toluene and xylene on the performance of cellulose triacetate membranes for natural gas sweetening. J. Memb. Sci..

[44] Mazyan W., Ahmadi A., Ahmed H., Hoorfar M. (2016). Market and technology assessment of natural gas processing: A review. J. Nat. Gas Sci. Eng..

[45] Ricci E., Di E., Degli M., Liu L., Mensitieri G., Fabbri P., Kentish S.E., Grazia M., Angelis D. (2021). Towards a systematic determination of multicomponent gas separation with membranes: The case of CO_2_/CH_4_ in cellulose acetates. J. Memb. Sci..

[46] McKeen L.W. (2008). The Effect of Temperature and Other factors on Plastics and Elastomers.

[47] Visser T. (2006). Mixed Gas Plasticization Phenomena in Asymmetric Membranes. Ph.D. Thesis.

[48] Genduso G., Pinnau I. (2020). Quantification of sorption, diffusion, and plasticization properties of cellulose triacetate films under mixed-gas CO_2_/CH_4_ environment. J. Memb. Sci..

[49] Serbanescu O.S., Voicu S.I., Thakur V.K. (2020). Polysulfone functionalized membranes: Properties and challenges. Mater. Today Chem..

[50] Scholes C.A., Kentish S.E., Stevens G.W. (2009). Effects of minor components in carbon dioxide capture using polymeric gas separation membranes. Sep. Purif. Rev..

[51] Wong K.K., Jawad Z.A. (2019). A review and future prospect of polymer blend mixed matrix membrane for CO_2_ separation. J. Polym. Res..

[52] Rezac M.E., Sorensen E.T., Beckham H.W. (1997). Transport properties of crosslinkable polyimide blends. J. Memb. Sci..

[53] Sabu T., Anil K.S., Runcy W., Soney C.G. (2018). Transport Properties of Polymeric Membranes.

[54] Freeman B., Yampolskii Y., Pinnau I. (2006). Materials Science of Membranes for Gas and Vapor Separation Materials.

[55] Kanehashi S., Nagai K. (2005). Analysis of dual-mode model parameters for gas sorption in glassy polymers. J. Membr. Sci..

[56] Miandoab E.S., Kentish S.E., Scholes C.A. (2021). Modelling competitive sorption and plasticization of glassy polymeric membranes used in biogas upgrading. J. Memb. Sci..

[57] Park H.B., Kamcev J., Robeson L.M., Elimelech M., Freeman B.D. (2017). Maximizing the right stuff: The trade-off between membrane permeability and selectivity. Science.

[58] Wu Y., Guo Z., Wu H., Zhu K., Yang L., Younas M., Jiang Z. (2020). Plasticization- and aging-resistant membranes with venation-like architecture for efficient carbon capture. J. Memb. Sci..

[59] Wypych G. (2017). Handbook of Plasticizer.

[60] Minelli M., Oradei S., Fiorini M., Sarti G.C. (2019). CO_2_ plasticization effect on glassy polymeric membranes. Polymer.

[61] Sridhar S., Bee S., Bhargava S. (2014). Membrane-based Gas Separation: Principle, Applications and Future Potential. Chem. Eng. Dig..

[62] Genduso G., Ghanem B.S., Pinnau I. (2019). Experimental mixed-gas permeability, sorption and diffusion of CO_2_–CH_4_ mixtures in 6FDA-mPDA polyimide membrane: Unveiling the effect of competitive sorption on permeability selectivity. Membranes.

[63] Freeman B.D., Pinnau I. (2004). Gas and Liquid Separations Using Membranes: An Overview. Advanced Materials for Membrane Separations.

[64] Liu Y., Liu Z., Morisato A., Bhuwania N., Chinn D., Koros J. (2020). Natural gas sweetening using a cellulose triacetate hollow fiber membrane illustrating controlled plasticization benefits. J. Memb. Sci..

[65] Bos A., Pünt I.G.M., Wessling M., Strathmann H. (1999). CO_2_-induced plasticization phenomena in glassy polymers. J. Memb. Sci..

[66] Ismail A.F., Lorna W. (2002). Penetrant-induced plasticization phenomenon in glassy polymers for gas separation membrane. Sep. Purif. Technol..

[67] Ahmad F., Lau K.K., Shariff A.M., Yeong Y.F. (2013). Temperature and pressure dependence of membrane permeance and its effect on process economics of hollow fiber gas separation system. J. Memb. Sci..

[68] Ricci E., Benedetti F.M., Dose M.E., De Angelis M.G., Freeman B.D., Paul D.R. (2020). Competitive sorption in CO_2_/CH_4_ separations: The case of HAB-6FDA polyimide and its TR derivative and a general analysis of its impact on the selectivity of glassy polymers at multicomponent conditions. J. Memb. Sci..

[69] Neyertz S., Brown D. (2020). Single- and mixed-gas sorption in large-scale molecular models of glassy bulk polymers. Competitive sorption of a binary CH_4_/N_2_ and a ternary CH_4_/N_2_/CO_2_ mixture in a polyimide membrane. J. Memb. Sci..

[70] He Z., Wang K. (2018). The “ideal selectivity” vs. “true selectivity” for permeation of gas mixture in nanoporous membranes. IOP Conference Series: Materials Science and Engineering.

[71] Huang Y., Paul D.R. (2005). Effect of temperature on physical aging of thin glassy polymer films. Macromolecules.

[72] Qian Q., Asinger P.A., Lee M.J., Han G., Mizrahi Rodriguez K., Lin S., Benedetti F.M., Wu A.X., Chi W.S., Smith Z.P. (2020). MOF-Based Membranes for Gas Separations. Chem. Rev..

[73] Yong W.F., Kwek K.H.A., Liao K.S., Chung T.S. (2015). Suppression of aging and plasticization in highly permeable polymers. Polymer.

[74] Yavari M., Le T., Lin H. (2017). Physical aging of glassy per fl uoropolymers in thin fi lm composite membranes. Part I. Gas transport properties. J. Memb. Sci..

[75] Houben H.J.M., Borneman Z., Nijmeijer K. (2020). Plasticization behavior of crown-ether containing polyimide membranes for the separation of CO_2_. Sep. Purif. Technol..

[76] Visser T., Koops G.H., Wessling M. (2005). On the subtle balance between competitive sorption and plasticization effects in asymmetric hollow fiber gas separation membranes. J. Memb. Sci..

[77] Mazinani S., Ramezani R., Molelekwa G.F., Darvishmanesh S., Di R., Bruggen B. (2019). Van Der Plasticization suppression and CO_2_ separation enhancement of Matrimid through homogeneous blending with a new high performance polymer. J. Memb. Sci..

[78] Lau C.H., Nguyen P.T., Hill M.R., Thornton A.W., Konstas K., Doherty C.M., Mulder R.J., Bourgeois L., Liu A.C.Y., Sprouster D.J. (2014). Ending aging in super glassy polymer membranes. Angew. Chemie-Int. Ed..

[79] Dong X., Liu C., Tran H. (2021). High Selectivity Membranes for Hydrogen Sulfide and Carbon Dioxide Removal from Natural Gas. U.S. Patent.

[80] Clarizia G., Tasselli F., Bernardo P. (2020). Effect of Physical Aging on Gas Transport in Asymmetric Polyimide Hollow Fibers Prepared by Triple-Orifice Spinneret. Polymers.

[81] Budd P.M., McKeown N.B. (2010). Highly permeable polymers for gas separation membranes. Polym. Chem..

[82] Rezakazemi M., Sadrzadeh M., Matsuura T. (2018). Thermally stable polymers for advanced high-performance gas separation membranes. Prog. Energy Combust. Sci..

[83] Adewole J.K., Sultan A.S. (2019). Polymeric Membranes for Natural Gas Processing: Polymer Synthesis and Membrane Gas Transport Properties. Functional Polymers, Polymers and Polymeric Composites.

[84] Ramachandran R., Jung D., Spokoyny A.M. (2019). Cross-linking dots on metal oxides. NPG Asia Mater..

[85] Achoundong C.S.K., Bhuwania N., Burgess S.K., Karvan O., Johnson J.R., Koros W.J. (2013). Silane Modi fi cation of Cellulose Acetate Dense Films as Materials for Acid Gas Removal. Macromolecules.

[86] Petrovic B., Gorbounov M., Soltani S.M. (2021). Microporous and Mesoporous Materials Influence of surface modification on selective CO_2_ adsorption: A technical review on mechanisms and methods. Microporous Mesoporous Mater..

[87] Li T., Liu J., Zhao S., Chen Z., Huang H., Guo R., Chen Y. (2019). Microporous polyimides containing bulky tetra- o -isopropyl and naphthalene groups for gas separation membranes. J. Memb. Sci..

[88] Wang Z., Shen Q., Liang J., Zhang Y., Jin J. (2020). Adamantane-grafted polymer of intrinsic microporosity with fi nely tuned interchain spacing for improved CO_2_ separation performance. Sep. Purif. Technol..

[89] Rahmani M., Kazemi A., Talebnia F., Gamali P.A. (2016). Fabrication and characterization of brominated separation: Application of response surface methodology (RSM). e-Polymers.

[90] Guiver M.D., Robertson G.P., Yoshikawa M. (2000). Functionalized Polysulfones: Methods for Chemical Modification and Membrane Applications. Membrane Formation and Modification.

[91] Koros W.J., Wallace D., Wind J.D., Miller S.J., Staudt-Bickel C. (2005). Crosslinked and Crosslinkable Hollow Fiber Membrane and Method of Making Same Utility. U.S. Patent.

[92] Zhang C., Li P., Cao B. (2017). Decarboxylation crosslinking of polyimides with high CO_2_/CH_4_ separation performance and plasticization resistance. J. Memb. Sci..

[93] Kratochvil A.M., Koros W.J. (2008). Decarboxylation-Induced Cross-Linking of a Polyimide for Enhanced CO_2_ Plasticization Resistance. Macromolecules.

[94] Wind J.D., Staudt-bickel C., Paul D.R., Koros W.J. (2002). The Effects of Crosslinking Chemistry on CO_2_ Plasticization of Polyimide Gas Separation Membranes. Ind. Eng. Chem. Res..

[95] Zhang C., Yan J., Tian Z., Liu X., Cao B., Li P. (2017). Molecular Design of Troger’s Base-Based Polymers Containing Spirobichroman Structure for Gas Separation. Ind. Eng. Chem. Res..

[96] Hu L., Cheng J., Li Y., Liu J., Zhou J., Cen K. (2018). In-situ grafting to improve polarity of polyacrylonitrile hollow fiber-supported polydimethylsiloxane membranes for CO_2_ separation. J. Colloid Interface Sci..

[97] Lin W., Chung T. (2001). Gas permeability, diffusivity, solubility, and aging characteristics of 6FDA-durene polyimide membranes. J. Membr. Sci..

[98] Niwa M., Kawakami H., Kanamori T., Shinbo T., Kaito A., Nagaoka S. (2001). Gas Separation of Asymmetric 6FDA Polyimide Membrane with Oriented Surface Skin Layer. Macromolecules.

[99] Suhaimi N.H., Yeong Y.F., Ch’ng C.W.M., Jusoh N. (2019). Tailoring CO_2_/CH_4_ Separation Performance of Mixed Matrix Membranes by Using ZIF-8 Particles Functionalized with Di ff erent Amine Groups. Polymers.

[100] Xu R., Li L., Hou M., Xue J., Liu Y., Pan Z., Song C., Wang T. (2020). Enhanced CO_2_ permeability of thermal crosslinking membrane via sulfonation/desulfonation of phenolphthalein-based cardo poly (arylene ether ketone). J. Memb. Sci..

[101] Largier T., Huang F., Kahn W., Cornelius C.J. (2019). Poly (phenylene) synthesized using diels-alder chemistry and its sulfonation: Sulfonate group complexation with metal counter-ions, physical properties, and gas transport. J. Memb. Sci..

[102] Liu C., Tran H. (2016). Polyimide Blend Membrane for Gas Separations. U.S. Patent.

[103] Xu R., Li L., Jin X., Hou M., He L., Lu Y., Song C., Wang T. (2019). Thermal crosslinking of a novel membrane derived from phenolphthalein- based cardo poly (arylene ether ketone) to enhance CO_2_/CH_4_ separation performance and plasticization resistance. J. Memb. Sci..

[104] Qiu W., Chen C., Xu L., Cui L., Paul D.R., Koros W.J. (2011). Sub- T g Cross-Linking of a Polyimide Membrane for Enhanced CO_2_ Plasticization Resistance for Natural Gas Separation. Macromolecules.

[105] Chen C., Miller S.J., Koros W.J. (2013). Characterization of Thermally Cross-Linkable Hollow Fiber Membranes for Natural Gas Separation. Ind. Eng. Chem. Res..

[106] Deng L., Xue Y., Yan J., Hon C., Cao B., Li P. (2019). Oxidative crosslinking of copolyimides at sub-T g temperatures to enhance resistance against CO_2_ -induced plasticization. J. Memb. Sci..

[107] Du N., Cin M.M.D., Pinnau I., Nicalek A., Robertson G.P., Guiver M.D. (2011). Azide-based Cross-Linking of Polymers of Intrinsic Microporosity (PIMs) for Condensable Gas Separation. Macromol. Rapid Commun..

[108] Liu Q., Galizia M., Gleason K.L., Scholes C.A., Paul D.R., Benny D. (2016). Influence of toluene on CO_2_ and CH_4_ gas transport properties in thermally rearranged (TR) polymers based on 3,3′-dihydroxy-4,4′-diamino-biphenyl (HAB) and 2,2′-bis-(3,4-dicarboxyphenyl) hexafluotopropane dianhydride (6FDA). J. Memb. Sci..

[109] Scholes C.A., Dong G., Sung J., Jin H., Lee J., Moo Y. (2017). Permeation and separation of SO2, H2S and CO_2_ through thermally rearranged (TR) polymeric membranes. Sep. Purif. Technol..

[110] Lee J., Sung J., Kim J.F., Jin H., Park H. (2019). Densification-induced hollow fiber membranes using crosslinked thermally rearranged (XTR) polymer for CO_2_ capture. J. Memb. Sci..

[111] Brunetti A., Cersosimo M., Sung J., Dong G., Fontananova E., Moo Y., Drioli E., Barbieri G. (2017). Thermally rearranged mixed matrix membranes for CO_2_ separation: An aging study. Int. J. Greenh. Gas Control.

[112] Li F.Y., Xiao Y., Ong Y.K., Chung T. (2012). UV-Rearranged PIM-1 Polymeric Membranes for Advanced Hydrogen Purifi cation and Production. Adv. Energy Mater..

[113] Altun V., Remigy J., Vankelecom I.F.J. (2017). UV-cured polysulfone-based membranes: E ff ect of co-solvent addition and evaporation process on membrane morphology and SRNF performance. J. Memb. Sci..

[114] Djoko T., Puji D., Ryan I. (2018). Enhancement of separation performance of nano hybrid PES–TiO_2_ membrane using three combination e ff ects of ultraviolet irradiation, ethanol-acetone immersion, and thermal annealing process for CO_2_ removal. J. Environ. Chem. Eng..

[115] Park C., Chang B., Kim J., Moo Y. (2019). UV-crosslinked poly (PEGMA-co-MMA-co-BPMA) membranes: Synthesis, characterization, and CO_2_/N_2_ and CO_2_/CO separation. J. Memb. Sci..

[116] Sazanova T.S., Otvagina K.V., Vorotyntsev I.V. (2018). The contributions of supramolecular organization to mechanical properties of chitosan and chitosan copolymers with synthetic polymers according to atomic force microscopy. Polym. Test..

[117] Sazanova T.S., Otvagina K.V., Kryuchkov S.S., Zarubin D.M., Fukina D.G., Vorotyntsev A.V., Vorotyntsev I.V. (2020). Revealing the surface effect on gas transport and mechanical properties in nonporous polymeric membranes in terms of surface free energy. Langmuir.

[118] Vasagar V., Hassan M.K., Khraisheh M. (2021). Membrane surface modification and functionalization. Membranes.

[119] SpecialChem Polyethersulfone (PES)—Complete Guide on High-Temperature Engineering Polymer. https://omnexus.specialchem.com/selection-guide/polyethersulfone-pes-thermoplastic.

[120] Mannan H.A., Mukhtar H., Shaharun M.S., Othman M.R., Murugesan T. (2016). Polysulfone/poly (ether sulfone) blended membranes for CO_2_ separation. J. Appl. Polym. Sci..

[121] Mosleh S., Mozdianfard M.R., Hemmati M., Khanbabaei G. (2016). Synthesis and characterization of rubbery/glassy blend membranes for CO_2_/CH_4_ gas separation. J. Polym. Res..

[122] Kojabad M.E., Babaluo A., Tavakoli A. (2021). A novel semi-mobile carrier facilitated transport membrane containing aniline/poly (ether-block-amide) for CO_2_/N_2_ separation: Molecular simulation and experimental study. Sep. Purif. Technol..

[123] Tu Z., Liu P., Zhang X., Shi M., Zhang Z., Luo S. (2021). Highly-selective separation of CO_2_ from N_2_ or CH_4_ in task-specific ionic liquid membranes: Facilitated transport and salting-out effect. Sep. Purif. Technol..

[124] Kunalan S., Dey K., Kumar P., Velachi V., Kumar P., Palanivelu K., Jayaraman N. (2021). Efficient facilitated transport PETIM dendrimer-PVA-PEG/PTFE composite flat-bed membranes for selective removal of CO_2_. J. Memb. Sci..

[125] Sun J., Wang Y., Liu J., Xu Q., Yin J. (2021). Highly selective separation of CO_2_/N_2_ using [Emim][Tf2N] supported ionic liquid membranes prepared by supercritical fluid deposition. J. Supercrit. Fluids.

[126] Vijayakumar V., Kim J.H., Nam S.Y. (2021). Piperidinium functionalized poly(2,6 dimethyl 1,4 phenylene oxide) based polyionic liquid/ionic liquid (PIL/IL) composites for CO_2_ separation. J. Ind. Eng. Chem..

[127] Zhang X., Xiong W., Tu Z., Peng L., Wu Y., Hu X. (2019). Supported Ionic Liquid Membranes with Dual-Site Interaction Mechanism for Efficient Separation of CO_2_. ACS Sustain. Chem. Eng.

[128] Zhang X., Tu Z., Li H., Li L., Wu Y., Hu X. (2017). Supported protic-ionic-liquid membranes with facilitated transport mechanism for the selective separation of CO_2_. J. Memb. Sci..

[129] Zhang X., Tu Z., Li H., Huang K., Hu X. (2017). Selective separation of H_2_S and CO_2_ from CH_4_ by supported ionic liquid membranes. J. Memb. Sci..

[130] Halder K., Munir M., Grünauer J., Shishatskiy S., Abetz C. (2017). Blend membranes of ionic liquid and polymers of intrinsic microporosity with improved gas separation characteristics. J. Memb. Sci..

[131] Klemm A., Lee Y.Y., Mao H., Gurkan B. (2020). Facilitated Transport Membranes With Ionic Liquids for CO_2_ Separations. Front. Chem..

[132] Cheng Y., Wang Z., Zhao D. (2018). Mixed Matrix Membranes for Natural Gas Upgrading: Current Status and Opportunities. Ind. Eng. Chem. Res..

[133] Goh P.S., Ismail A.F., Sanip S.M., Ng B.C., Aziz M. (2011). Recent advances of inorganic fillers in mixed matrix membrane for gas separation. Sep. Purif. Technol..

[134] Alberto M., Bhavsar R., Luque-alled J.M., Vijayaraghavan A., Budd P.M., Gorgojo P. (2018). Impeded physical aging in PIM-1 membranes containing graphene-like fi llers. J. Memb. Sci..

[135] Aqilah N., Fauzan B., Mukhtar H., Nasir R. (2020). Composite amine mixed matrix membranes for high- pressure CO_2_–CH_4_ separation: Synthesis, characterization and performance evaluation. R. Soc. Open Sci..

[136] Chen X.Y., Tien-binh N., Romero A., Patón A., Sanchez-silva L., Valverde J.L., Kaliaguine S., Rodrigue D. (2020). Gas Separation Properties of Mixed Matrix Membranes Based on Polyimide and Graphite Oxide Graphical abstract Keywords. J. Membr. Sci. Res..

[137] Natarajan P., Sasikumar B., Elakkiya S., Arthanareeswaran G., Ismail A.F., Youravong W., Yuliwati E. (2021). Pillared cloisite 15A as an enhancement filler in polysulfone mixed matrix membranes for CO_2_/N_2_ and O_2_/N_2_ gas separation. J. Nat. Gas Sci. Eng..

[138] Roilo D., Checchetto R. (2017). Gas Transport Properties and Free Volume Structure of Polymer Nanocomposite Membranes. Ph.D. Thesis.

[139] Padbury R. (2013). Bulk Property Modification of Fiber Forming Polymers Using Vapor Phase.

[140] Abdulhamid M.A., Genduso G., Wang Y., Ma X., Pinnau I. (2020). Plasticization-Resistant Carboxyl-Functionalized 6FDA-Polyimide of Intrinsic Microporosity (PIM–PI) for Membrane-Based Gas Separation. Ind. Eng. Chem. Res..

[141] Ma X., Swaidan R., Belmabkhout Y., Zhu Y., Litwiller E., Jouiad M., Pinnau I., Han Y. (2012). Synthesis and Gas Transport Properties of Hydroxyl-Functionalized Polyimides with Intrinsic Microporosity. Macromolecules.

[142] Karimi S., Firouzfar E., Khoshchehreh M.R. (2019). Journal of Petroleum Science and Engineering Assessment of gas separation properties and CO_2_ plasticization of polysulfone/polyethylene glycol membranes. J. Pet. Sci. Eng..

[143] Yave W., Car A., Peinemann K. (2010). Nanostructured membrane material designed for carbon dioxide separation. J. Membr. Sci..

[144] Car A., Stropnik C., Yave W., Peinemann K. (2008). Pebax^®^/polyethylene glycol blend thin film composite membranes for CO_2_ separation: Performance with mixed gases. Sep. Purif. Technol..

[145] Kawakami M., Iwanaga H., Hara Y., Iwamoto M. (1982). Gas Permeabilities of Cellulose Nitrate/Poly (ethylene Glycol) Blend Membranes. J. Appl. Polym. Sci..

[146] Okamoto K., Fujii M., Okamyo S., Suzuki H., Tanaka K., Kita H. (1995). Gas Permeation Properties of Poly(ether imide) Segmented Copolymers. Macromolecules.

[147] Yoshino M., Ito K., Kita H., Okamoto K. (2000). Effects of Hard-Segment Polymers on CO_2_/N_2_ Gas- Separation Properties of Poly (ethylene oxide) -Segmented. J. Polym. Sci. Part B Polym. Phys..

[148] Yu Y., Ma Y., Yin J., Zhang C., Feng G., Zhang Y., Meng J. (2021). Tuning the micro-phase separation of the PES-g-PEG comb-like copolymer membrane for efficient CO_2_ separation. Sep. Purif. Technol..

[149] Lee J.H., Jung J.P., Jang E., Lee K.B., Kang Y.S., Kim J.H. (2016). CO_2_-philic PBEM-g-POEM comb copolymer membranes: Synthesis, characterization and CO_2_/N_2_ separation. J. Memb. Sci..

[150] Bazhenov S.D., Borisov I.L., Bakhtin D.S., Rybakova A.N., Khotimskiy V.S., Molchanov S.P., Volkov V. (2016). V High-permeance crosslinked PTMSP thin-film composite membranes as supports for CO_2_ selective layer formation. Green Energy Environ..

[151] Feng H., Hong T., Mahurin S.M., Mays J.W., Sokolov A.P., Kang N., Saito T. (2017). Gas separation mechanism of CO_2_ selective amidoxime-poly(1-trimethylsilyl-1-propyne) membranes. Polym. Chem..

[152] Yampolskii Y., Belov N., Alentiev A. (2020). Perfluorinated polymers as materials of membranes for gas and vapor separation. J. Memb. Sci..

[153] Chiang H., Fang M., Okamoto Y. (2020). Mechanical, optical and gas transport properties of poly (perfluoro-2- methylene-4-methyl-1, 3-dioxolane) membrane containing perfluoropolyether as a plasticizer. J. Fluor. Chem..

[154] Baker R.W., Pinnau I., He Z., Amo K.D., Costa A.R.D., Daniels R. (2003). Carbon Dioxide Gas Separation Using Organic-Vapor Resistant Membranes. U.S. Patent.

[155] Baker R.W., Pinnau I., He Z., Amo K.D., Costa A.R.D., Daniels R. (2003). Nitrogen Gas Separation Using Organic Vapor Resistant Membrane. U.S. Patent.

